# Differences in genome-wide gene expression response in peripheral blood mononuclear cells between young and old men upon caloric restriction

**DOI:** 10.1186/s12263-016-0528-0

**Published:** 2016-05-06

**Authors:** I. P. G. Van Bussel, A. Jolink-Stoppelenburg, C. P. G. M. De Groot, M. R. Müller, L. A. Afman

**Affiliations:** 1 Division of Human Nutrition, Wageningen University, Bomenweg 2, 6703 HD Wageningen, The Netherlands; 2 Current Address: Norwich Medical School, University of East Anglia, Norwich, NR4 7TJ UK; 3 Division of Human Nutrition, Wageningen University & Research centre, PO BOX 8129, NL-6700 EV Wageningen, The Netherlands

**Keywords:** Age, Caloric restriction, Gene expression, Microarray, Peripheral blood mononuclear cells

## Abstract

**Background:**

Caloric restriction (CR) is considered to increase lifespan and to prevent various age-related diseases in different nonhuman organisms. Only a limited number of CR studies have been performed on humans, and results put CR as a beneficial tool to decrease risk factors in several age-related diseases. The question remains at what age CR should be implemented to be most effective with respect to healthy aging. The aim of our study was to elucidate the role of age in the transcriptional response to a completely controlled 30 % CR diet on immune cells, as immune response is affected during aging. Ten healthy young men, aged 20–28, and nine healthy old men, aged 64–85, were subjected to a 2-week weight maintenance diet, followed by 3 weeks of 30 % CR. Before and after 30 % CR, the whole genome gene expression in peripheral blood mononuclear cells (PBMCs) was assessed.

**Results:**

Expression of 554 genes showed a different response between young and old men upon CR. Gene set enrichment analysis revealed a downregulation of gene sets involved in the immune response in young but not in old men. At baseline, immune response-related genes were higher expressed in old compared to young men. Upstream regulator analyses revealed that most potential regulators were controlling the immune response.

**Conclusions:**

Based on the gene expression data, we theorise that a short period of CR is not effective in old men regarding immune-related pathways while it is effective in young men.

**Trial registration:**

ClinicalTrials.gov, NCT00561145

**Electronic supplementary material:**

The online version of this article (doi:10.1186/s12263-016-0528-0) contains supplementary material, which is available to authorized users.

## Background

Caloric restriction (CR), the restriction of food intake without malnutrition, increases longevity in *Caenorhabditis elegans* [31], *Saccharomyces cerevisiae* [18], and rodents [32]. In addition to longevity, CR minimises the age-related dysfunction of organs [19] and lowers risks of several age-related diseases, for example, cancer in rats and mice [22], and age-related aorta sclerosis in rats [4]. CR studies in primates led to less conclusive results. CR did increase longevity in monkeys at the Wisconsin National Primate Research Centre [6] but did not increase longevity in monkeys at the National Institute of Aging [20]. Factors such as genetics, husbandry, or dietary composition are perhaps more relevant for longevity in these primate studies than the number of calories [20]. Despite contrasting longevity results, both studies documented beneficial health effects of CR, including improved immune function and improved glucose homeostasis [20]. The limited number of studies investigating the effect of a CR diet in humans is, because of long life expectancy [27], solely directed at beneficial health effects and not at longevity [15]. For example, 6 years of CR decreased risk factors for atherosclerosis in humans [11] and 1 year of CR decreased risk factors for coronary heart disease in humans [12]. Also, aging processes seem to be altered by CR: the gene expression profiles from skeletal muscles from humans of the CR Society showed a closer relationship to the gene expression profiles of young subjects than to those of age-matched subjects [21]. The preventive or retardative effect of CR on age-associated changes in gene expression has also been shown in the muscle, brain, heart, and adipose tissue from other species [23].

Mechanisms underlying beneficial effects of CR remain largely unclear. To understand these mechanisms, genes and molecular pathways involved in the effects of CR on longevity and healthy aging have been investigated. Overall, the effects of CR are characterised by the downregulated expression of genes involved in growth hormone signalling and genes involved in immune response [25]. In contrast, aging is characterised by the upregulated expression of genes involved in immune response [10]. The opposing effects of CR and aging on the immune response might be one potential lead for the beneficial effects of CR on healthy aging. In this regard, immune cells, such as peripheral blood mononuclear cells (PBMCs), are an interesting target to study in humans [13, 25]. PBMCs are easily accessible and circulate in the blood [2], exposing them to metabolites, hormones, chemokines, or cytokine from tissues such as the liver and adipose tissue [3], which make them relevant to study. So far, most human CR studies have been executed in middle-aged subjects; the question remains at what age CR should be implemented to be most effective with respect to healthy aging. To approach this question, we aimed to elucidate the effect of age in the response to CR by comparing whole genome gene expression response to 3 weeks of 30 % CR in PBMCs from old and young men.

## Results

Baseline characteristics for ten young and nine old men of which high quality microarrays were present are summarised in Table 1. Besides the lower body mass index (BMI, *P* = 0.04) and lower fasting glucose level (*P* < 0.001) in old compared to young men, no differences were observed.

**Table 1:** Baseline characteristics of young (*n* = 10) and old (*n* = 9) men of whom microarray analysis on PBMCs was performed. Data represent mean and (SD) or median and [range]

	Young men	Old men	*P* value
Age (year)	24 [20, 28]	70 [64, 85]	4.37E−09
Height (m)	1.78 (0.06)	1.77 (0.04)	5.26E−01
Weight (kg)	71.1 (8.52)	76.7 (7.4)	1.28E−01
Body mass index (kg/m^2^)	22.4 (2.3)	24.6 (2.0)	3.08E−02
Glucose (mmol/L)	4.5 [3.7, 5.1]	5.2 [4.8, 5.5]	5.17E−04
Haemoglobin (g/L)	9.4 (0.4)	9.3 (0.4)	5.69E−01
Haematocrit (%)	45 (2)	44 (2)	5.46E−1

Three weeks of CR resulted in a decrease in body weight and BMI in both groups (Table 2). Age had no effect on weight (*P* = 0.18) or BMI change (*P* = 0.18).

**Table 2:** Body weight and body mass index of young and old men before and upon 3 weeks of 30 % CR and significance (*P* value). Data represent mean with (SD)

		Before CR	Upon CR	*P* value
Weight (kg)	Young (*n* = 10)	71.1 (8.5)	68.7 (8.6)	2.87E−05
	Old (*n* = 9)	76.7 (7.4)	74.9 (7.4)	6.53E−05
Body mass index (kg/m^2^)	Young (*n* = 10)	22.4 (2.3)	21.6 (2.3)	3.64E−05
	Old 9 (*n* = 9)	24.6 (2.0)	24.0 (2.0)	4.21E−05

### Effect on gene expression: old versus young

**Fig. 1:**
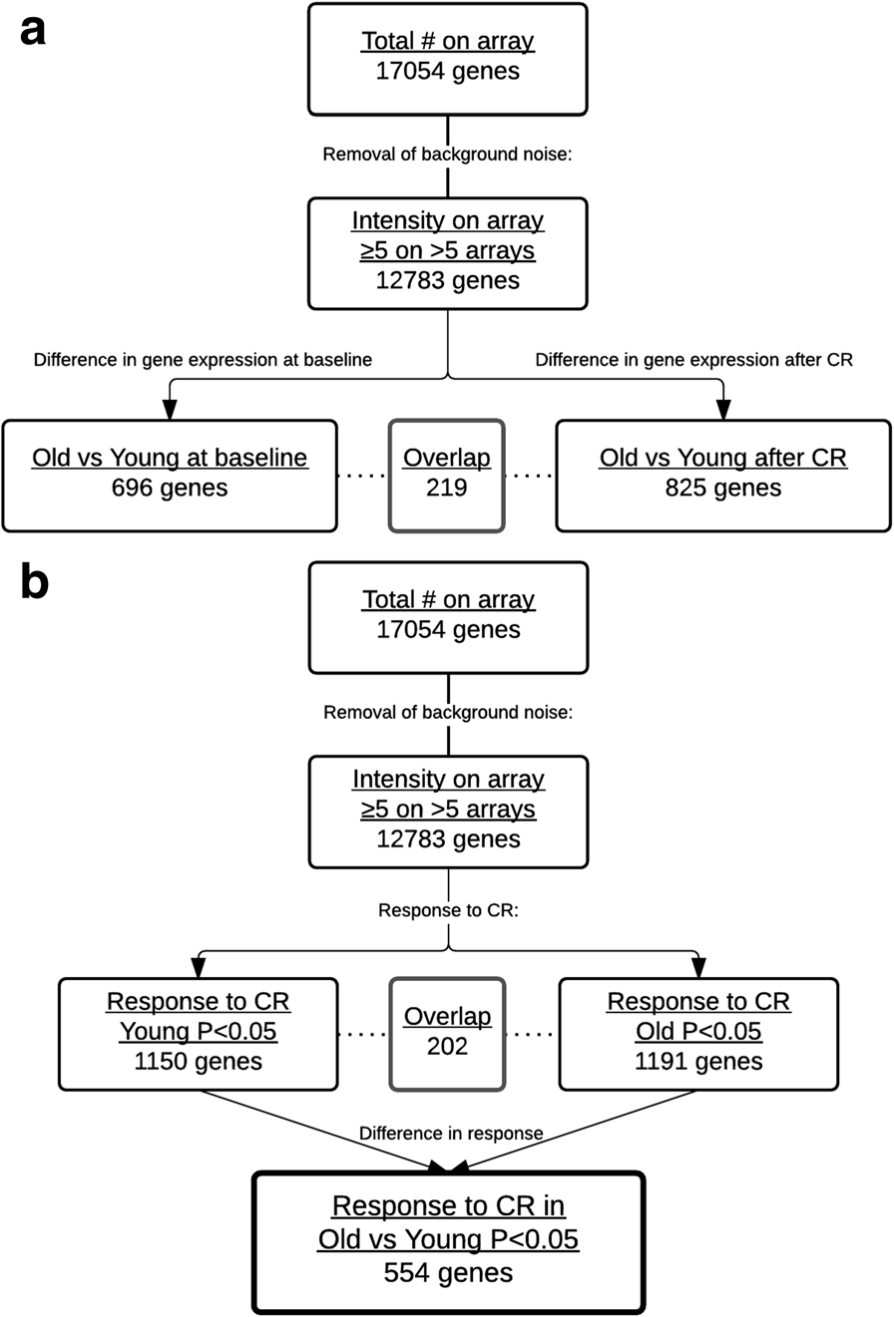
Stepwise selection of genes in microarray analysis of old versus young men upon 3 weeks of 30 % CR: 12,783 genes were selected for signal intensity (≥5 in >5 arrays), **a** for a difference in expression between old and young (*P* < 0.05) men at baseline (*left track*) and upon CR (*right track*), and **b** a change in expression of genes for young (*left track*) and old (*right track*) in response to CR. The *last box* depicts the number of genes that has a different response to CR in old versus young men

At baseline, before 30 % CR, 696 genes were significantly differently expressed between old and young men (Fig. 1a). To identify the effect of age on CR-induced gene expression changes, responses to CR were compared between old and young men (Fig. 1b). A total of 554 genes showed a significantly different expression response between old and young men.

### Effect of CR on gene expression: pathway analysis

Gene Set Enrichment Analysis (GSEA) was used to identify pathways in which gene expression was differentially regulated by age, at baseline, or in response to CR. Before the start of the 30 % CR diet, the expression of genes involved in immune response was higher in old compared to young men and the expression of genes involved in RNA processing was lower in old compared to young men (Table 3). Upon 3 weeks of 30 % CR, the expression of genes involved in immune response and glucose metabolism was downregulated in young men only, whereas the expression of genes involved in olfactory signalling was downregulated in old men only.

**Table 3:** Pathways changed in PBMC gene expression profiles of young and old men before and upon 3 weeks of 30 % CR

Pathway	Baseline (old vs young)	Young men	Old men
RNA processing	↓	↑	↑
Cell cycle	–	↑/↓^a^	↑/–^a^
Oxidative stress	–	↓	↓
Immune response	↑	↓	–
Glucose metabolism	–	↓	–
Olfactory signalling	–	–	↓

**↓** downregulated, ↑ upregulated, – no change

^a^Part of gene sets classified under these pathways were upregulated, whereas others where downregulated

### Upstream regulator analysis

**Fig. 2:**
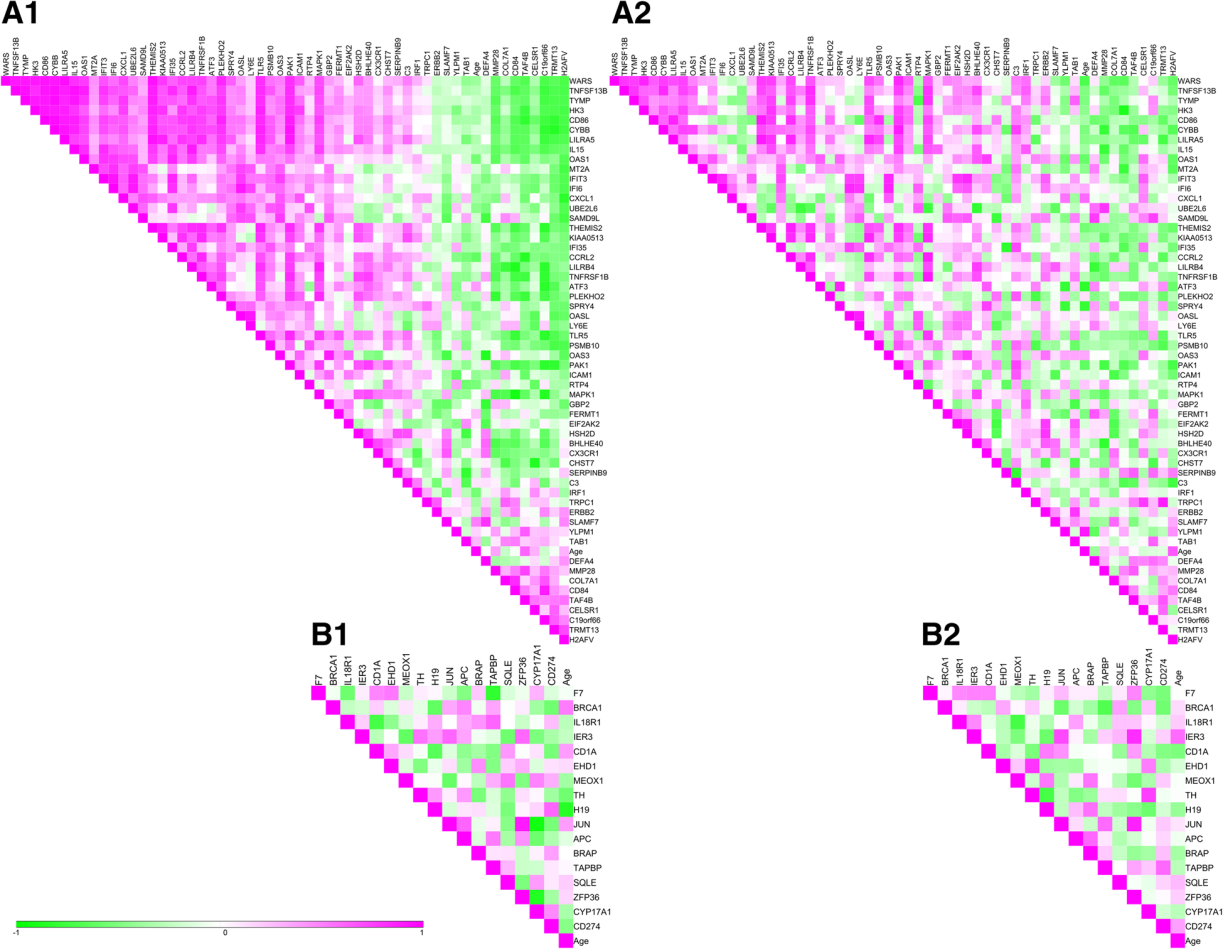
Correlation heat maps of CR-induced significant changes in gene expression of (*A*) genes regulated by upstream regulators in young, depicted for the response in young (*A1*) and old (*A2*), and of (*B*) genes regulated by upstream regulators in old, depicted for the response in young (*B1*) and old (*B2*). Scale: *green* = correlation score of −1, *pink* = correlation score of 1

Ingenuity Pathway Analysis (IPA) Upstream Regulator Analysis is a tool to find transcription regulators that may explain the observed gene expression. To identify these upstream regulators of genes that had a different expression before CR between old and young, or had a changed expression upon CR, we performed IPA Upstream Regulator Analysis. The regulators that were predicted to be affected at baseline and upon CR are listed in Additional file 1: Table S1. This list shows immune-related upstream regulators, interferon lambda 1 (INFL1), interferon alpha 2 (IFNA2), and interferon gamma (IFNG), that were predicted to be significantly higher in old compared to young before intervention. IFNA2 and IFNG were inhibited upon CR in young men, but not in old men. To identify correlation between the genes predicted to be regulated, we selected all significantly changed genes targeted by the predicted transcriptional regulators upon CR and created correlation heat maps of the significant changes in expression of these genes for young men (Fig. 2 (A1)) and old men (Fig. 2 (A2)). For young men, 57 unique genes were affected by the six identified transcriptional regulators, i.e. IFNA2, IFNG, eukaryotic translation initiation factor 2-alpha kinase 2 (EIF2AK2), mitogen-activated protein kinase 1 (MAPK1), glyceraldehyde-2-phosphate dehydrogenase (GAPDH), and transglutaminase 2 (TGM2) (Additional file 1: Table S1). These genes showed a distinct correlation in young men (Fig 2 (A1)) which was less strong or absent in old men (Fig 2 (A2)). Contrary for old men, many genes regulated by the upstream regulators were overlapping: 15 potential upstream regulators (Additional file 1: Table S1) were predicted to affect 17 unique genes, and no specific correlation pattern for old could be identified (Fig. 2 (B)).

### Younger transcriptional profile upon CR

**Fig. 3:**
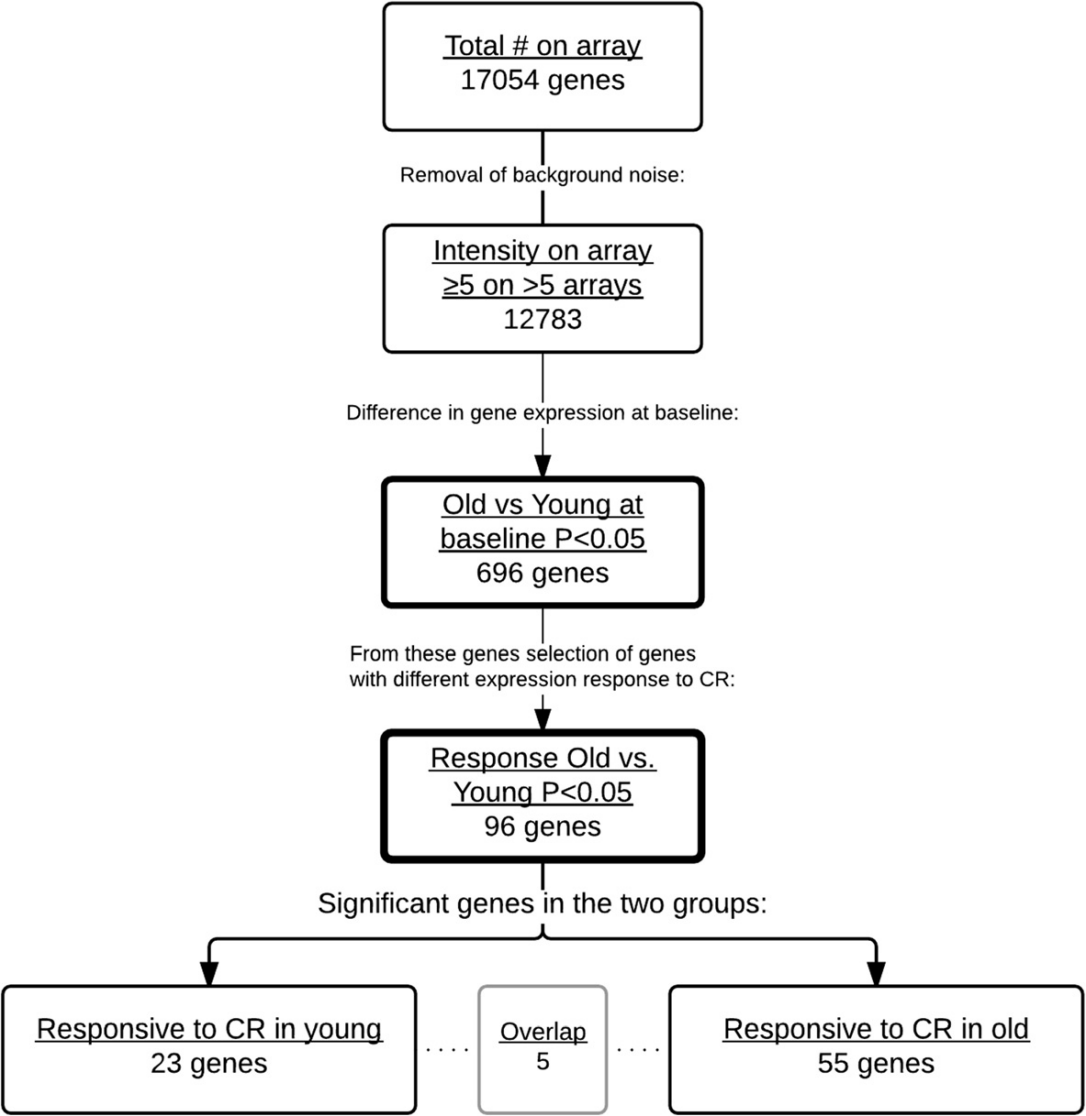
Stepwise selection of genes in microarray analysis to identify genes in which expression changed from an old profile to a young profile upon 3 weeks of 30 % CR. For signal intensity, 12,783 genes were selected (≥5 in >5 arrays); from these, 96 genes were selected that were differently expressed between old and young men before CR (*P* < 0.05); the last part depicts number of genes that show a different response to CR in old versus young men. Finally, 23 genes with a significant change in expression within young (*left track*) men and 55 genes within old (*right track*) men are shown

**Fig. 4:**
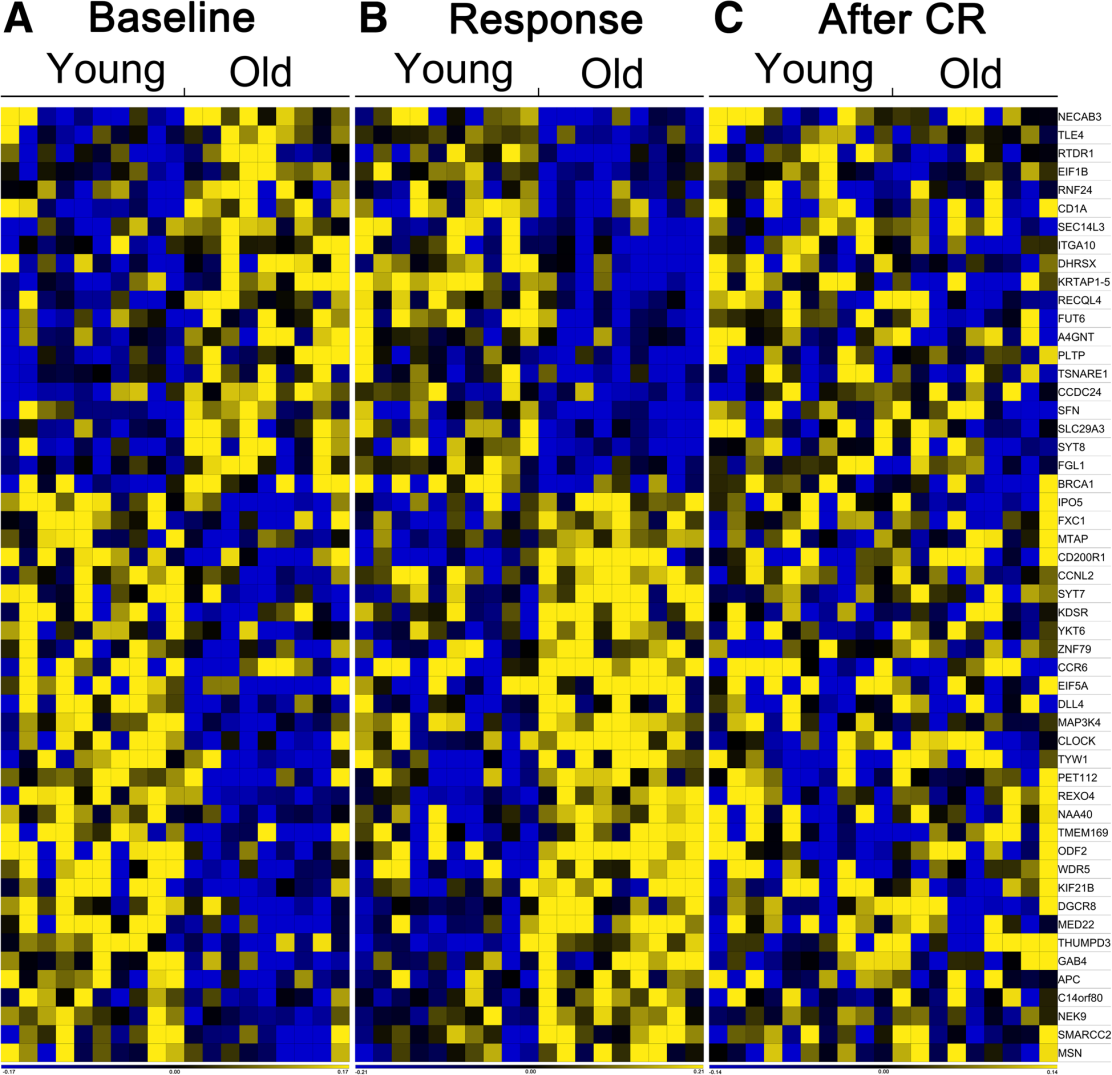
Heat map of genes of which expression changed from an old profile to a young profile upon CR. (**a**) baseline expression level, (**b**) response to CR, and (**c**) expression levels after CR for young and old men. Each column represents one person; each row represents one gene. Depicted are for (**a**) the signal log ratio calculated as gene expression values at baseline compared to the average of the whole group, for (**b**) the signal log ratios calculated as gene expression values upon CR compared to gene expression values at baseline, and for (**c**) the signal log ratios as gene expression values after CR compared to the average of the whole group.

To identify if old men were able to obtain, at least for a subset of genes, a younger gene expression profile upon CR, the following approach was used: selection of genes with a different expression between old and young before CR resulting in 696 genes (Fig. 3); 96 of these genes also showed a significant different response upon CR between old and young men (Fig. 3); 55 of these genes had a changed expression in old men only. Figure 4 shows a heat map of the gene expression per subject and illustrates the different expressed genes at baseline and the change towards a young profile in old men, as is shown by the third part of the heat map where no significant expression differences between old and young upon CR were present.

## Discussion

We aimed to investigate the potential relevance of age at which CR should be implemented to be most effective on gene expression changes of pathways important for healthy aging. To achieve this, we compared the gene expression changes in PBMCs of old men with the gene expression changes in PBMCs of young men upon 3 weeks of 30 % CR.

Three weeks of CR resulted in a downregulated expression of genes involved in immune response and glucose metabolism in young but not in old men. Effects of CR on immune response-related gene expression have been shown before; 8 weeks of CR in middle-aged obese men downregulated the expression of genes involved in immune response in PBMCs [7]. The response of these middle-aged obese men is similar to the response of young men in our study. One reason why we do not see the response in old men might be due to ‘immunosenescence’ [24], known to take place in elderly individuals above 65 years of age. Immunosenescence means the loss of immune functions and is characterised by an increase in the expression of inflammation- and immune response-related genes [10]. Although the overlap between the genes in this paper and in the current study is minimal, we also observed an increase in gene expression and a predicted activation of transcriptional regulators involved in immune pathways, i.e. IFNA2 and IFNG, in old men at baseline when compared to young. Upon CR, a decreased activation of IFNA2 and IFNG was only observed in young men. MAPK1 was activated upon CR. MAPK1 represses the expression of IFNG-induced genes via DNA-binding [16]. An increase in MAPK1 might have affected the decrease in IFNG-induced genes. The potential immunosenescence present at baseline may be the reason why we do not see a response on immune-related pathways upon CR in the old men. This is further illustrated by the correlation heat maps of immune-related genes in which high correlations are observed between gene expression responses in young men and far less pronounced effects are observed in old men. Old men seem to have lost the ability to change gene expression in immune response upon CR. This inability to change expression might be due to a potential advanced aging-related state of epigenetics, keeping the DNA structure in a more rigid structure and making it less likely to change gene expression. Either 3 weeks of 30 % CR is not sufficient to reduce the higher gene expression of immune-related genes in old men or the age-related potential epigenetic changes are too strong to overcome with CR and CR should be started at an earlier age. Three weeks of CR also resulted in a downregulated expression of genes involved in glucose metabolism in young men only. We did not find a difference in the expression of genes involved in glucose metabolism at baseline between old and young men, even though aging is known to have a diminishing effect on adequate glucose metabolism [9]. Aging might, however, have played a role in the nonresponsive effect of our short-term CR on glucose metabolism-related pathways in old men.

The decreased expression of genes related to olfactory signalling pathways in old men has not been described in the literature. However, other studies in fruit flies have been done in which the absence of odorants from nutrients affected the expression of odorant-binding proteins [26]. In addition, it has been described before in yeast that CR has an increasing effect on the expression of genes involved in RNA processing [5] as seen in our study for both young and old men.

Although old men did not respond with the same changes on immune response and glucose metabolism, many genes did show a change in expression upon CR. We identified a group of genes that changed from an ‘old’ expression level towards a ‘young’ expression level upon CR. This was in line with the finding that the expression of genes from the skeletal muscle tissue of middle-aged subjects of the Caloric Restriction Society matched closer to gene expression profiles of younger subjects than to gene expression profiles of age-matched controls [21]. We could, however, not find any clear signalling route, pathway, or network for these genes.

It should be mentioned that both baseline differences and differences in gene expression changes between young and old can be due to different subpopulations of immune cells in the PBMCs between the groups. During aging, involution of the thymus, responsible for production of naïve T cells, leads to a shift in the T cell population [14]. Unfortunately, we did not have the opportunity to determine PBMC subpopulations. Furthermore, a period of 3 weeks of CR is short and might not have been long enough to induce changes in the gene expression of old men. A strength of our study design is the completely controlled dietary run-in period of 2 weeks and the completely controlled dietary 30 % CR intervention of 3 weeks which excludes a potential effect of habitual diet differences between the young and old men on gene expression differences at baseline and on gene expression response upon CR.

## Conclusions

In our study, the expression of genes involved in immune response pathways was higher in old compared to young men at baseline. Three-week 30 % CR did not affect this higher immune-related gene expression in old men whereas it did reduce immune-related gene expression in young men. Due to our small sample size, we cannot draw solid conclusions about the relevance of age on the effect of CR on gene expression changes of pathways important for healthy aging. We hypothesise based on immune-related gene expression changes in men that for a short period of 30 % CR a young onset has more potential benefit than an old onset.

## Methods

### Study population and eligibility criteria

**Fig. 5:**
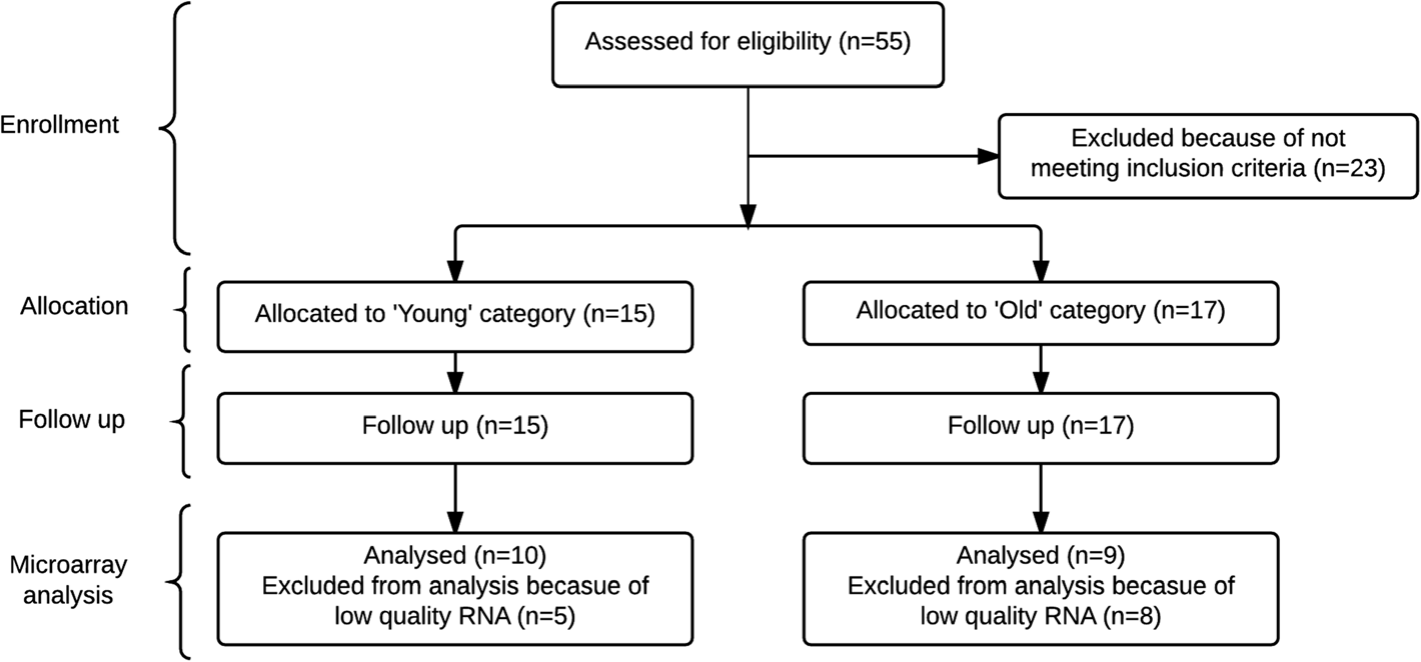
Flow diagram of subject inclusion

Our study population was a subgroup of participants who participated in a previously reported controlled-feeding trial [34]. Male Caucasian participants were recruited, by publishing advertisements in local newspapers and by sending out general e-mails to an e-mail list of persons who had indicated their interest in participating in studies of our university, at Wageningen University (The Netherlands), in October 2007 till January 2008, and followed up until the 15th of March 2008. Participants were excluded based on the following criteria: body mass index (BMI, kg/m^2^) less than 20 or higher than 30, adherence to a weight-reduction or medically prescribed diet, dementia (Mini-Mental State Examination score <21), diabetes, anaemia, gastrointestinal disorders, use of drugs known to interfere with energy balance, or a history of medical or surgical events known to affect the study outcome. Participants were divided based on their age into young (20–40 years) and old (65–85 years) men. Based on these criteria, 15 young and 17 old men were included in the original study at Wageningen University (The Netherlands) [34]. Microarray analyses were performed on high-quality PBMC RNA of ten young men, age range 20–28 years, and nine old men, age range 64–85 years (Fig. 5).

Each of the participants was informed about the design and purpose of the study, and each of the participants provided written informed consent. The Medical Ethical Committee of Wageningen University (The Netherlands) approved the study. This clinical study was registered with ClinicalTrials.gov as NCT00561145.

### Study design

The original study consisted of three subsequent phases as described previously [34] and was all carried out at the Division of Human Nutrition of Wageningen University (The Netherlands). Only samples collected after phases 1 and 2 are the subject of the current paper. Phase 1 (days 1–14): controlled dietary intervention in which each of the participants had to remain weight stable. Each of the participants was provided with a diet containing approximately 90 % of their estimated total daily energy requirement. The remaining 10 % was chosen from a list of choice items. In phase 2 (days 15–35): each of the participants was provided with a diet containing 70 % of the energy consumed during the last 3 days of phase 1. Composition of the diets was determined as described in [34]. Blood samples were taken at the end of phase 1, before CR, and at the end of phase 2, after CR.

### PBMC RNA isolation and microarray processing

PBMCs were isolated from whole blood using BD Vacutainer® Cell Preparation Tubes™ according to the manufacturer’s instructions. Total RNA was isolated from PBMC samples using TRIzol reagent (Invitrogen, Breda, The Netherlands) and purified using Qiagen RNeasy Micro Kit (Qiagen, Venlo, The Netherlands). RNA integrity was checked with Agilent 2100 Bioanalyzer (Agilent Technologies, Amstelveen, The Netherlands). Total RNA (500 ng/sample) was labelled using a one-cycle cDNA labelling kit (MessageAmpTM II-Biotin Enhanced Kit; Ambion Inc., Nieuwerkerk aan de IJssel, The Netherlands) and hybridised to human whole genome GeneChip arrays encoding 17,054 genes, designed by the European Nutrigenomics Organization and manufactured by Affymetrix (Santa Clara, CA). Sample labelling, hybridization to chips, and image scanning were performed according to the manufacturer’s instructions.

### Microarray data analysis

Quality control was performed and fulfilled the criteria for array hybridisation suggested by the Tumor Analysis Best Practices Working Group [30].

Microarrays were analysed using reorganised oligonucleotide probes as described by Dai et al. [8]. All individual probes for a gene were combined, allowing the possibility to detect overall transcription activity on the basis of latest genome and transcriptome information, rather than on the basis of Affymetrix probe set annotation. Expression values were calculated with the Robust Multi-array Average (RMA) method and quantile normalised [1, 17]. Only probe sets with normalised signals >5 on ≥5 arrays were defined as expressed and selected for analysis. This normalisation level was chosen because of a low microarray intensity level, due to the use of expired microarrays. It has, however, been shown that microarray data generated by microarrays more than 4 years past the manufacturer’s expiration date had lower signal intensities but were highly specific and consistent with those from unexpired microarrays [33]. We used microarrays within 2 years of the expiry date.

Individual genes were defined as changed when comparison of the average normalised signal intensities showed a *P* value <0.05 in a two-tailed paired *t* test with Bayesian correction (Limma) [29]. Filtered data were analysed with Gene Set Enrichment Analysis (GSEA; GSEA/MSigDB website v3.87 released April 4, 2013). Significantly regulated gene sets were defined with a false discovery rate of <0.25. Gene sets were visualised and clustered using Cytoscape [28], which enabled the identification of clusters of gene sets. Ingenuity Pathway Analysis version 8.5 (Ingenuity Systems, Redwood City, CA) was also used for pathway analysis and upstream regulator analysis, but because of similar results, only GSEA results are displayed. Ingenuity Pathway Analysis has been performed based on findings from human experiments*.*

For upstream regulator analysis at baseline, genes with a significant different expression at baseline were included (*P* < 0.05). For young, genes with a significant different response upon CR in young were included (*P* < 0.05), and for old, genes with a significant different response upon CR in old were included (*P* < 0.05). For correlation heat maps, target genes of the upstream regulators were included if they also had a significantly different response between old and young (*P* < 0.05) upon CR. Array data have been submitted to Gene Expression Omnibus under accession number GSE63117.

### Statistical analysis of clinical measurements

The statistical package SPSS (version 15.0; SPSS Inc, Chicago, IL) was used for analysis of the following data: expression changes within age groups were determined by paired *t* tests, and differential changes between age groups were determined by unpaired *t* tests.

### Availability of supporting data

The data set supporting the results of this article is available in the Gene Expression Omnibus repository, under accession number GSE63117, at http://www.ncbi.nlm.nih.gov/geo/. Additional data can be found in supplemental files. Additional file 2 contains the Quality Control report, Additional file 3 contains gene expression analysis, Additional file 4 contains GSEA outputs, and Additional file 5 contains IPA outputs.

## Additional files

Additional file 1: Table S1. Predicted upstream regulators. Predicted difference before CR between old and young, in the response of young, the response in old, and the difference upon CR between old and young. (PDF 106 kb) Additional file 2: Complete quality control report of microarrays. (PDF 3939 kb) Additional file 3: Gene expression file with expression analysis (A) baseline, (B) response to intervention in old men compared to response to intervention in young men, and (C) genes in old towards a young expression profile. (ZIP 4761 kb) Additional file 4: Gene Set Enrichment Analysis outputs for (A) old men compared to young men before intervention, (B) response to intervention in young men, (C) response to intervention in old men, and (D) response to intervention in old men compared to response to intervention in young men. (ZIP 75 kb) Additional file 5: Ingenuity Pathway Analysis Upstream regulators output used for Additional file 1: Table S1 (A) baseline, (B) response to intervention in young men, and (C) response to intervention in old men. (ZIP 27 kb)

## Abbreviations

BMI: body mass index
CR: caloric restriction
GSEA: Gene Set Enrichment Analysis
PBMCs: peripheral blood mononuclear cells
RMA: Robust Multi-Array Average
