# Supplementary material for: Differences in genome-wide gene expression response in peripheral blood mononuclear cells between young and old men upon caloric restriction
Source: Genes Nutr. 2016 May 6;11:13. doi: 10.1186/s12263-016-0528-0 (PMC4968441; doi:10.1186/s12263-016-0528-0)
Supplement: Additional file 1: Table S1. — Predicted upstream regulators. Predicted difference before CR between old and young, in the response of young, the response in old, and the difference upon CR between old and young. (PDF 106 kb) [file 12263_2016_528_MOESM1_ESM.pdf]

Upstream regulator	Function		Baseline (Old vs Young)			Young			Old	
			z-score	p-value		z-score	p-value		z-score	p-value
**IFNL1**	Cytokine	↑	2.630	4.94E-03	-	-1.929	2.25E-03	-	-	-
**IFNA2**	Cytokine	↑	3.272	1.68E-04	↓	-3.138	1.08E-03	-	-	-
**IFNG**	Cytokine	↑	2.934	1.85E-05	↓	-2.052	1.40E-02	-	0.792	3.48E-02
**EIF2AK2**	Kinase	-	-	-	↓	-2.828	1.10E-02	-	-	-
**MAPK1**	Kinase	↓	-2.985	1.12E-01	↑	3.63	1.19E-02	-	-	-
**GAPDH**	Enzyme	-	-	-	↑	2.433	6.81E-03	-	-	-
**TGM2**	Enzyme	-	0.112	1.11E+00	↓	-4.375	1.23E-03	-	-	-
**PDGF BB**	Complex	-	-	-	-	-	-	↓	-4.026	5.16E-09
**ERK**	Group	-	-	-	-	-	-	↓	-3.538	7.29E-04
**CCL5**	Cytokine	-	-	-	-	-	-	↓	-2.985	6.15E-04
**Jnk**	Group	-	-	-	-	-	-	↓	-2.960	3.71E-03
**F7**	Peptidase	-	-	-	-	-	-	↓	-2.772	9.95E-05
**CSF2**	Cytokine	-	-	-	-	-	-	↓	-2.722	3.14E-04
**STAT3**	Transcription regulator	-	-	-	-	-	-	↓	-2.598	1.19E-04
**ERK1/2**	Group	-	-	-	-	-	-	↓	-2.256	2.79E-03
**TNF**	Cytokine	-	0.870	8.23E-03	-	-1.993	0.261	↓	-2.219	5.28E-03
**P38 MAPK**	Group	-	-	-	-	-	-	↓	-2.131	1.21E-02
**EGF**	Growth factor	-	-	-	-	-	-	↓	-2.072	1.53E-02
**IL1**	Group	-	-	-	-	-	-	↓	-2.000	1.33E-02
**COL18A1**	Other	-	-	-	-	-	-	↑	3.000	1.70E-02
**CD24**	Other	-	-	-	↑	2.236	6.20E-02	↑	2.121	6.39E-03
**TAB1**	Enzyme	-	-	4.05E-02	-	-	-	↑	2.000	4.02E-02

↑: activated; ↓: inhibited; -: not activated/inhibited;

Black text: regulator is present in dataset and z-score is below -2 or above 2 with p-value<0.05

Grey text: regulator is present in dataset, but z-score is above -2 or below 2, or P-value is>0.05
