# Supplementary material for: Differences in genome-wide gene expression response in peripheral blood mononuclear cells between young and old men upon caloric restriction
Source: Genes Nutr. 2016 May 6;11:13. doi: 10.1186/s12263-016-0528-0 (PMC4968441; doi:10.1186/s12263-016-0528-0)
Supplement: Additional file 2: — Complete quality control report of microarrays. (PDF 3939 kb) [file 12263_2016_528_MOESM2_ESM.pdf]

Quality control report (BEFORE normalization only).
Date: Thu Mar 12 14:43:08 2009 / BioConductor version: 2.3 (R-2.8.0) / Platform: Linux / simpleaffy version: 2.18.0-1.
Scaling: target intensity (tgt) is: 100.000000

gapdh3 probeset is: AFFX-HUMGAPDH/M33197_3_at
gapdhM probeset is: AFFX-HUMGAPDH/M33197_M_at
gapdh5 probeset is: AFFX-HUMGAPDH/M33197_5_at
actin3 probeset is: AFFX-HSAC07/X00351_3_at
actinM probeset is: AFFX-HSAC07/X00351_M_at
actin5 probeset is: AFFX-HSAC07/X00351_5_at

Loaded Files	scaling factors	% present	average backgr.	minimum backgr.	maximum backgr.	BioB signal	BioB P/A	BioC signal	BioC P/A	BioD signal	BioD P/A	CreX signal	CreX P/A
A143_01_Nugo_001_t0.CEL	2.1495	50.3947	48.0235	47.1101	49.5859	7.4168	P	8.4722	P	11.0886	P	12.9850	P
A143_02_Nugo_001_t2.CEL	2.5512	49.8141	48.1705	46.8267	49.3229	7.4090	P	8.5548	P	11.1388	P	12.9470	P
A143_07_Nugo_009_t0.CEL	2.1543	50.7748	40.7014	39.9910	41.3852	7.5513	P	9.1998	P	11.2386	P	13.0359	P
A143_08_Nugo_009_t2.CEL	2.5776	51.0630	37.1145	36.6045	37.7534	7.7714	P	9.2425	P	11.3617	P	13.1824	P
A143_09_Nugo_014_t0.CEL	3.0626	50.0522	38.3634	37.6611	39.9203	7.3908	P	9.1260	P	11.1448	P	13.0149	P
A143_10_Nugo_014_t2.CEL	2.3495	52.1198	39.7032	38.2356	41.2707	7.4443	P	8.7598	P	11.1223	P	12.9897	P
A143_11_Nugo_016_t0.CEL	2.8158	49.3045	42.1085	41.3359	42.8909	7.7044	P	8.9225	P	11.4030	P	13.2560	P
A143_12_Nugo_016_t2.CEL	2.4472	51.2092	41.9470	41.2045	43.2242	7.8413	P	9.2037	P	11.4371	P	13.2431	P
A143_13_Nugo_017_t0.CEL	2.1896	51.2761	35.8791	34.8389	36.8565	7.2226	P	8.7476	P	10.8984	P	12.8316	P
A143_14_Nugo_017_t2.CEL	2.3848	52.3370	35.8437	35.0821	36.5620	7.5903	P	9.0734	P	11.1790	P	13.0825	P
A143_15_Nugo_018_t0.CEL	3.0276	49.6345	36.9492	36.0962	37.5654	7.5277	P	9.3712	P	11.3525	P	13.2429	P
A143_16_Nugo_018_t2.CEL	2.9039	49.6220	36.3773	35.3218	37.2682	7.5516	P	8.8208	P	11.2507	P	13.1673	P
A143_17_Nugo_019_t0.CEL	2.7441	51.1132	34.7151	34.1835	35.3203	7.2566	P	8.9009	P	10.9938	P	12.9217	P
A143_18_Nugo_019_t2.CEL	2.5826	52.3078	35.3372	34.8977	35.8429	7.8778	P	9.0459	P	11.2195	P	13.0644	P
A143_19_Nugo_020_t0.CEL	2.8834	50.2611	35.0814	34.2234	35.9474	7.5965	P	9.0703	P	11.3273	P	13.1654	P
A143_20_Nugo_020_t2.CEL	2.9752	51.6520	33.0459	32.2902	33.8647	7.9183	P	9.1975	P	11.4919	P	13.2701	P
A143_23_Nugo_024_t0.CEL	2.6615	50.2444	37.5929	36.6793	38.5022	7.9088	P	9.2162	P	11.4357	P	13.3011	P
A143_24_Nugo_024_t2.CEL	2.6927	50.9461	38.8579	38.3188	39.4469	7.7095	P	9.0976	P	11.4399	P	13.2736	P
A143_25_Nugo_029_t0.CEL	2.5585	50.1733	45.3953	44.0547	46.9761	7.3843	P	8.6801	P	10.9666	P	12.9027	P
A143_26_Nugo_029_t2.CEL	2.4735	49.6512	45.9500	45.3116	46.9504	7.2435	P	8.7951	P	11.0694	P	12.8769	P
A143_27_Nugo_030_t0.CEL	2.6258	50.1190	41.1955	39.9489	42.3068	7.7811	P	9.2376	P	11.3945	P	13.1589	P
A143_28_Nugo_030_t2.CEL	2.7885	48.6864	42.7352	41.1534	43.9318	7.5536	P	8.9579	P	11.1354	P	13.0270	P
A143_29_Nugo_031_t0.CEL	3.1050	47.9178	38.7291	35.9223	41.2827	7.5236	P	9.3209	P	11.3099	P	13.1805	P
A143_30_Nugo_031_t2.CEL	3.1655	48.3397	40.2332	39.0526	42.0571	7.8085	P	9.0582	P	11.4569	P	13.3561	P
A143_31_Nugo_033_t0.CEL	2.1096	52.3036	41.5372	40.5836	42.8012	7.2650	P	8.7725	P	10.9568	P	12.8153	P
A143_32_Nugo_033_t2.CEL	2.2786	52.3996	38.9115	38.0902	40.0897	7.2750	P	8.8578	P	11.1754	P	13.0500	P
A143_33_Nugo_038_t0.CEL	3.5358	47.6839	34.8696	34.0608	36.1504	7.6847	P	8.7890	P	11.4093	P	13.2093	P
A143_34_Nugo_038_t2.CEL	3.3185	48.9746	33.9198	33.4045	34.6211	7.7611	P	9.1833	P	11.4547	P	13.2613	P
A143_35_Nugo_039_t0.CEL	2.2624	51.7397	36.1624	35.5744	36.7728	7.4617	P	8.9025	P	11.1416	P	12.9365	P
A143_36_Nugo_039_t2.CEL	2.4253	51.9151	34.5033	33.4827	35.1643	7.1076	P	8.4026	P	10.8091	P	12.7330	P
A143_37_Nugo_044_t0.CEL	2.8838	49.6303	38.0244	37.1094	38.8744	7.6997	P	9.0704	P	11.2996	P	13.2010	P
A143_38_Nugo_044_t2.CEL	2.4637	51.5016	41.2042	39.7549	43.1729	7.9429	P	9.3830	P	11.3810	P	13.2545	P
A143_39_Nugo_045_t0.CEL	2.7639	50.4448	33.4874	33.0707	34.0226	7.3597	P	9.0303	P	11.0635	P	12.9760	P
A143_40_Nugo_045_t2.CEL	2.4508	52.8341	33.4695	32.5851	34.2767	7.5024	P	8.9632	P	11.1773	P	12.9857	P
A143_41_Nugo_048_t0.CEL	2.7024	50.0272	49.7338	47.8100	50.9669	7.4866	P	8.7250	P	10.8770	P	12.9748	P
A143_42_Nugo_048_t2.CEL	2.4093	50.8291	49.3120	48.2586	50.7788	7.3104	P	8.5469	P	11.0830	P	13.0065	P
A143_43A_Nugo_053_t0.CEL	3.1424	50.2861	35.5636	34.8857	36.1783	7.6948	P	8.9816	P	11.3102	P	13.2824	P
A143_44A_Nugo_053_t2.CEL	2.6417	51.5851	34.4253	33.8343	35.3797	7.7598	P	9.3110	P	11.3226	P	13.1709	P

Loaded Files	gapdh3 signal	gapdh3 P/A	gapdhM signal	gapdhM P/A	gapdh5 signal	gapdh5 P/A	actin3 signal	actin3 P/A	actinM signal	actinM P/A	actin5 signal	actin5 P/A
A143_01_Nugo_001_t0.CEL	12.1202	P	12.0310	P	12.0728	P	12.7878	P	12.6568	P	12.4038	P
A143_02_Nugo_001_t2.CEL	11.9499	P	11.7784	P	11.8649	P	12.9028	P	12.7584	P	12.7778	P
A143_07_Nugo_009_t0.CEL	11.9768	P	11.7782	P	11.8546	P	12.6617	P	12.4904	P	12.3162	P
A143_08_Nugo_009_t2.CEL	11.9478	P	11.8478	P	11.9531	P	12.8741	P	12.6057	P	12.4408	P
A143_09_Nugo_014_t0.CEL	11.9711	P	11.8770	P	11.7934	P	13.0437	P	12.8703	P	12.8039	P

A143_10_Nugo_014_t2.CEL	11.8995	P	11.7572	P	11.6978	P	12.7356	P	12.7014	P	12.5573	P
A143_11_Nugo_016_t0.CEL	11.8775	P	11.8318	P	11.8018	P	13.0130	P	12.6893	P	12.4761	P
A143_12_Nugo_016_t2.CEL	11.8782	P	11.7391	P	11.7114	P	12.8703	P	12.5767	P	12.2681	P
A143_13_Nugo_017_t0.CEL	12.0097	P	11.8793	P	11.9602	P	12.6890	P	12.4418	P	12.2694	P
A143_14_Nugo_017_t2.CEL	12.0767	P	12.0086	P	12.0183	P	12.8366	P	12.8939	P	12.6717	P
A143_15_Nugo_018_t0.CEL	11.5977	P	11.4273	P	11.5177	P	12.8617	P	12.5547	P	12.3048	P
A143_16_Nugo_018_t2.CEL	11.8229	P	11.4939	P	11.5690	P	12.8050	P	12.6127	P	12.4088	P
A143_17_Nugo_019_t0.CEL	12.0471	P	11.9764	P	11.8469	P	12.8354	P	12.7585	P	12.7039	P
A143_18_Nugo_019_t2.CEL	11.7850	P	11.6867	P	11.5465	P	12.7830	P	12.6166	P	12.5748	P
A143_19_Nugo_020_t0.CEL	11.7486	P	11.6001	P	11.4959	P	12.7396	P	12.5521	P	12.4005	P
A143_20_Nugo_020_t2.CEL	11.5631	P	11.3401	P	11.2044	P	12.7396	P	12.4984	P	12.2956	P
A143_23_Nugo_024_t0.CEL	11.8516	P	11.7170	P	11.7238	P	12.8906	P	12.6247	P	12.4687	P
A143_24_Nugo_024_t2.CEL	11.6194	P	11.4219	P	11.4792	P	12.8064	P	12.5609	P	12.3307	P
A143_25_Nugo_029_t0.CEL	11.8853	P	11.8653	P	11.8958	P	12.7805	P	12.6962	P	12.5548	P
A143_26_Nugo_029_t2.CEL	12.0477	P	11.9193	P	11.9821	P	12.8115	P	12.6463	P	12.5338	P
A143_27_Nugo_030_t0.CEL	11.7698	P	11.5732	P	11.5839	P	12.7140	P	12.5396	P	12.3671	P
A143_28_Nugo_030_t2.CEL	11.7907	P	11.7104	P	11.6069	P	12.7694	P	12.5678	P	12.4545	P
A143_29_Nugo_031_t0.CEL	11.9049	P	11.7304	P	11.6508	P	12.8231	P	12.6247	P	12.4323	P
A143_30_Nugo_031_t2.CEL	11.6123	P	11.4547	P	11.3121	P	12.9180	P	12.6015	P	12.5154	P
A143_31_Nugo_033_t0.CEL	11.7811	P	11.7615	P	11.8022	P	12.5427	P	12.6118	P	12.4330	P
A143_32_Nugo_033_t2.CEL	11.7637	P	11.6051	P	11.6686	P	12.8021	P	12.6409	P	12.4637	P
A143_33_Nugo_038_t0.CEL	11.8462	P	11.5815	P	11.6284	P	12.9800	P	12.6274	P	12.4651	P
A143_34_Nugo_038_t2.CEL	11.9515	P	11.6276	P	11.6036	P	12.9212	P	12.6116	P	12.3841	P
A143_35_Nugo_039_t0.CEL	11.8144	P	11.7561	P	11.7183	P	12.6978	P	12.6296	P	12.4981	P
A143_36_Nugo_039_t2.CEL	11.6107	P	11.5546	P	11.3204	P	12.6077	P	12.4667	P	12.2088	P
A143_37_Nugo_044_t0.CEL	11.7849	P	11.6525	P	11.6320	P	12.8556	P	12.5539	P	12.4327	P
A143_38_Nugo_044_t2.CEL	11.7491	P	11.5991	P	11.5725	P	12.8323	P	12.5590	P	12.2832	P
A143_39_Nugo_045_t0.CEL	11.8221	P	11.8121	P	11.5079	P	12.8630	P	12.6482	P	12.5164	P
A143_40_Nugo_045_t2.CEL	11.8308	P	11.7182	P	11.2960	P	12.7281	P	12.6248	P	12.3879	P
A143_41_Nugo_048_t0.CEL	11.8138	P	11.5684	P	11.3095	P	12.7772	P	12.6302	P	12.3019	P
A143_42_Nugo_048_t2.CEL	11.7360	P	11.5552	P	11.7339	P	12.7718	P	12.6710	P	12.5260	P
A143_43A_Nugo_053_t0.CEL	11.8744	P	11.7323	P	11.6267	P	12.8221	P	12.8242	P	12.5470	P
A143_44A_Nugo_053_t2.CEL	11.7422	P	11.6465	P	11.6827	P	12.7868	P	12.6433	P	12.5070	P

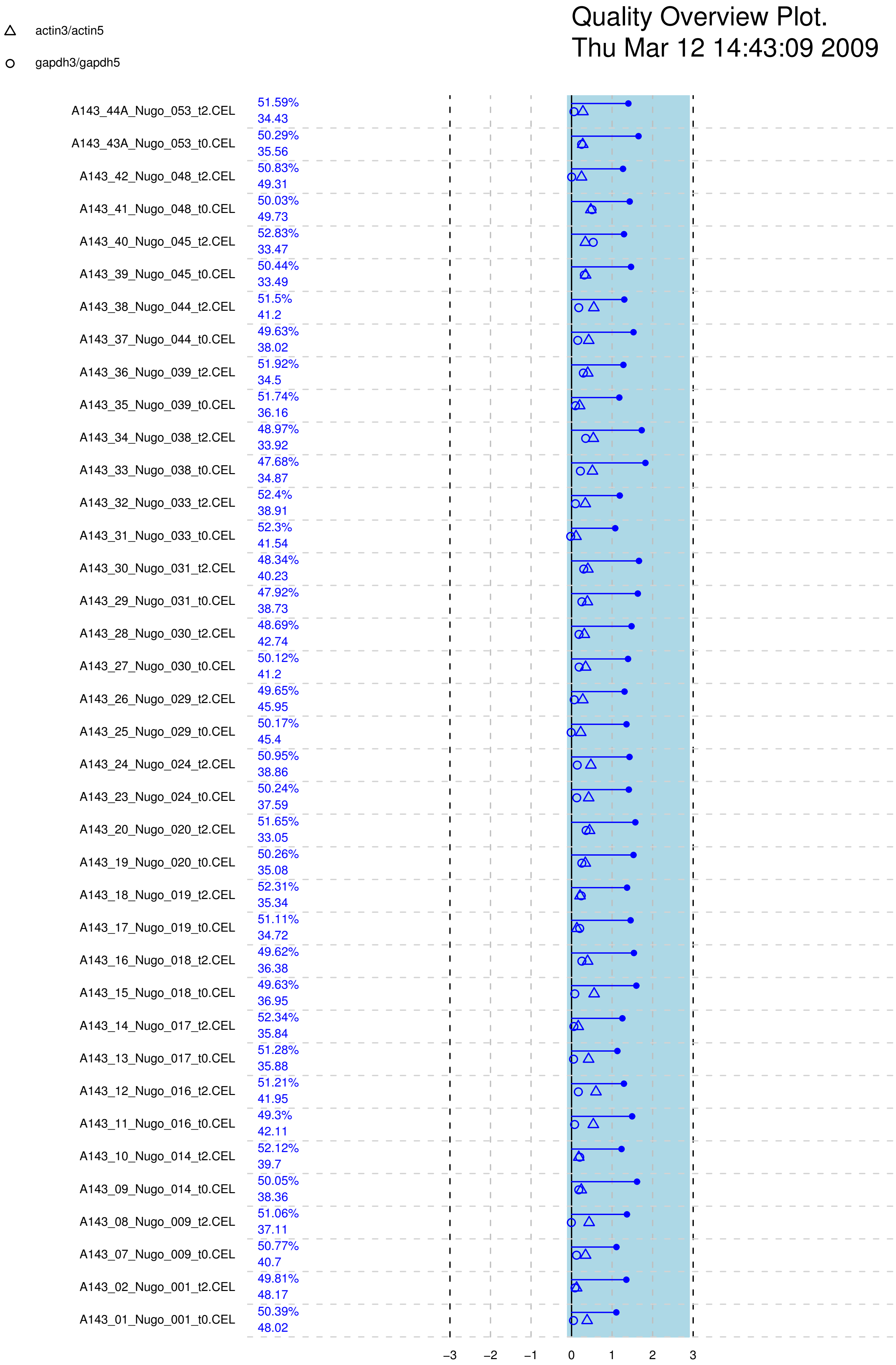
Quality Overview Plot.
Thu Mar 12 14:43:09 2009
actin3/actin5
gapdh3/gapdh5
A143_44A_Nugo_053_t2.CEL 51.59% 34.43
A143_43A_Nugo_053_t0.CEL 50.29% 35.56
A143_42_Nugo_048_t2.CEL 50.83% 49.31
A143_41_Nugo_048_t0.CEL 50.03% 49.73
A143_40_Nugo_045_t2.CEL 52.83% 33.47
A143_39_Nugo_045_t0.CEL 50.44% 33.49
A143_38_Nugo_044_t2.CEL 51.5% 41.2
A143_37_Nugo_044_t0.CEL 49.63% 38.02
A143_36_Nugo_039_t2.CEL 51.92% 34.5
A143_35_Nugo_039_t0.CEL 51.74% 36.16
A143_34_Nugo_038_t2.CEL 48.97% 33.92
A143_33_Nugo_038_t0.CEL 47.68% 34.87
A143_32_Nugo_033_t2.CEL 52.4% 38.91
A143_31_Nugo_033_t0.CEL 52.3% 41.54
A143_30_Nugo_031_t2.CEL 48.34% 40.23
A143_29_Nugo_031_t0.CEL 47.92% 38.73
A143_28_Nugo_030_t2.CEL 48.69% 42.74
A143_27_Nugo_030_t0.CEL 50.12% 41.2
A143_26_Nugo_029_t2.CEL 49.65% 45.95
A143_25_Nugo_029_t0.CEL 50.17% 45.4
A143_24_Nugo_024_t2.CEL 50.95% 38.86
A143_23_Nugo_024_t0.CEL 50.24% 37.59
A143_20_Nugo_020_t2.CEL 51.65% 33.05
A143_19_Nugo_020_t0.CEL 50.26% 35.08
A143_18_Nugo_019_t2.CEL 52.31% 35.34
A143_17_Nugo_019_t0.CEL 51.11% 34.72
A143_16_Nugo_018_t2.CEL 49.62% 36.38
A143_15_Nugo_018_t0.CEL 49.63% 36.95
A143_14_Nugo_017_t2.CEL 52.34% 35.84
A143_13_Nugo_017_t0.CEL 51.28% 35.88
A143_12_Nugo_016_t2.CEL 51.21% 41.95
A143_11_Nugo_016_t0.CEL 49.3% 42.11
A143_10_Nugo_014_t2.CEL 52.12% 39.7
A143_09_Nugo_014_t0.CEL 50.05% 38.36
A143_08_Nugo_009_t2.CEL 51.06% 37.11
A143_07_Nugo_009_t0.CEL 50.77% 40.7
A143_02_Nugo_001_t2.CEL 49.81% 48.17
A143_01_Nugo_001_t0.CEL 50.39% 48.02
−3 −2 −1 0 1 2 3

A143_01_Nugo_001_t0.CEL

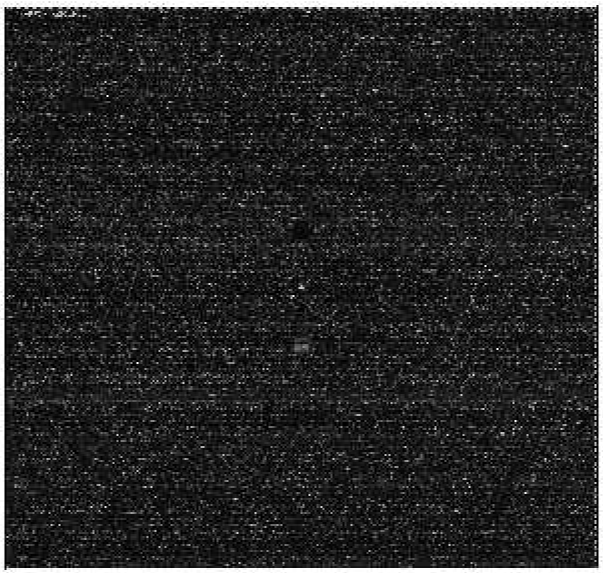

Raw image plot: 1/38 (Thu Mar 12 14:43:13 2009)

A143_02_Nugo_001_t2.CEL

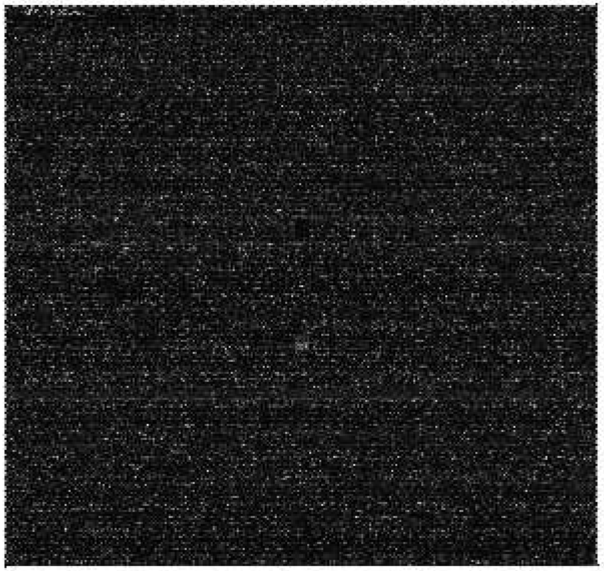

Raw image plot: 2/38 (Thu Mar 12 14:43:18 2009)

A143_07_Nugo_009_t0.CEL

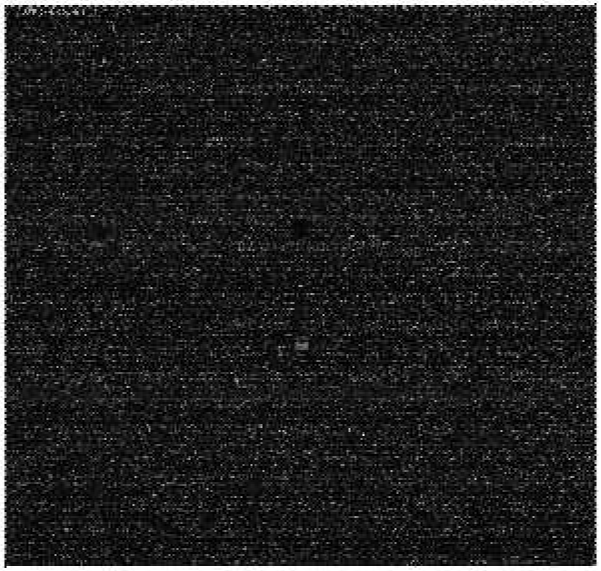

Raw image plot: 3/38 (Thu Mar 12 14:43:22 2009)

A143_08_Nugo_009_t2.CEL

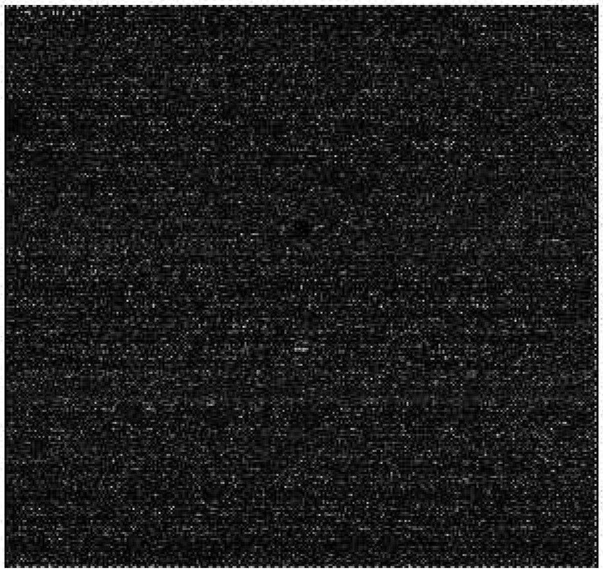

Raw image plot: 4/38 (Thu Mar 12 14:43:27 2009)

A143_09_Nugo_014_t0.CEL

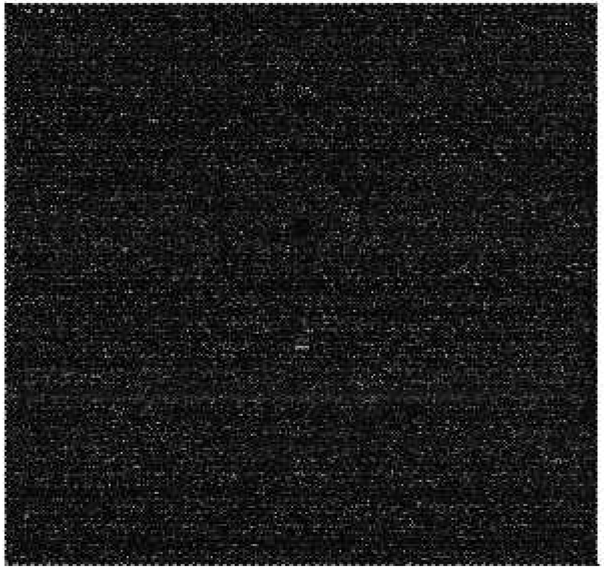

Raw image plot: 5/38 (Thu Mar 12 14:43:31 2009)

A143_10_Nugo_014_t2.CEL

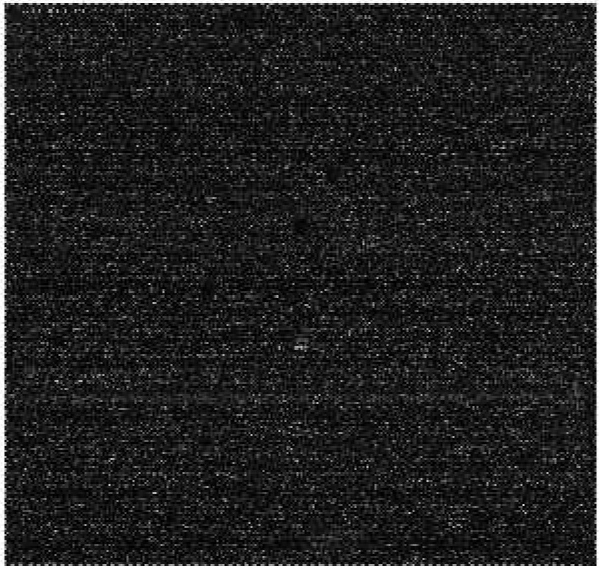

Raw image plot: 6/38 (Thu Mar 12 14:43:35 2009)

A143_11_Nugo_016_t0.CEL

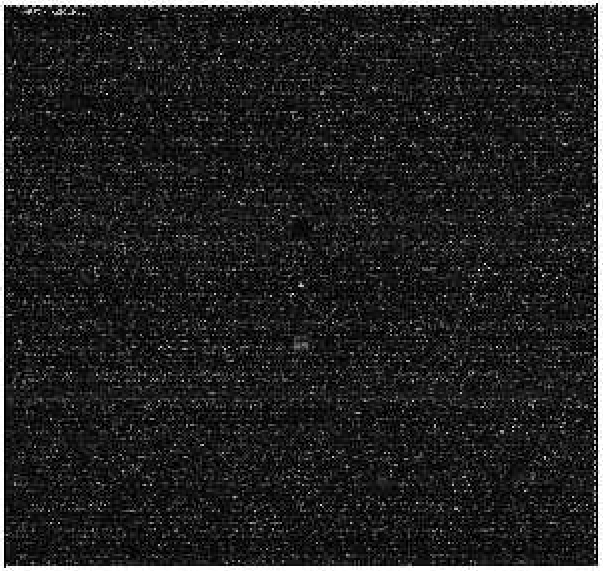

Raw image plot: 7/38 (Thu Mar 12 14:43:41 2009)

A143_12_Nugo_016_t2.CEL

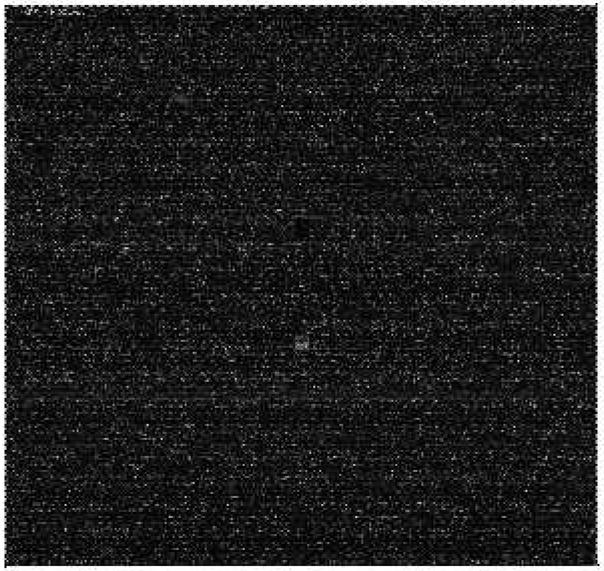

Raw image plot: 8/38 (Thu Mar 12 14:43:45 2009)

A143_13_Nugo_017_t0.CEL

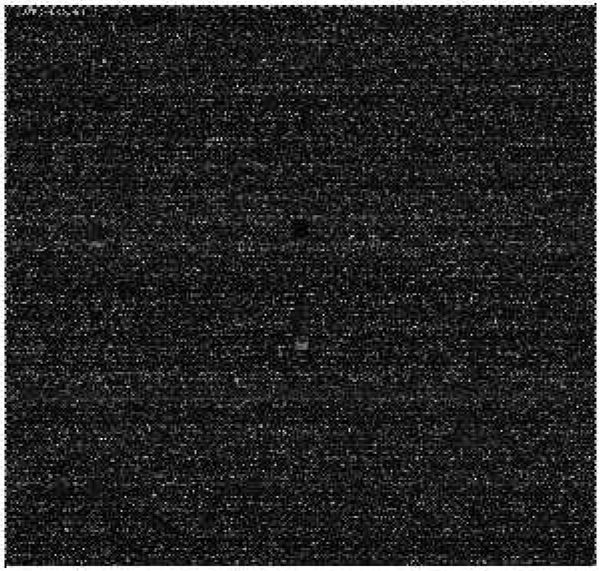

Raw image plot: 9/38 (Thu Mar 12 14:43:49 2009)

A143_14_Nugo_017_t2.CEL

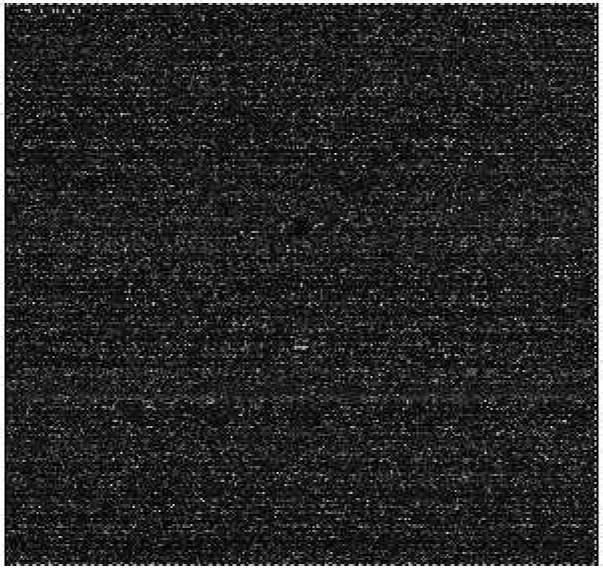

Raw image plot: 10/38 (Thu Mar 12 14:43:54 2009)

A143_15_Nugo_018_t0.CEL

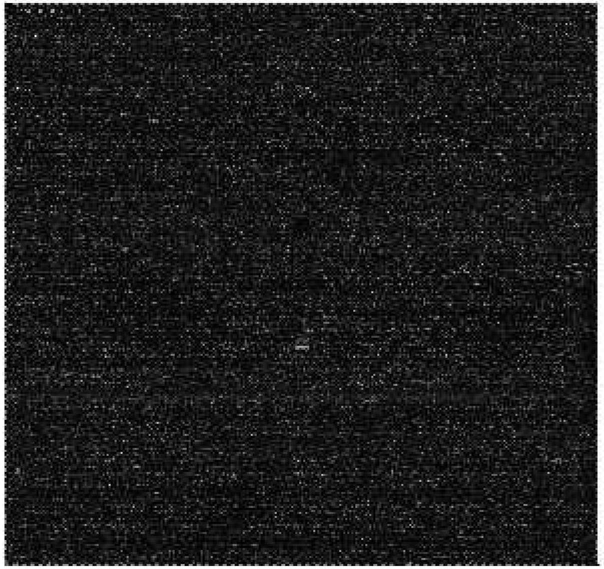

Raw image plot: 11/38 (Thu Mar 12 14:43:58 2009)

A143_16_Nugo_018_t2.CEL

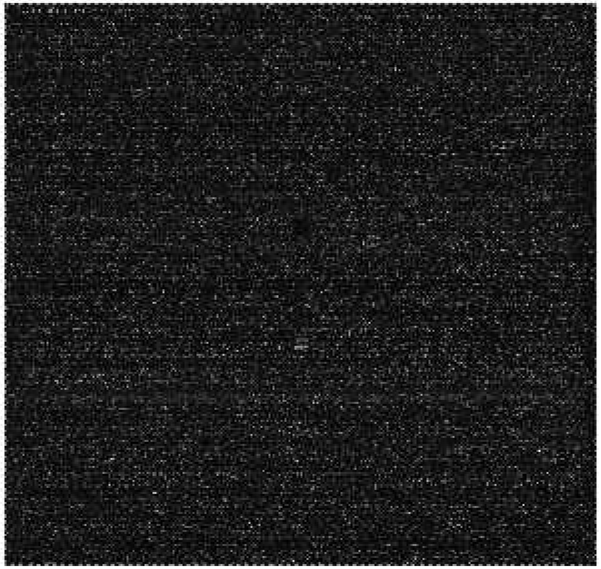

Raw image plot: 12/38 (Thu Mar 12 14:44:03 2009)

A143_17_Nugo_019_t0.CEL

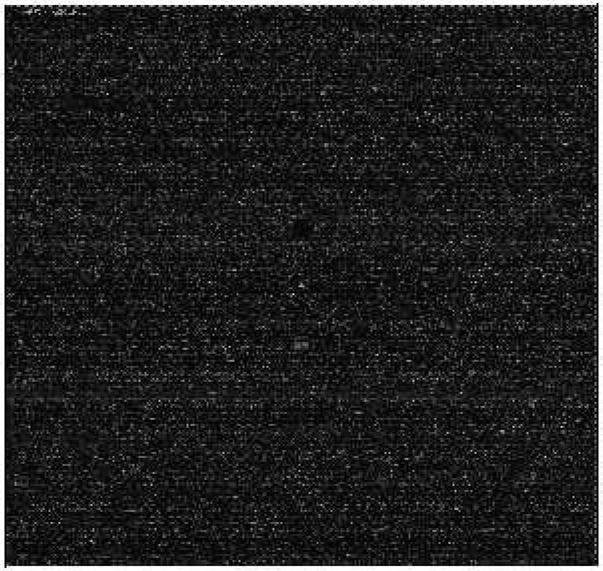

Raw image plot: 13/38 (Thu Mar 12 14:44:08 2009)

A143_18_Nugo_019_t2.CEL

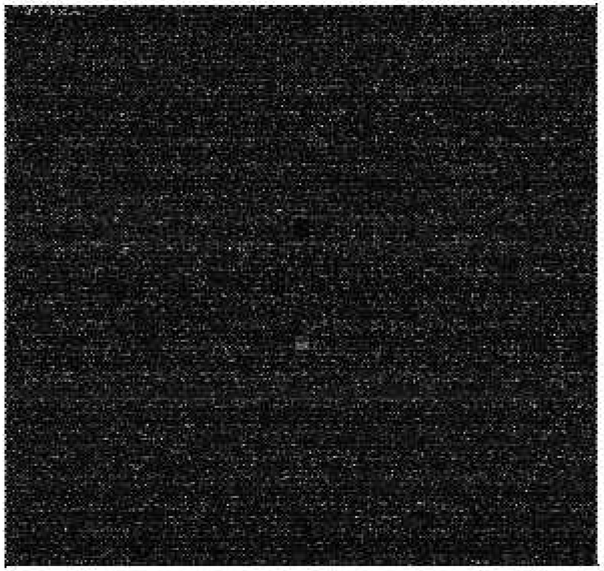

Raw image plot: 14/38 (Thu Mar 12 14:44:12 2009)

A143_19_Nugo_020_t0.CEL

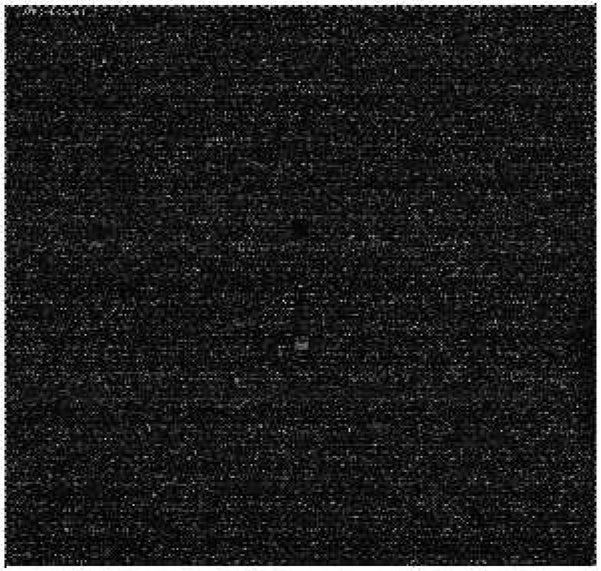

Raw image plot: 15/38 (Thu Mar 12 14:44:17 2009)

A143_20_Nugo_020_t2.CEL

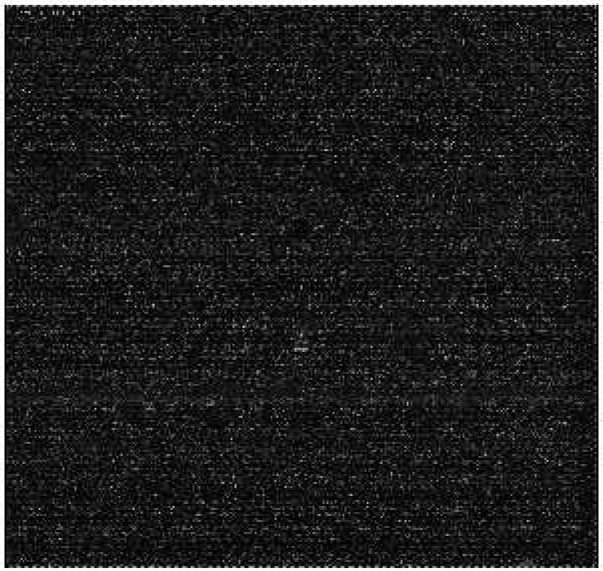

Raw image plot: 16/38 (Thu Mar 12 14:44:21 2009)

A143_23_Nugo_024_t0.CEL

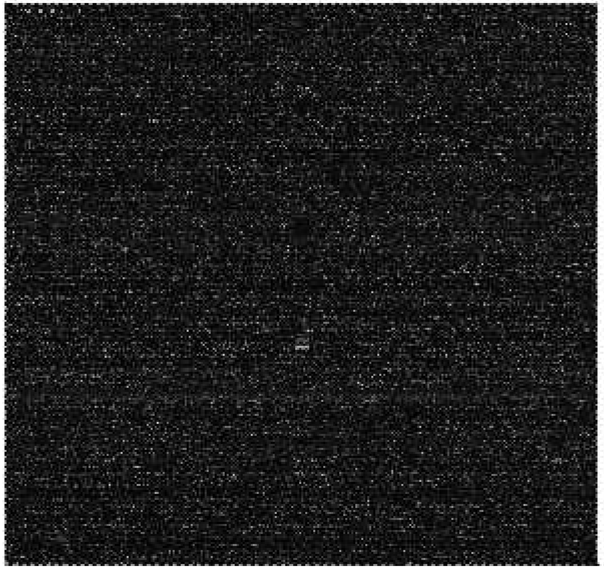

Raw image plot: 17/38 (Thu Mar 12 14:44:25 2009)

A143_24_Nugo_024_t2.CEL

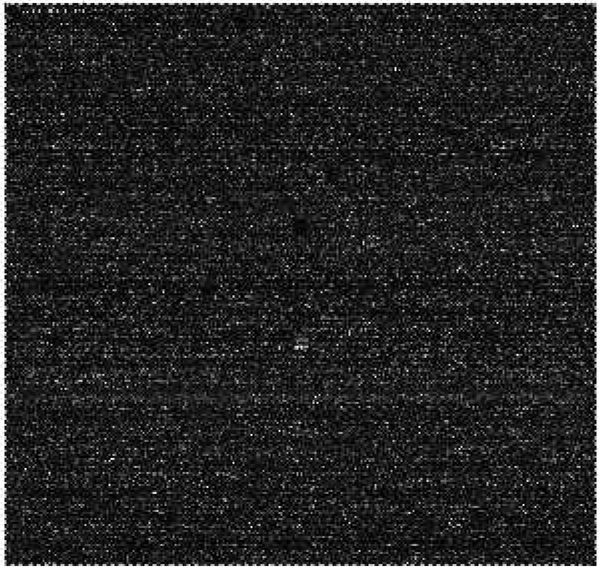

Raw image plot: 18/38 (Thu Mar 12 14:44:30 2009)

A143_25_Nugo_029_t0.CEL

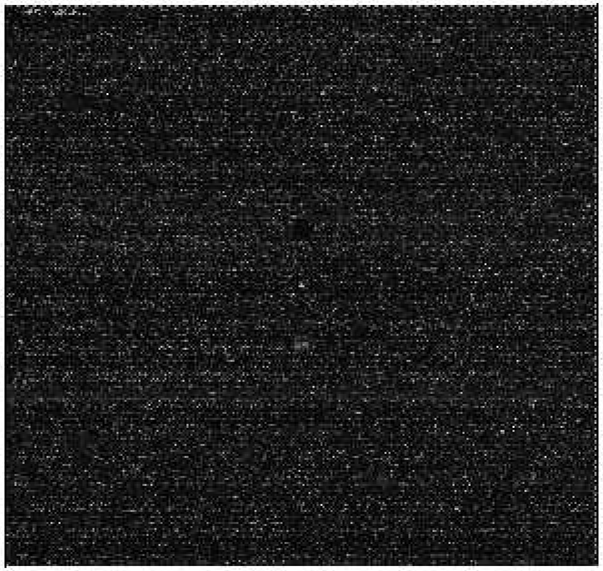

Raw image plot: 19/38 (Thu Mar 12 14:44:35 2009)

A143_26_Nugo_029_t2.CEL

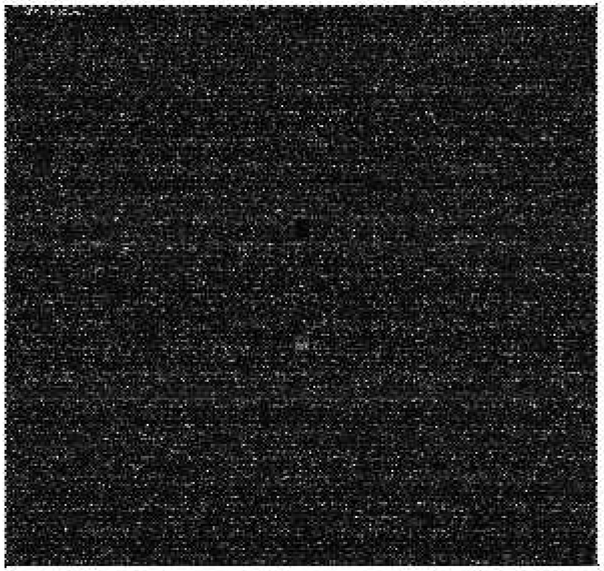

Raw image plot: 20/38 (Thu Mar 12 14:44:40 2009)

A143_27_Nugo_030_t0.CEL

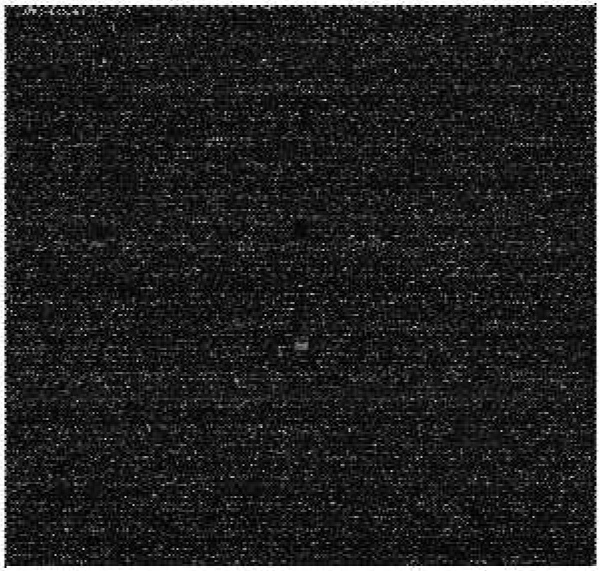

Raw image plot: 21/38 (Thu Mar 12 14:44:44 2009)

A143_28_Nugo_030_t2.CEL

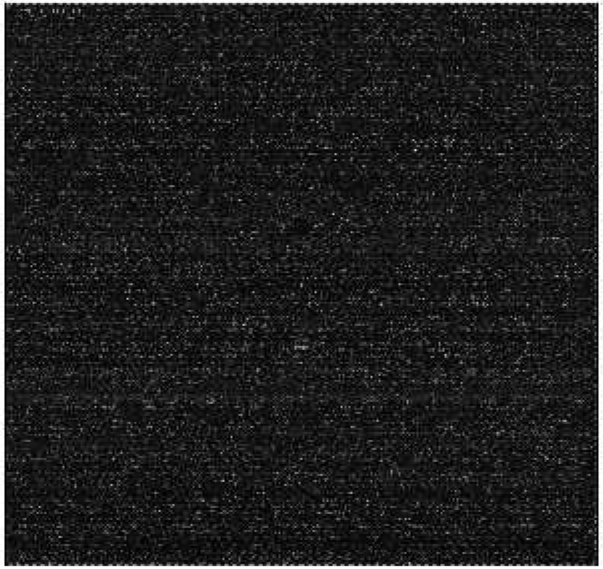

Raw image plot: 22/38 (Thu Mar 12 14:44:48 2009)

A143_29_Nugo_031_t0.CEL

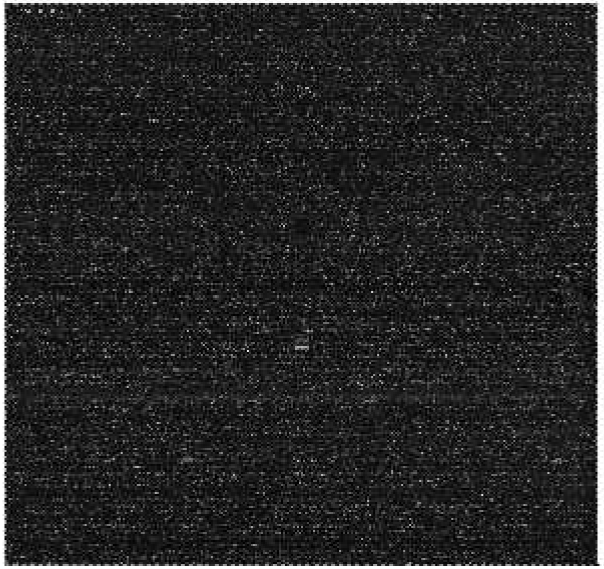

Raw image plot: 23/38 (Thu Mar 12 14:44:52 2009)

A143_30_Nugo_031_t2.CEL

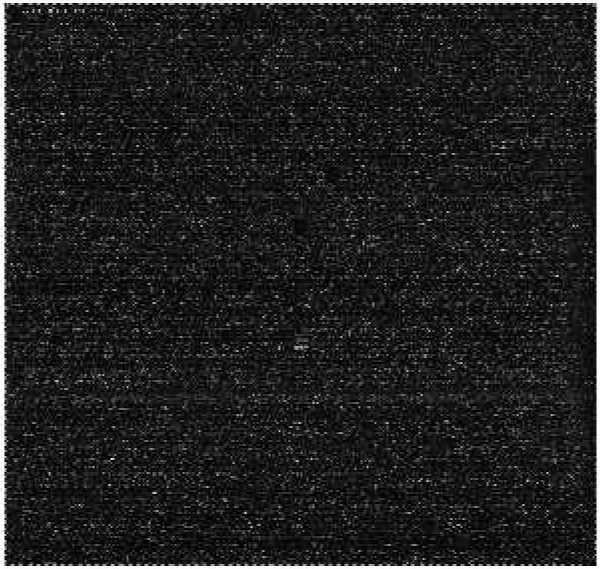

Raw image plot: 24/38 (Thu Mar 12 14:44:57 2009)

A143_31_Nugo_033_t0.CEL

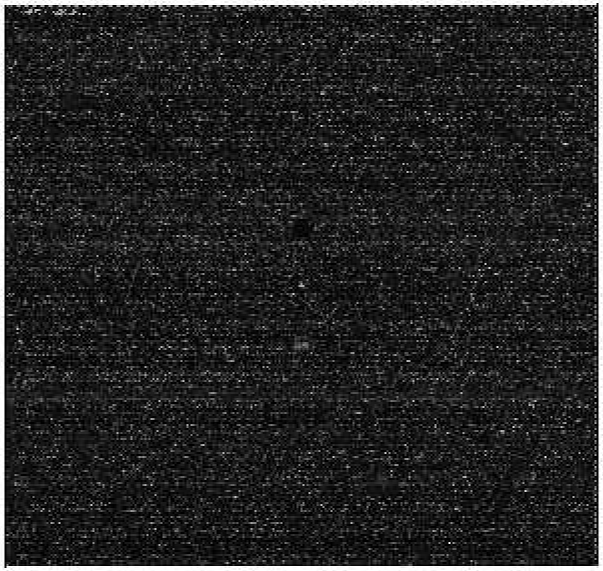

Raw image plot: 25/38 (Thu Mar 12 14:45:02 2009)

A143_32_Nugo_033_t2.CEL

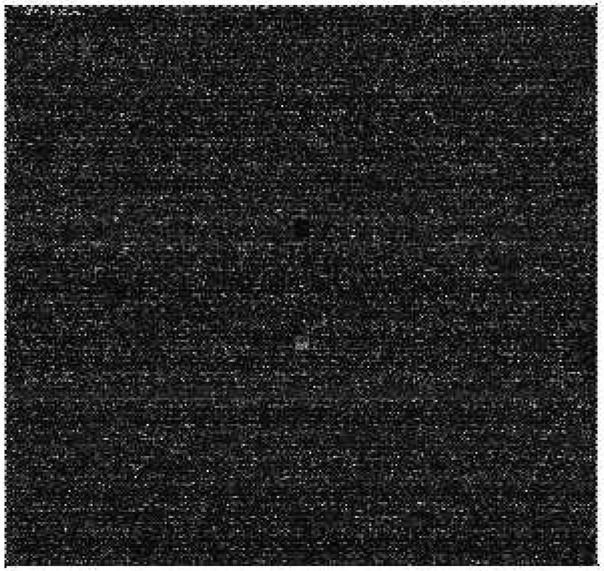

Raw image plot: 26/38 (Thu Mar 12 14:45:07 2009)

A143_33_Nugo_038_t0.CEL

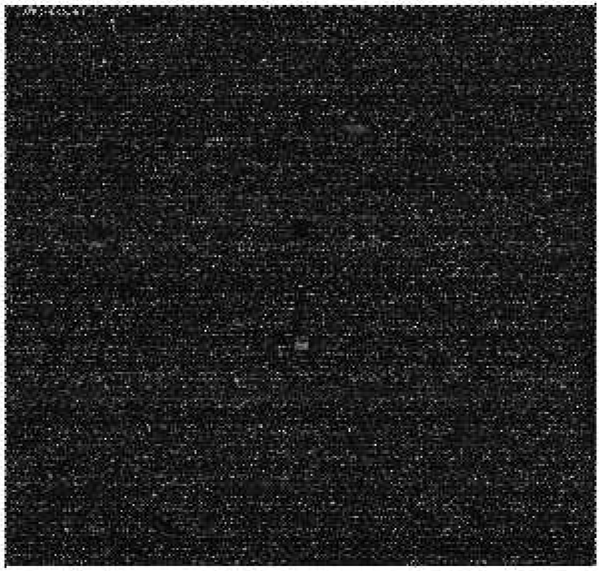

Raw image plot: 27/38 (Thu Mar 12 14:45:11 2009)

A143_34_Nugo_038_t2.CEL

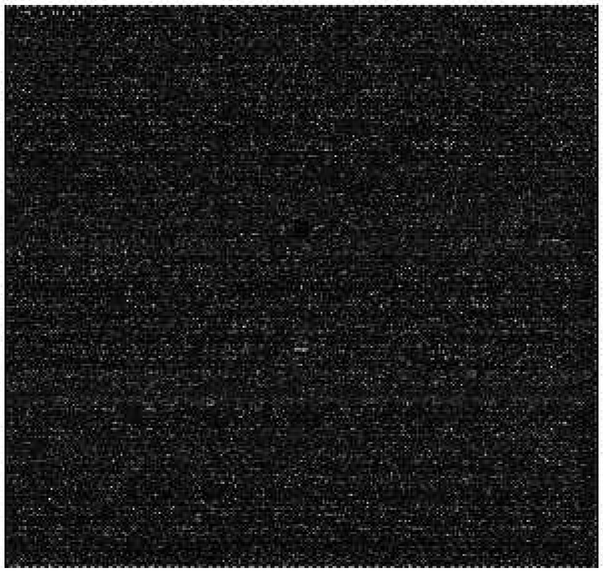

Raw image plot: 28/38 (Thu Mar 12 14:45:15 2009)

A143_35_Nugo_039_t0.CEL

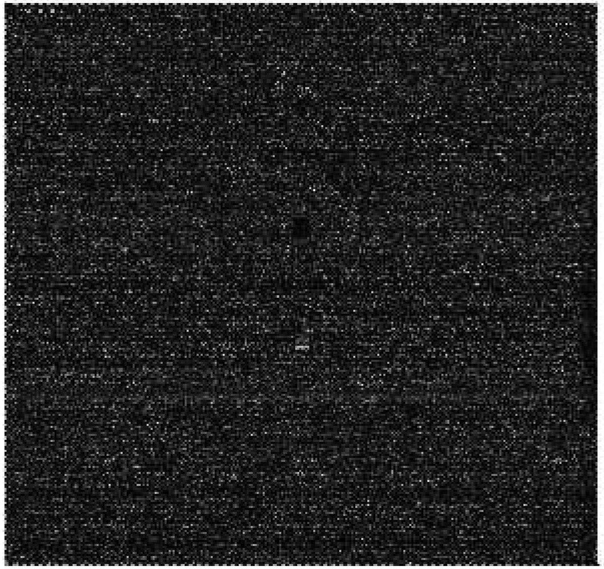

Raw image plot: 29/38 (Thu Mar 12 14:45:19 2009)

A143_36_Nugo_039_t2.CEL

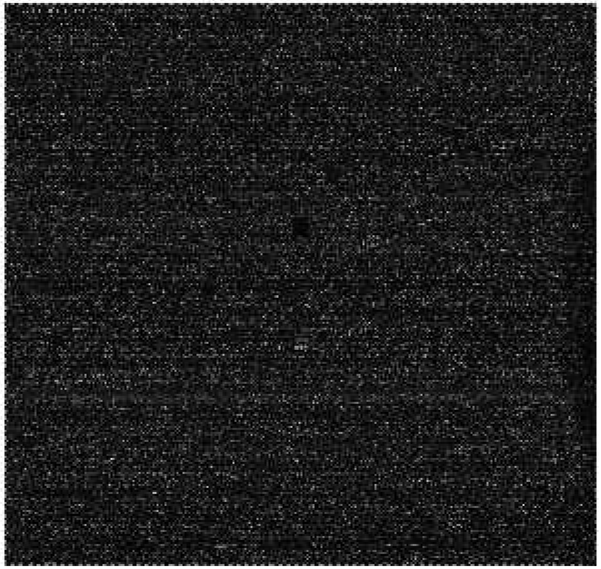

Raw image plot: 30/38 (Thu Mar 12 14:45:24 2009)

A143_37_Nugo_044_t0.CEL

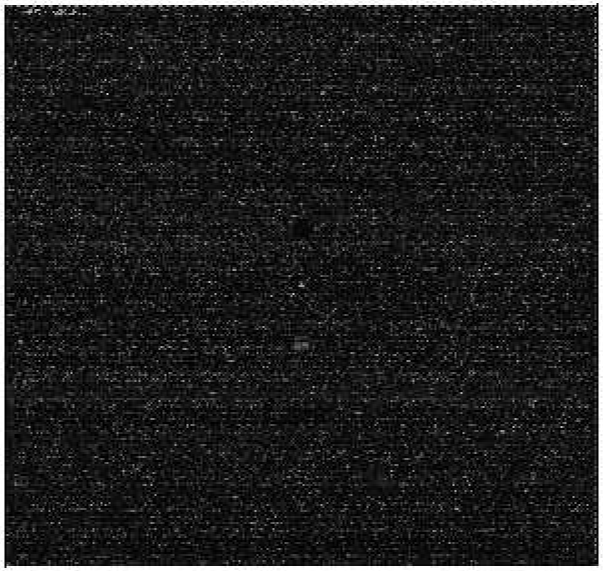

Raw image plot: 31/39 (Thu Mar 12 14:45:29 2009)

A143_38_Nugo_044_t2.CEL

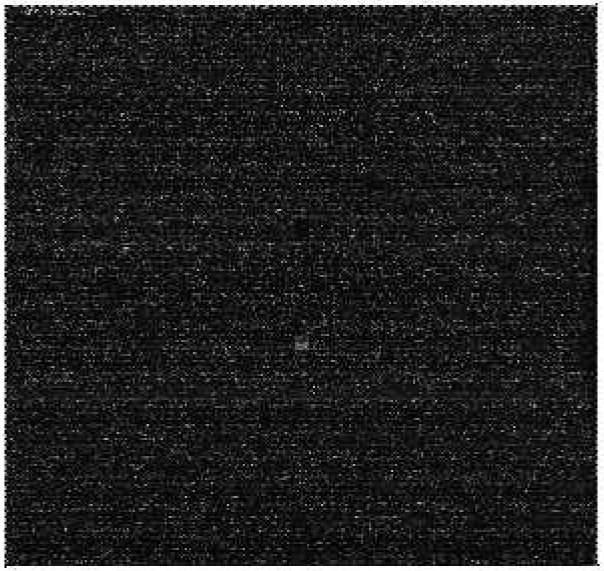

Raw image plot: 32/39 (Thu Mar 12 14:45:33 2009)

A143_39_Nugo_045_t0.CEL

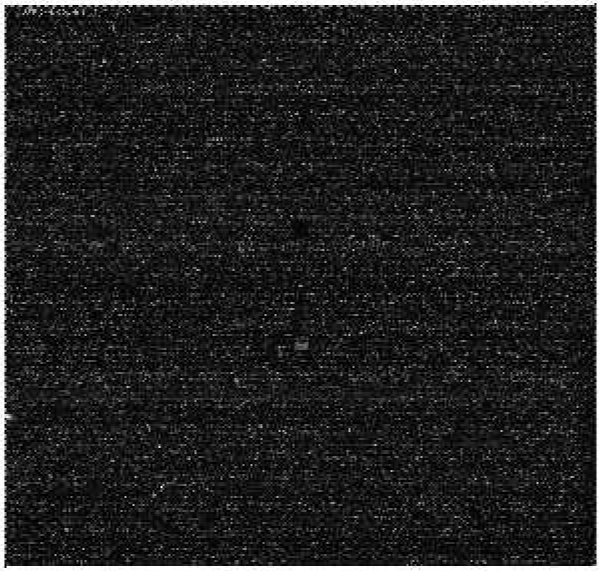

Raw image plot: 33/39 (Thu Mar 12 14:45:38 2009)

A143_40_Nugo_045_t2.CEL

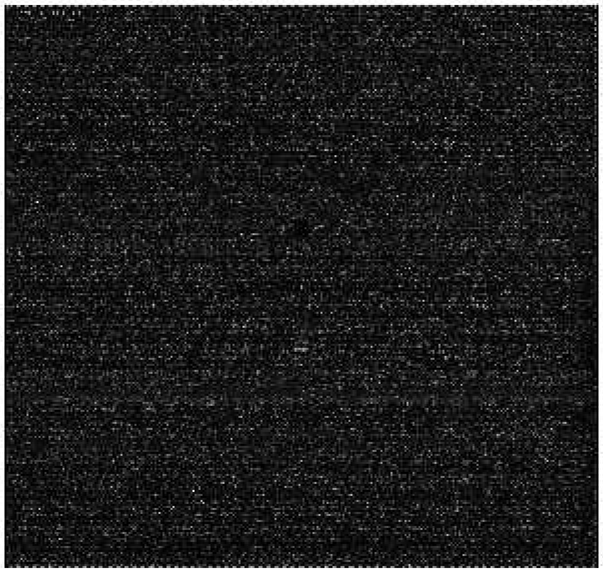

Raw image plot: 34/39 (Thu Mar 12 14:45:42 2009)

A143_41_Nugo_048_t0.CEL

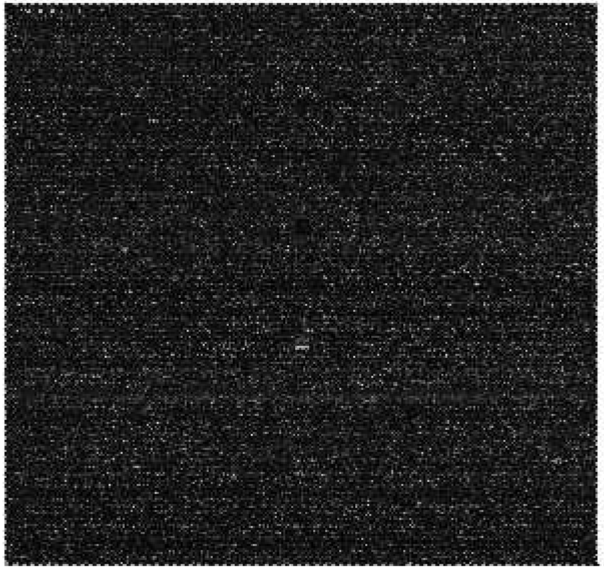

Raw image plot: 35/39 (Thu Mar 12 14:45:46 2009)

A143_42_Nugo_048_t2.CEL

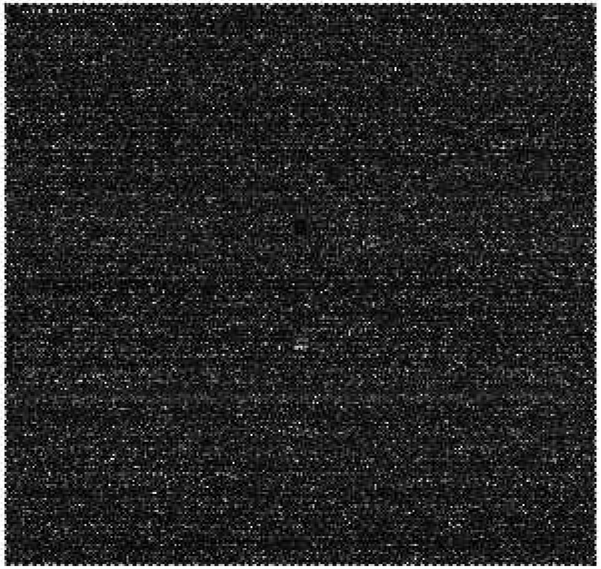

Raw image plot: 36/39 (Thu Mar 12 14:45:50 2009)

A143_43A_Nugo_053_t0.CEL

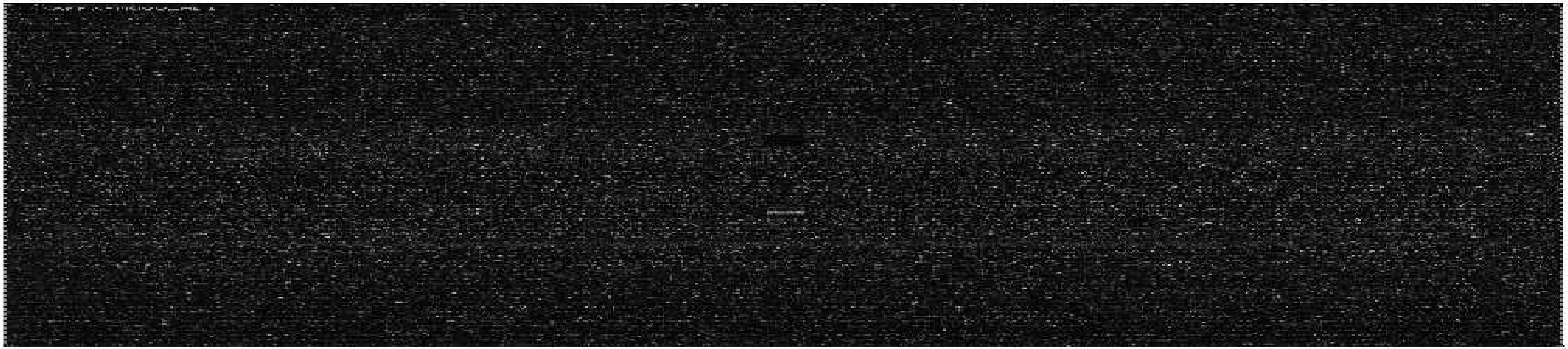

Raw image plot: 37/38 (Thu Mar 12 14:45:55 2009)

A143_44A_Nugo_053_t2.CEL

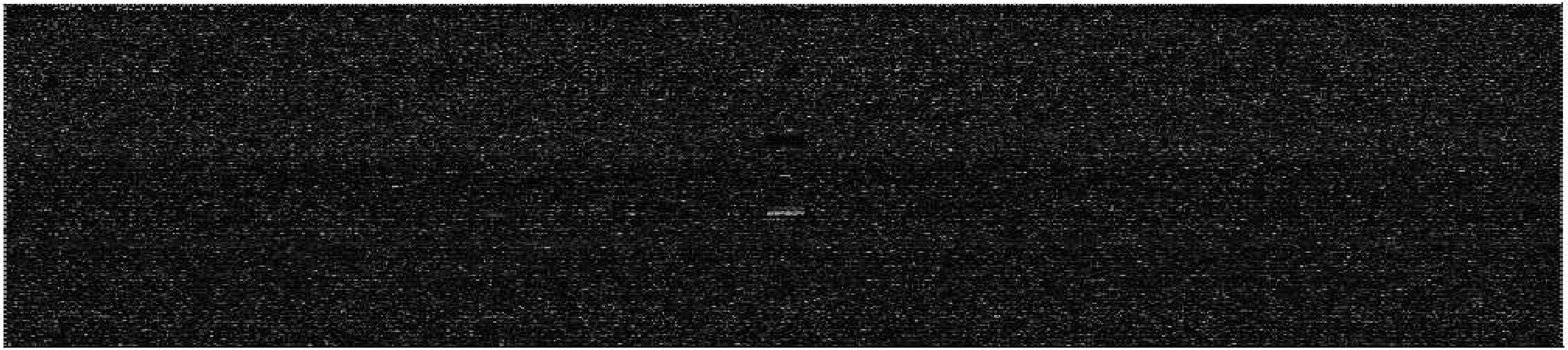

Raw image plot: 38/38 (Thu Mar 12 14:46:00 2009)

A143_01_Nugo_001_t0.CEL

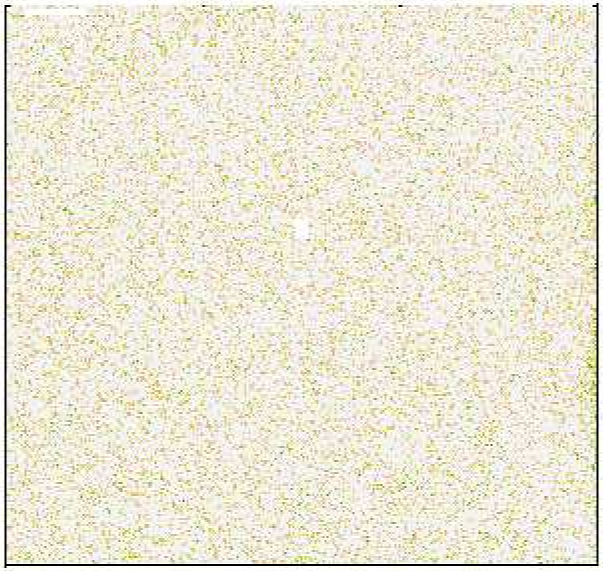

fitPLM weight image plot: 1/38 (Thu Mar 12 14:55:04 2009)

A143_02_Nugo_001_t2.CEL

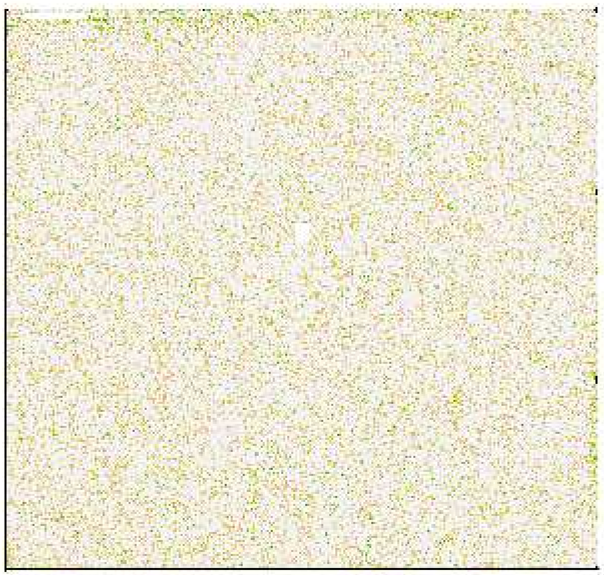

fitPLM weight image plot: 2/38 (Thu Mar 12 14:55:06 2009)

A143_07_Nugo_009_t0.CEL

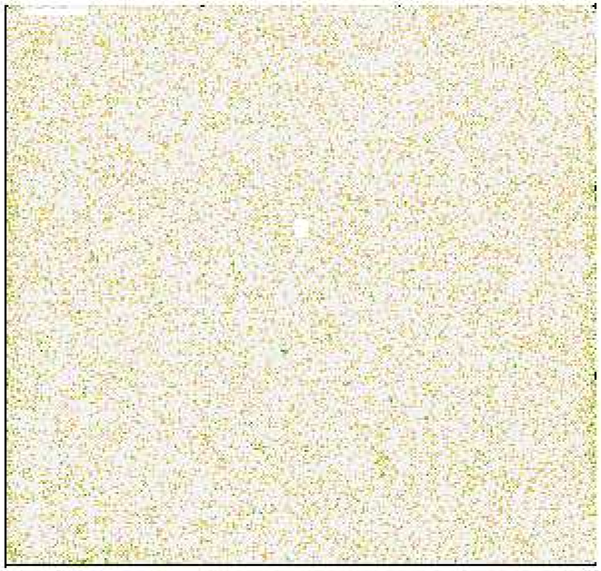

fitPLM weight image plot: 3/38 (Thu Mar 12 14:55:09 2009)

A143_08_Nugo_009_t2.CEL

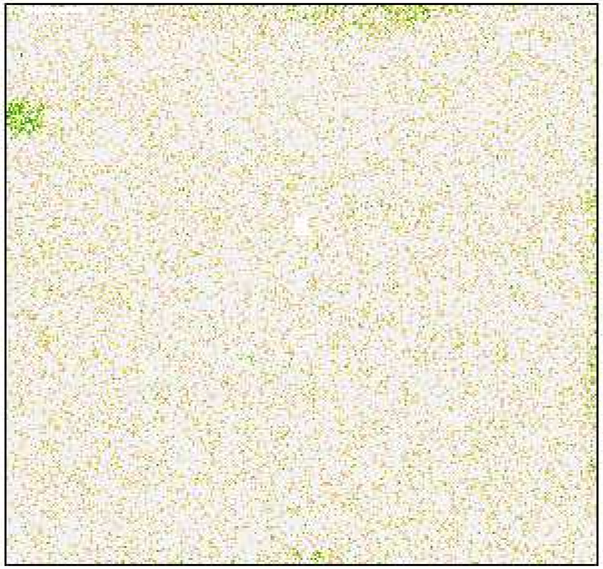

fitPLM weight image plot: 4/38 (Thu Mar 12 14:55:12 2009)

A143_09_Nugo_014_t0.CEL

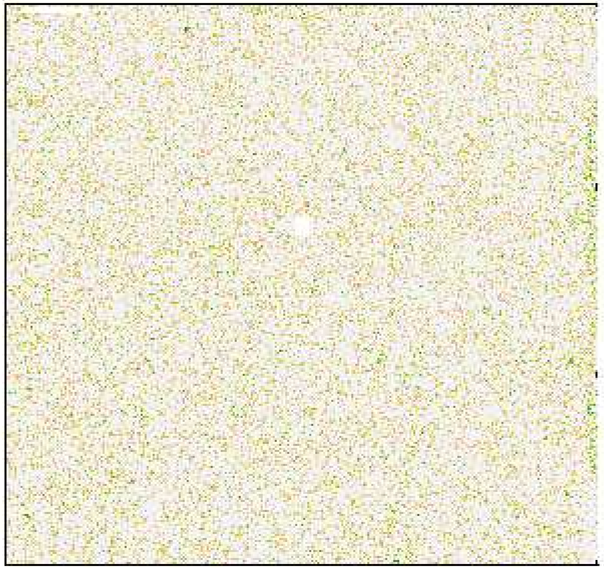

fitPLM weight image plot: 5/38 (Thu Mar 12 14:55:15 2009)

A143_10_Nugo_014_t2.CEL

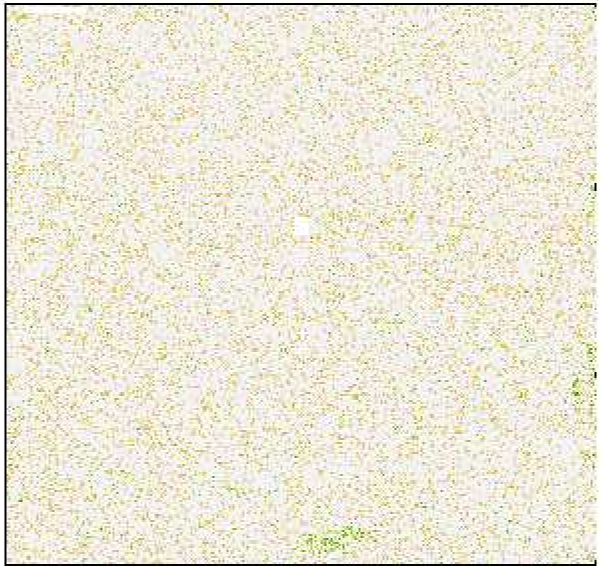

fitPLM weight image plot: 6/38 (Thu Mar 12 14:55:18 2009)

A143_11_Nugo_016_t0.CEL

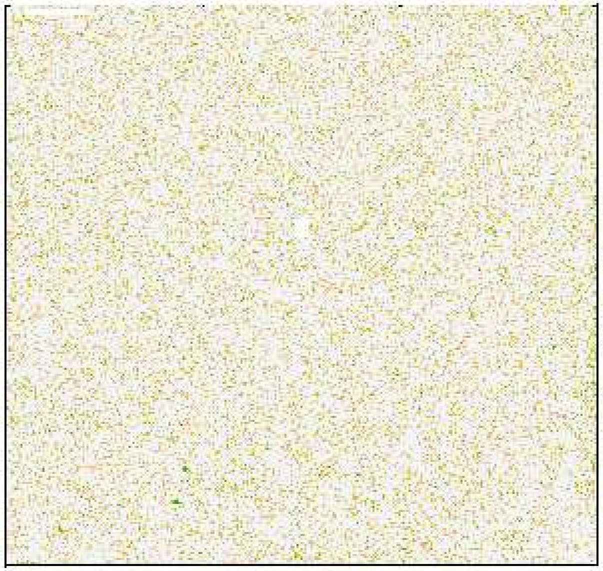

fitPLM weight image plot: 7/38 (Thu Mar 12 14:55:24 2009)

A143_12_Nugo_016_t2.CEL

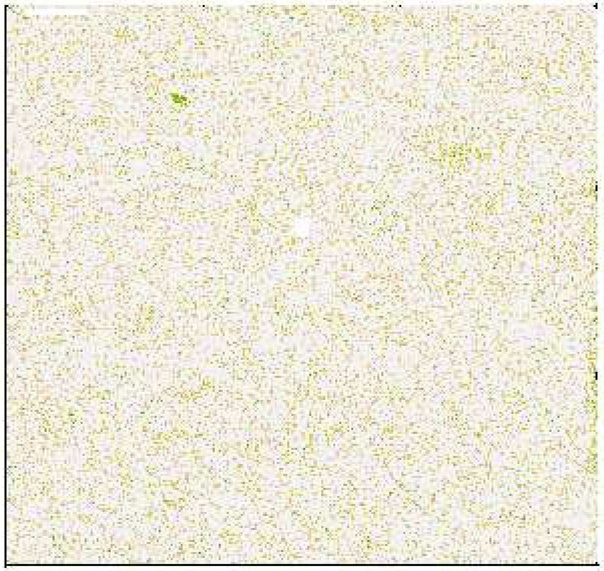

fitPLM weight image plot: 8/38 (Thu Mar 12 14:55:26 2009)

A143_13_Nugo_017_t0.CEL

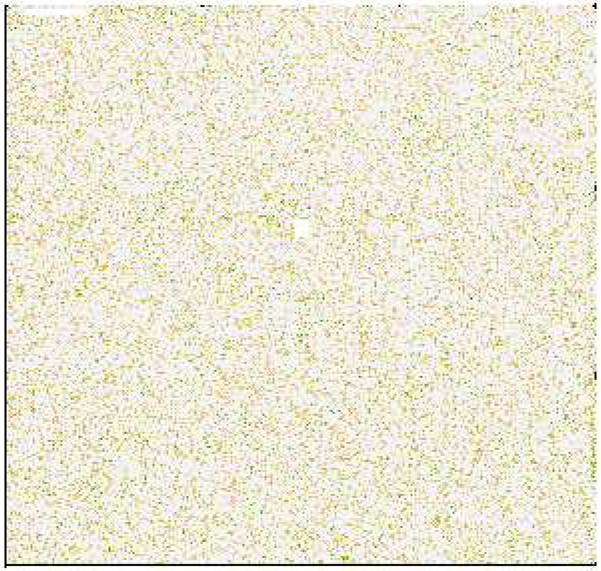

fitPLM weight image plot: 9/38 (Thu Mar 12 14:55:29 2009)

A143_14_Nugo_017_t2.CEL

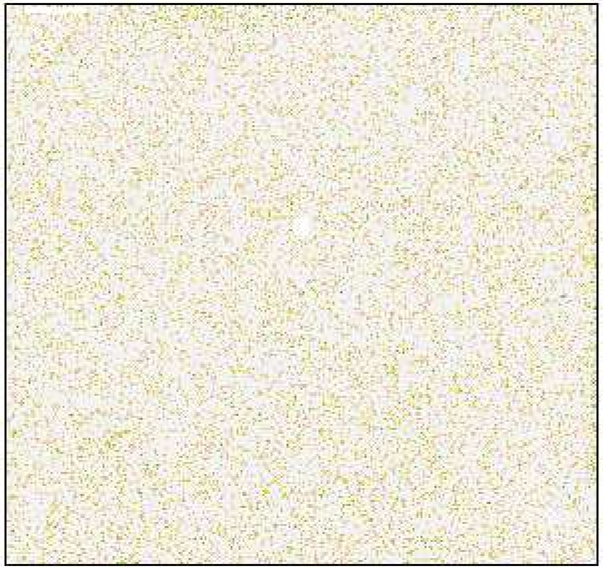

fitPLM weight image plot: 10/38 (Thu Mar 12 14:55:32 2009)

A143_15_Nugo_018_t0.CEL

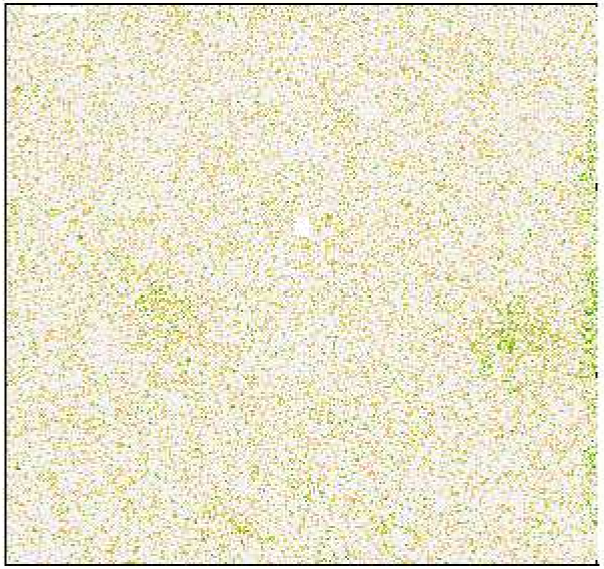

fitPLM weight image plot: 11/38 (Thu Mar 12 14:55:35 2009)

A143_16_Nugo_018_t2.CEL

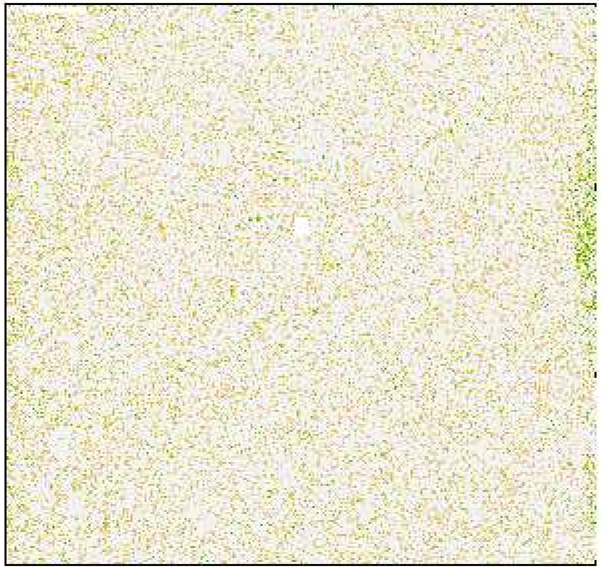

fitPLM weight image plot: 12/38 (Thu Mar 12 14:55:39 2009)

A143_17_Nugo_019_t0.CEL

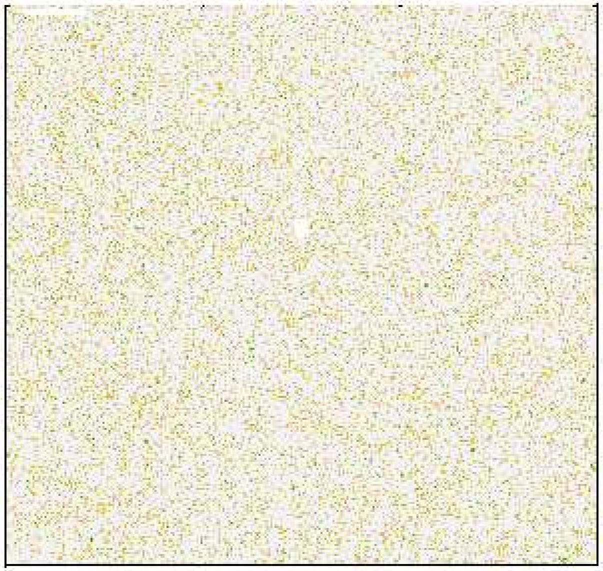

fitPLM weight image plot: 13/38 (Thu Mar 12 14:55:44 2009)

A143_18_Nugo_019_t2.CEL

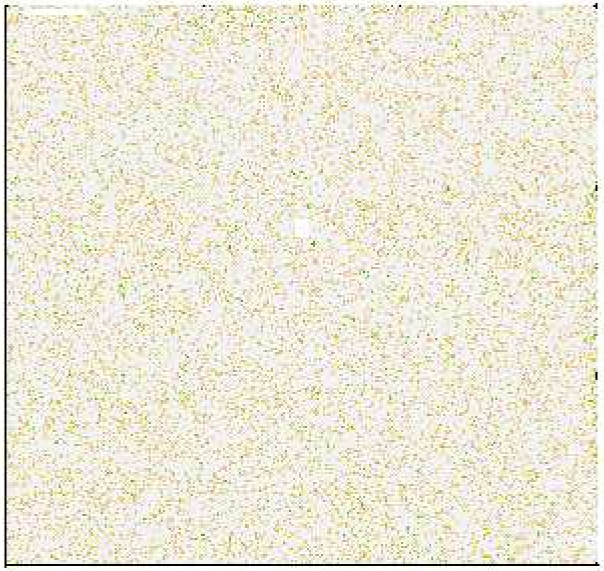

fitPLM weight image plot: 14/38 (Thu Mar 12 14:55:47 2009)

A143_19_Nugo_020_t0.CEL

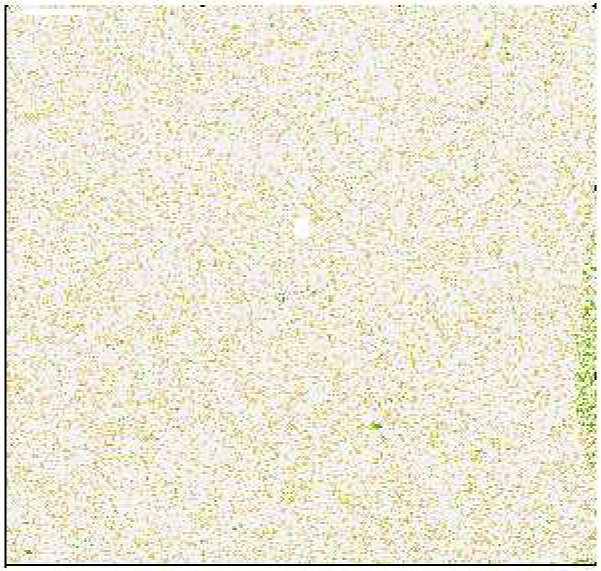

fitPLM weight image plot: 15/38 (Thu Mar 12 14:55:50 2009)

A143_20_Nugo_020_t2.CEL

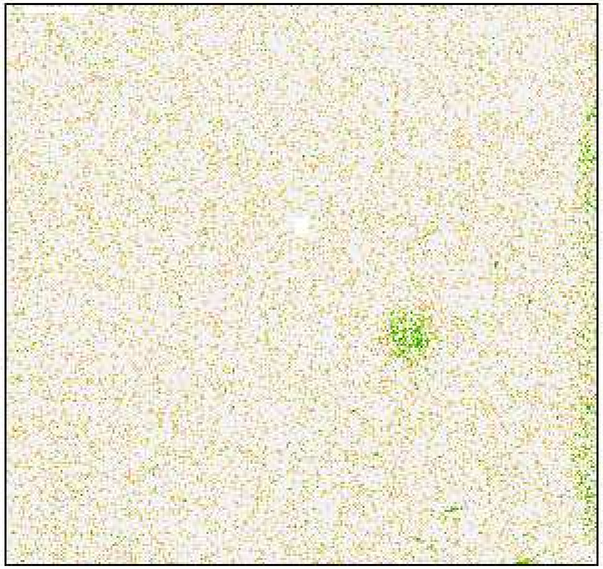

fitPLM weight image plot: 16/38 (Thu Mar 12 14:55:53 2009)

A143_23_Nugo_024_t0.CEL

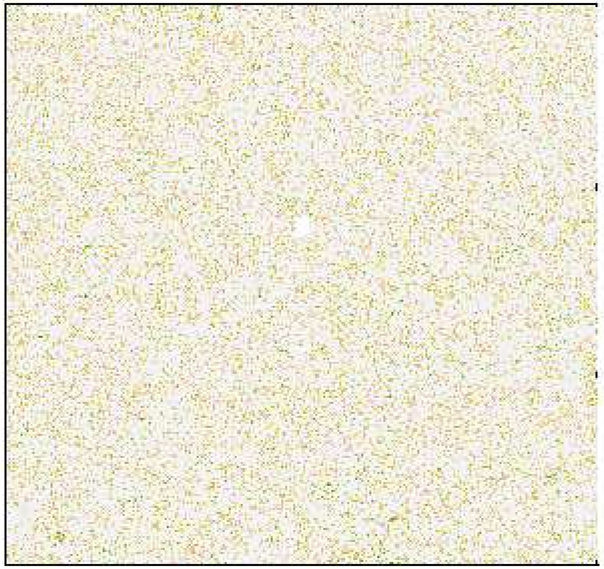

fitPLM weight image plot: 17/38 (Thu Mar 12 14:55:56 2009)

A143_24_Nugo_024_t2.CEL

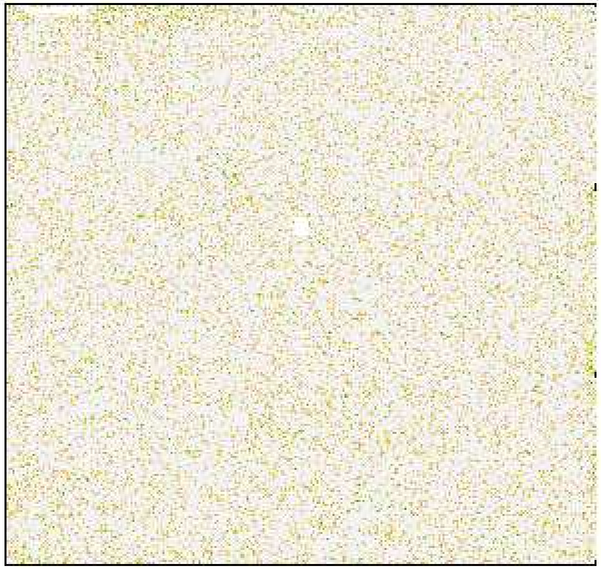

fitPLM weight image plot: 18/38 (Thu Mar 12 14:55:59 2009)

A143_25_Nugo_029_t0.CEL

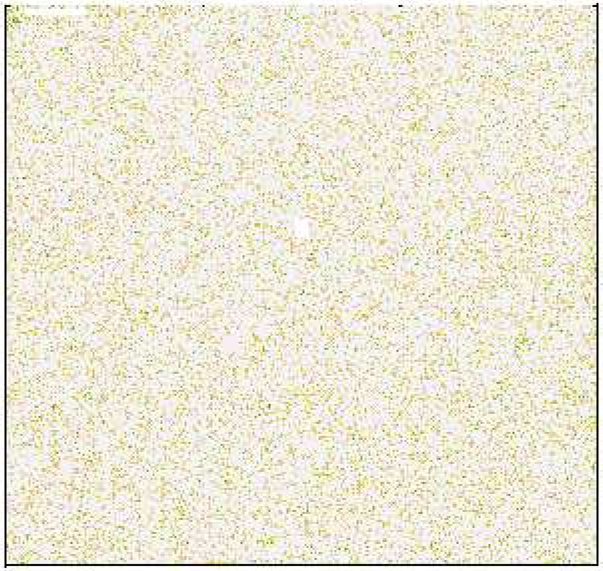

fitPLM weight image plot: 19/38 (Thu Mar 12 14:56:04 2009)

A143_26_Nugo_029_t2.CEL

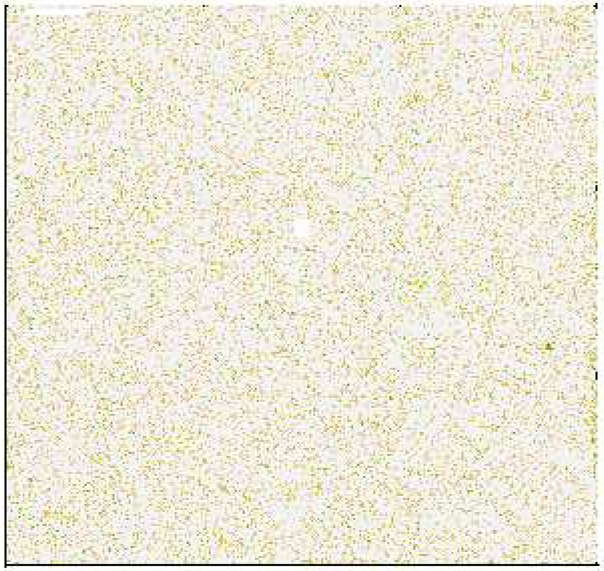

fitPLM weight image plot: 20/38 (Thu Mar 12 14:56:07 2009)

A143_27_Nugo_030_t0.CEL

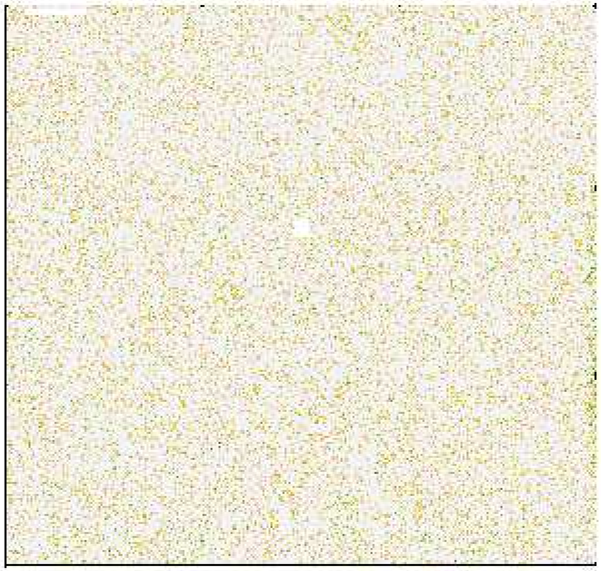

fitPLM weight image plot: 21/38 (Thu Mar 12 14:56:09 2009)

A143_28_Nugo_030_t2.CEL

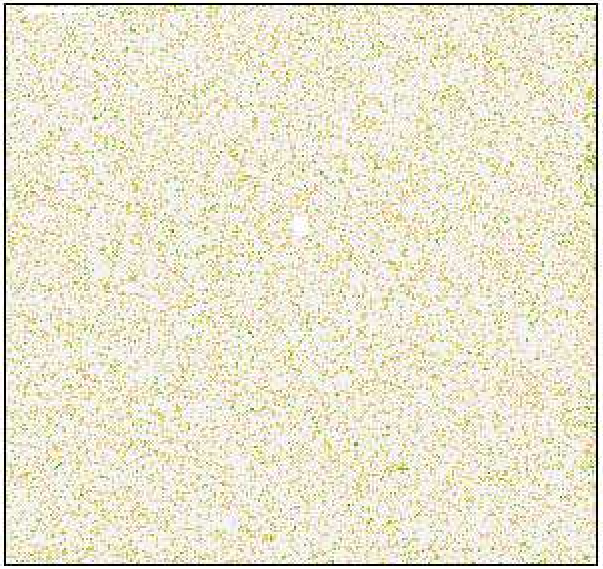

fitPLM weight image plot: 22/38 (Thu Mar 12 14:56:13 2009)

A143_29_Nugo_031_t0.CEL

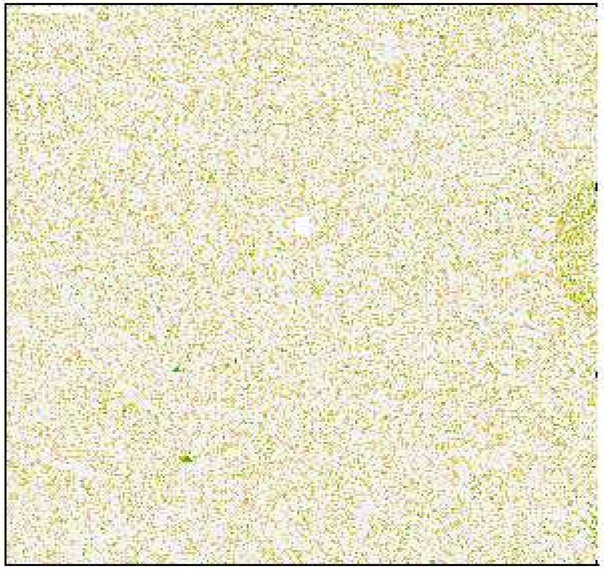

fitPLM weight image plot: 23/38 (Thu Mar 12 14:56:16 2009)

A143_30_Nugo_031_t2.CEL

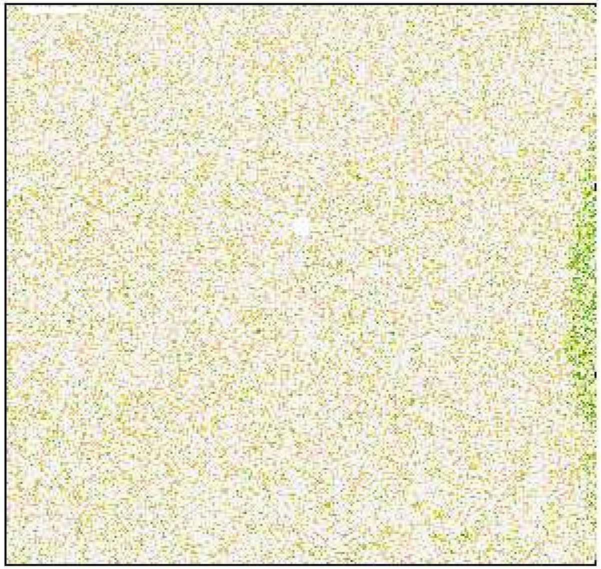

fitPLM weight image plot: 24/38 (Thu Mar 12 14:56:19 2009)

A143_31_Nugo_033_t0.CEL

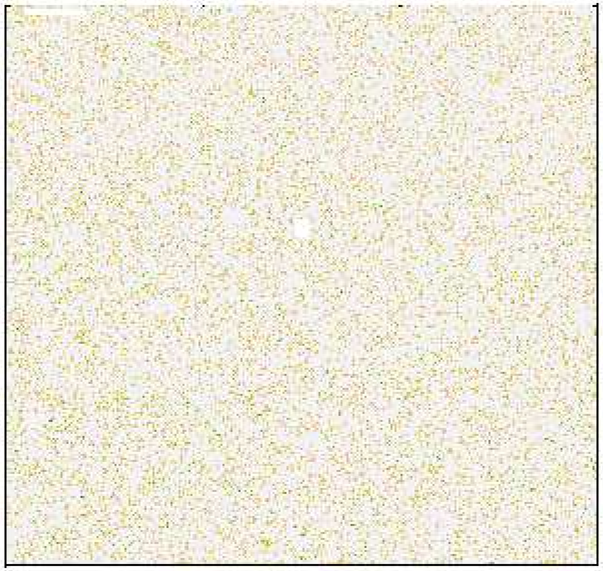

fitPLM weight image plot: 25/38 (Thu Mar 12 14:56:24 2009)

A143_32_Nugo_033_t2.CEL

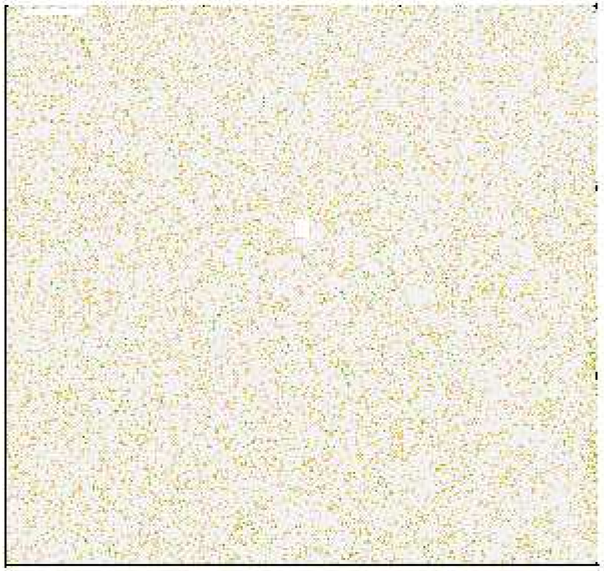

fitPLM weight image plot: 26/38 (Thu Mar 12 14:56:27 2009)

A143_33_Nugo_038_t0.CEL

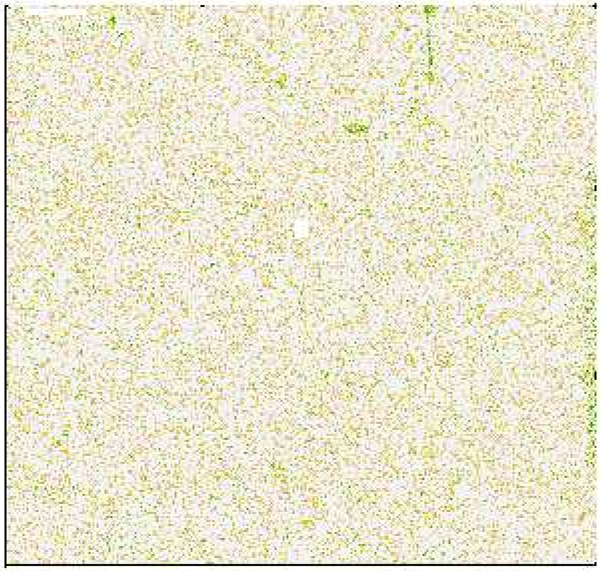

fitPLM weight image plot: 27/38 (Thu Mar 12 14:56:30 2009)

A143_34_Nugo_038_t2.CEL

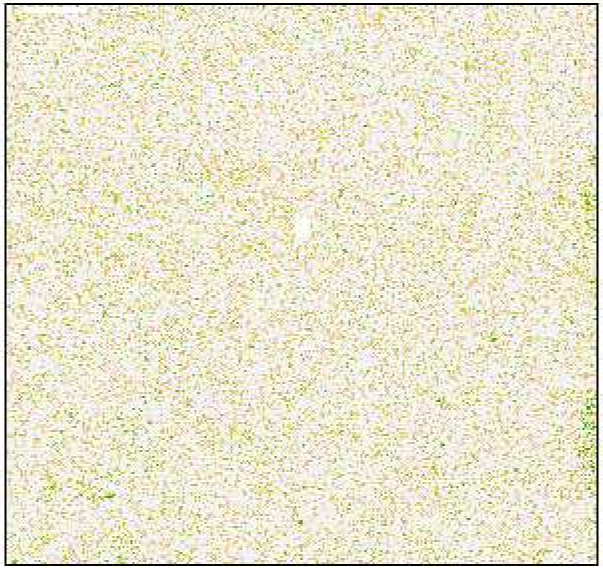

fitPLM weight image plot: 28/38 (Thu Mar 12 14:56:33 2009)

A143_35_Nugo_039_t0.CEL

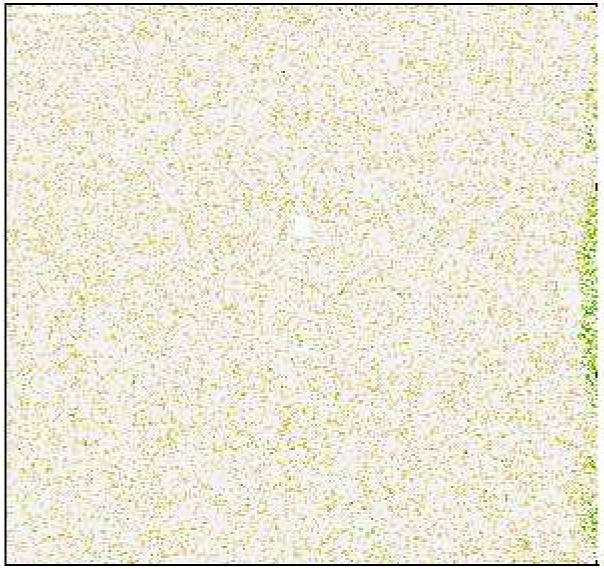

fitPLM weight image plot: 29/38 (Thu Mar 12 14:56:36 2009)

A143_36_Nugo_039_t2.CEL

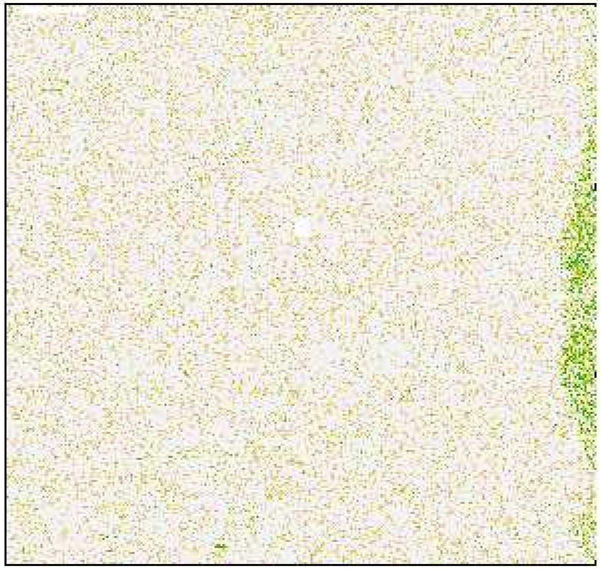

fitPLM weight image plot: 30/38 (Thu Mar 12 14:56:39 2009)

A143_37_Nugo_044_t0.CEL

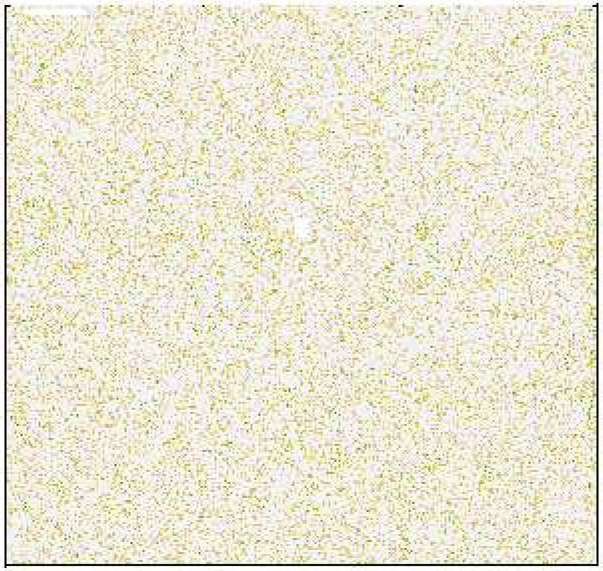

fitPLM weight image plot: 31/38 (Thu Mar 12 14:56:44 2009)

A143_38_Nugo_044_t2.CEL

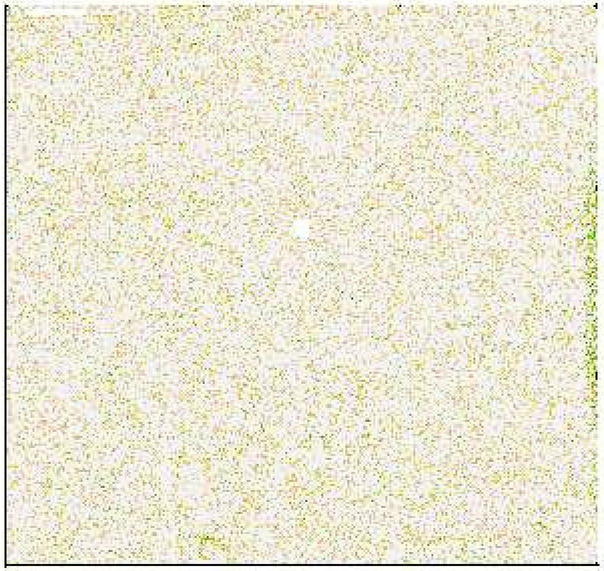

fitPLM weight image plot: 32/38 (Thu Mar 12 14:56:47 2009)

A143_39_Nugo_045_t0.CEL

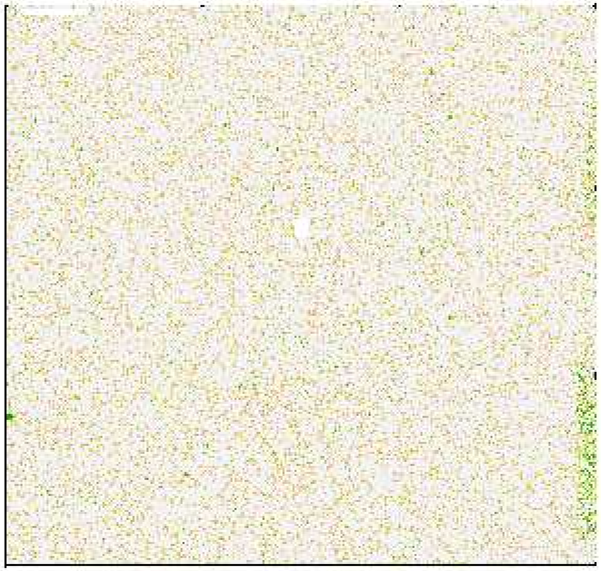

fitPLM weight image plot: 33/38 (Thu Mar 12 14:56:50 2009)

A143_40_Nugo_045_t2.CEL

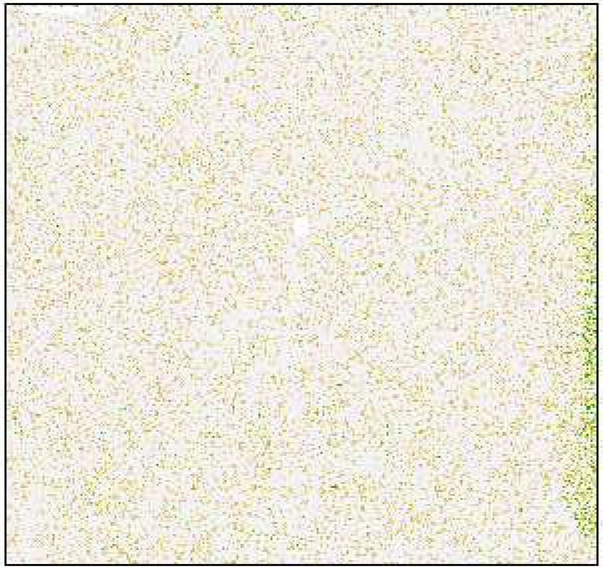

fitPLM weight image plot: 34/38 (Thu Mar 12 14:56:53 2009)

A143_41_Nugo_048_t0.CEL

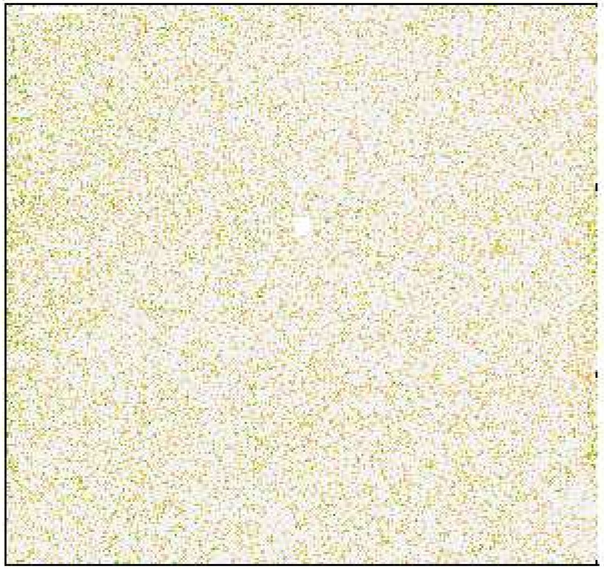

fitPLM weight image plot: 35/38 (Thu Mar 12 14:56:56 2009)

A143_42_Nugo_048_t2.CEL

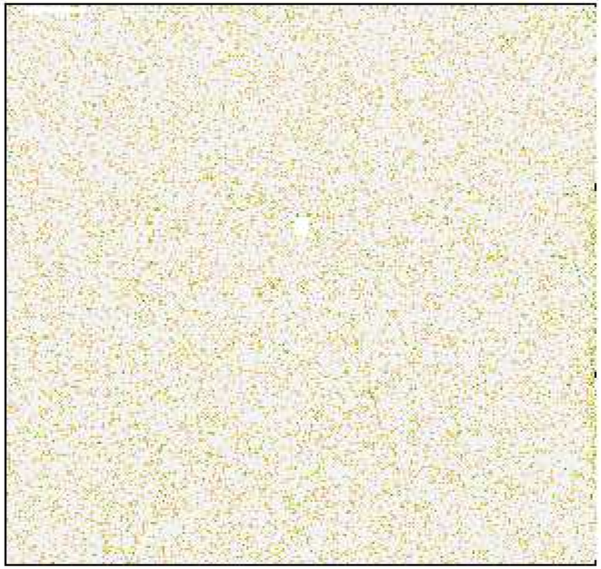

fitPLM weight image plot: 36/38 (Thu Mar 12 14:56:59 2009)

A143_43A_Nugo_053_t0.CEL

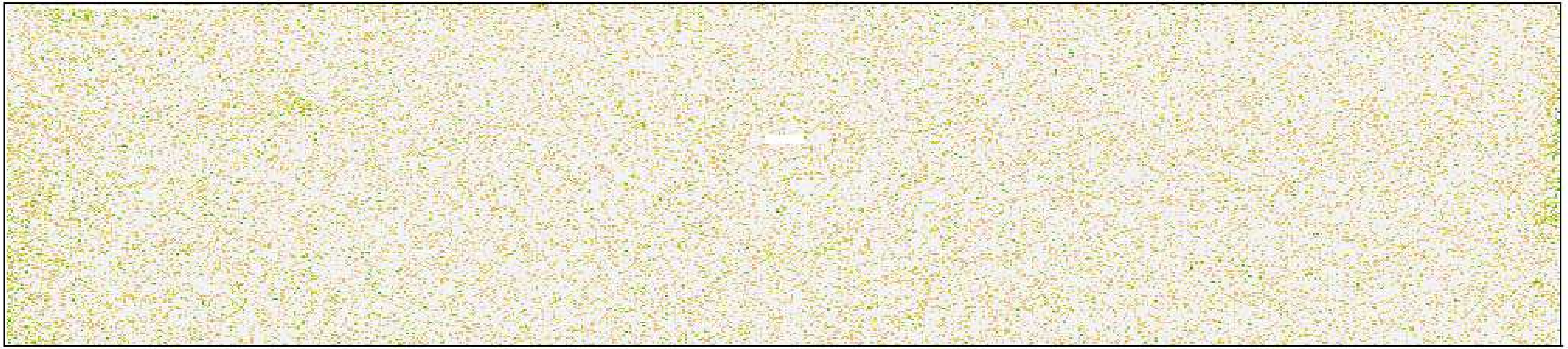

fitPLM weight image plot: 37/38 (Thu Mar 12 14:57:03 2009)

A143_44A_Nugo_053_t2.CEL

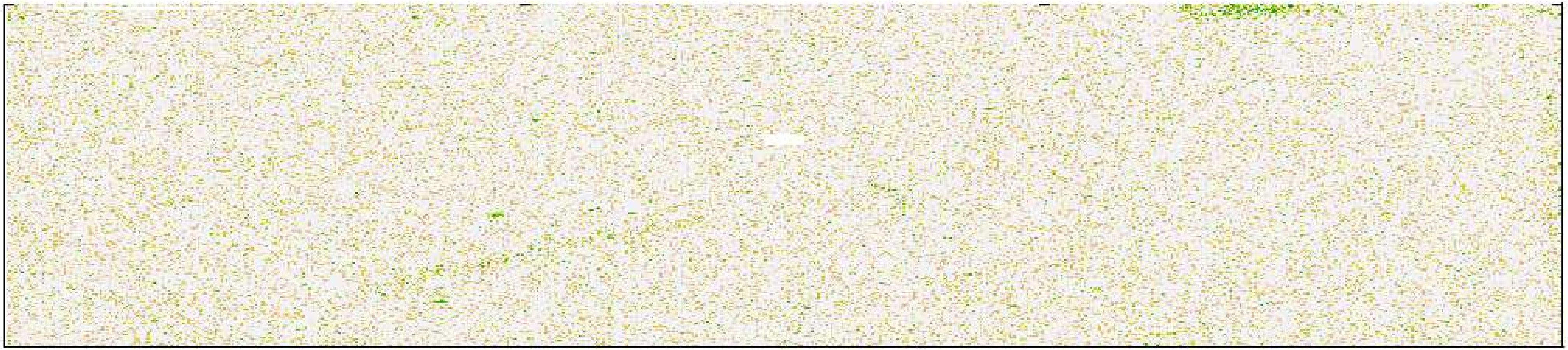

fitPLM weight image plot: 38/38 (Thu Mar 12 14:57:06 2009)

A143_01_Nugo_001_t0.CEL

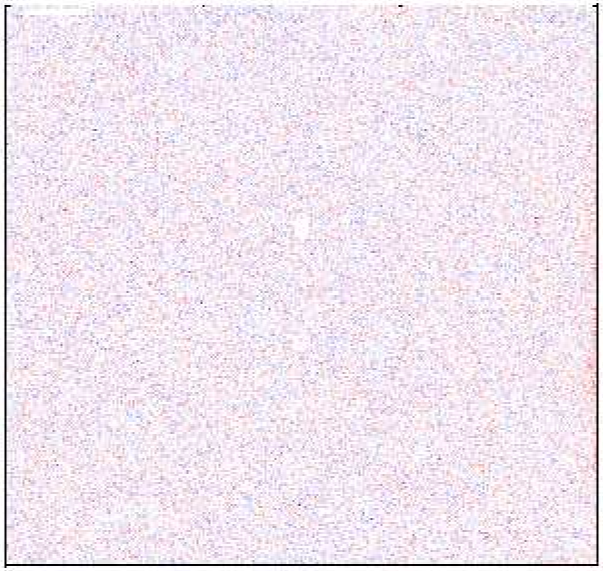

fitPLM residuals image plot: 1/38 (Thu Mar 12 14:57:12 2009)

A143_02_Nugo_001_t2.CEL

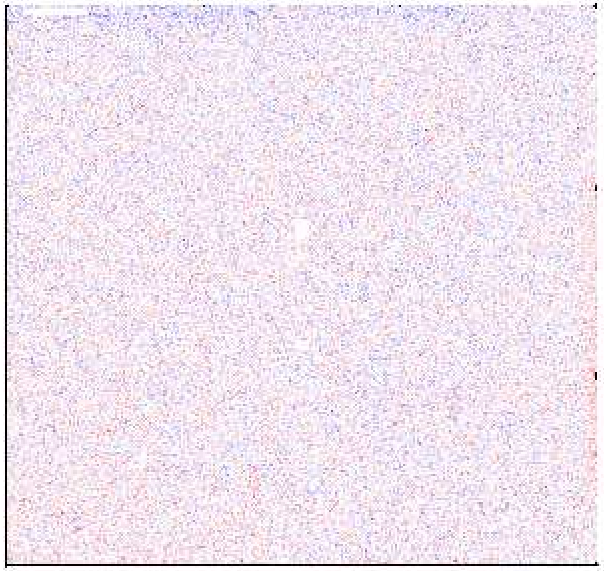

fitPLM residuals image plot: 2/38 (Thu Mar 12 14:57:16 2009)

A143_07_Nugo_009_t0.CEL

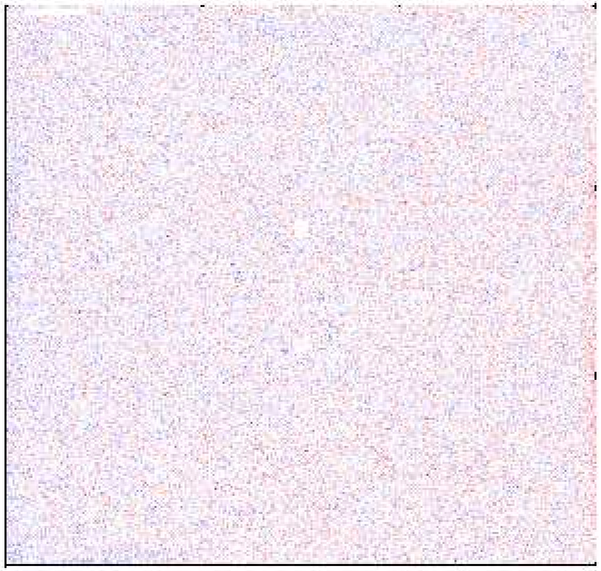

fitPLM residuals image plot: 3/38 (Thu Mar 12 14:57:20 2009)

A143_08_Nugo_009_t2.CEL

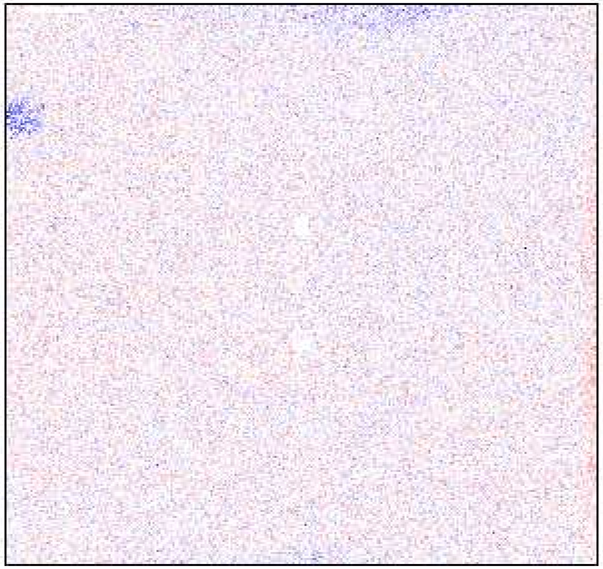

fitPLM residuals image plot: 4/38 (Thu Mar 12 14:57:24 2009)

A143_09_Nugo_014_t0.CEL

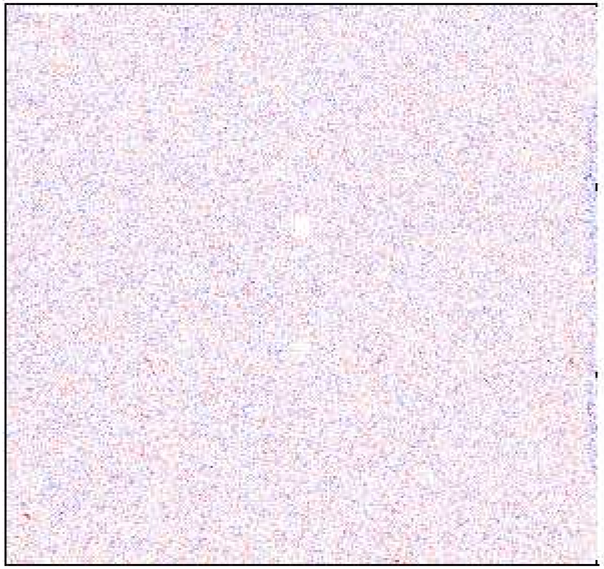

fitPLM residuals image plot: 5/38 (Thu Mar 12 14:57:27 2009)

A143_10_Nugo_014_t2.CEL

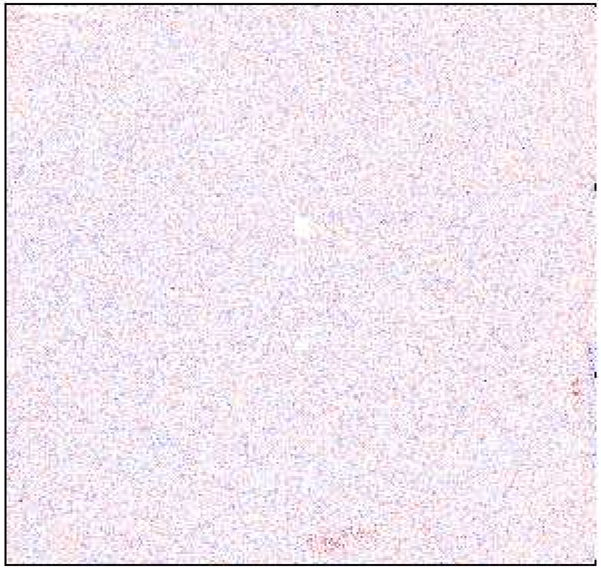

fitPLM residuals image plot: 6/38 (Thu Mar 12 14:57:31 2009)

A143_11_Nugo_016_t0.CEL

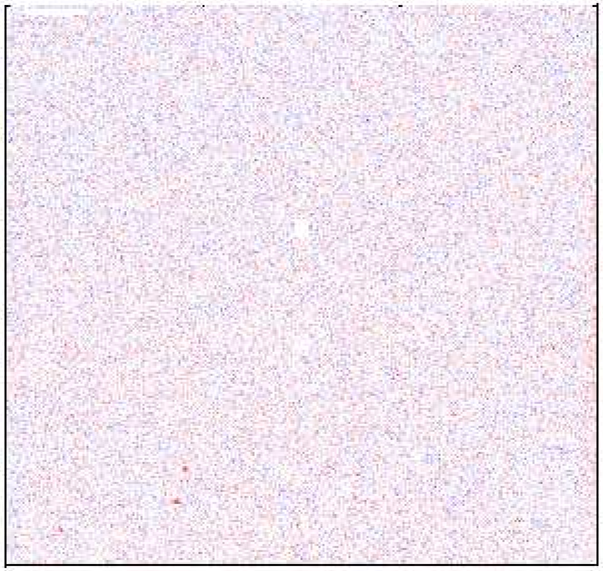

fitPLM residuals image plot: 7/38 (Thu Mar 12 14:57:38 2009)

A143_12_Nugo_016_t2.CEL

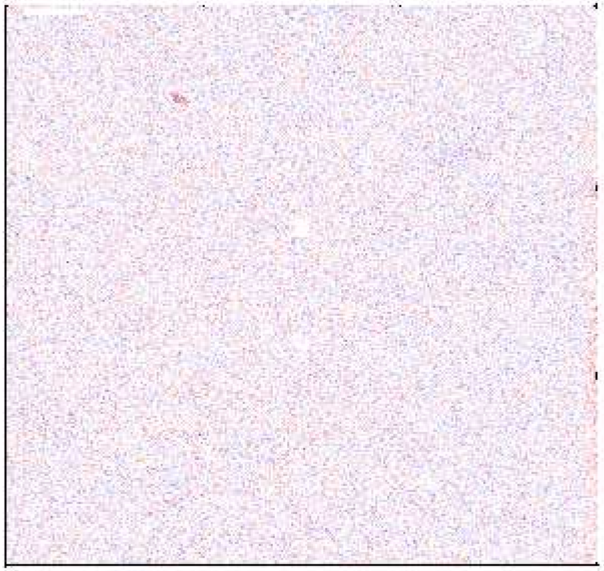

fitPLM residuals image plot: 8/38 (Thu Mar 12 14:57:41 2009)

A143_13_Nugo_017_t0.CEL

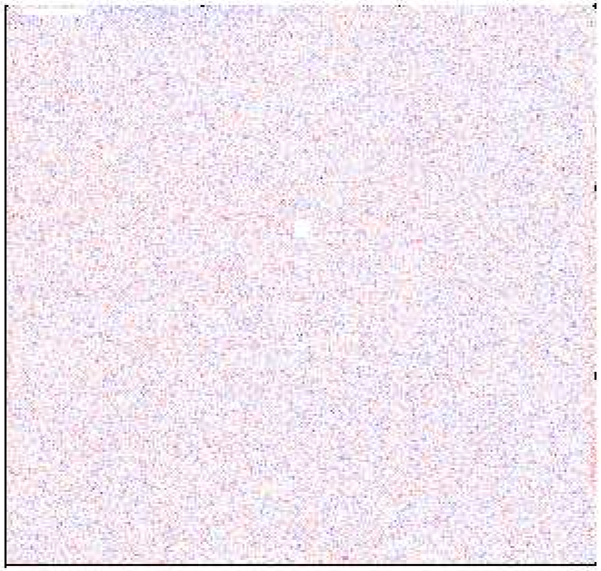

fitPLM residuals image plot: 9/38 (Thu Mar 12 14:57:45 2009)

A143_14_Nugo_017_t2.CEL

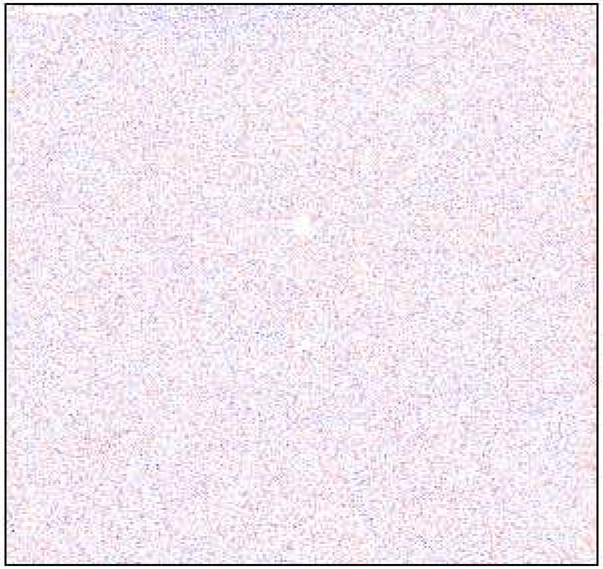

fitPLM residuals image plot: 10/38 (Thu Mar 12 14:57:49 2009)

A143_15_Nugo_018_t0.CEL

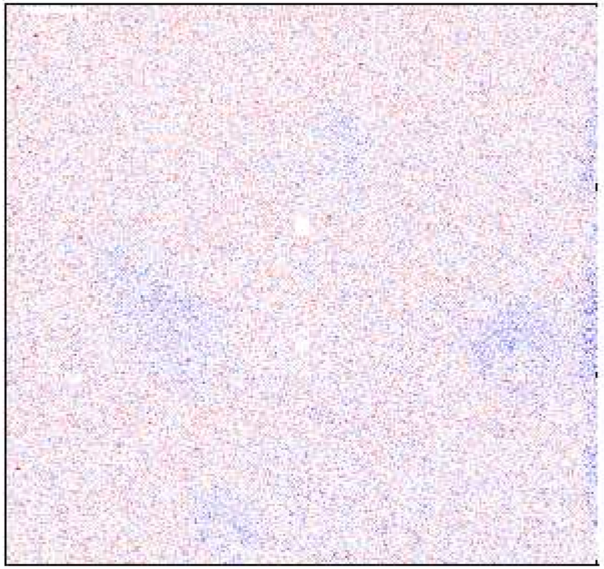

fitPLM residuals image plot: 11/38 (Thu Mar 12 14:57:53 2009)

A143_16_Nugo_018_t2.CEL

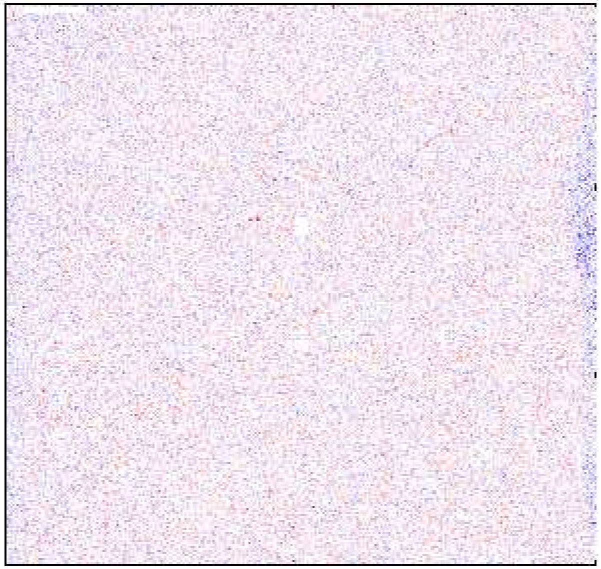

fitPLM residuals image plot: 12/38 (Thu Mar 12 14:57:57 2009)

A143_17_Nugo_019_t0.CEL

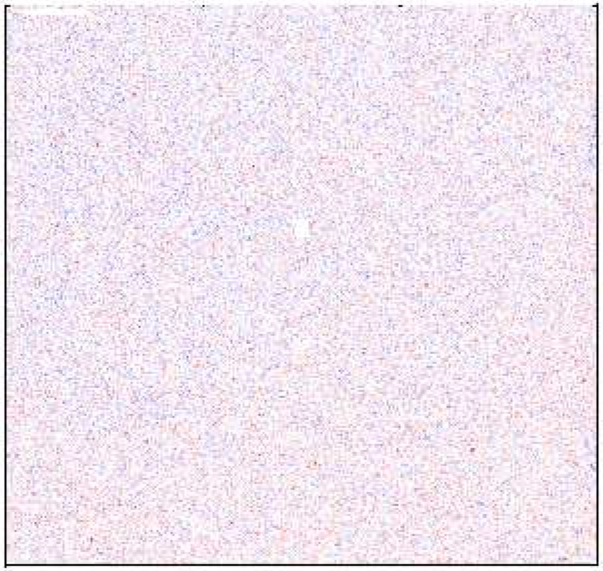

fitPLM residuals image plot: 13/38 (Thu Mar 12 14:58:03 2009)

A143_18_Nugo_019_t2.CEL

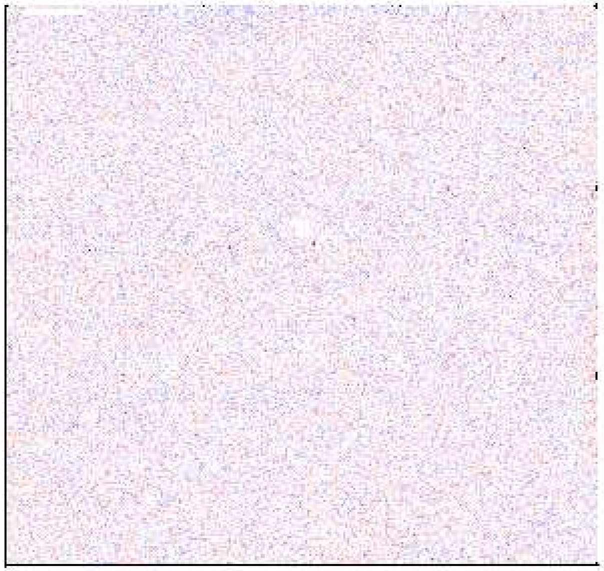

fitPLM residuals image plot: 14/38 (Thu Mar 12 14:58:07 2009)

A143_19_Nugo_020_t0.CEL

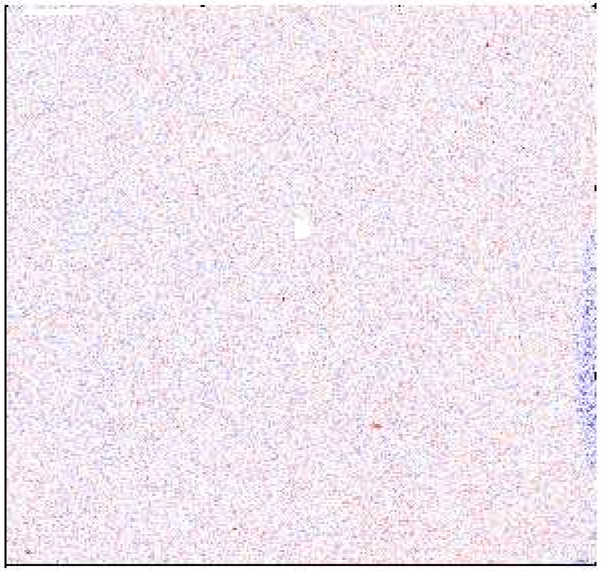

fitPLM residuals image plot: 15/38 (Thu Mar 12 14:58:10 2009)

A143_20_Nugo_020_t2.CEL

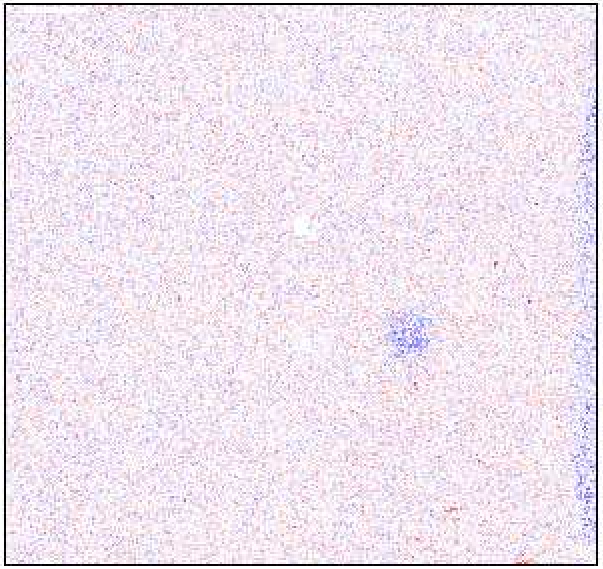

fitPLM residuals image plot: 16/38 (Thu Mar 12 14:58:14 2009)

A143_23_Nugo_024_t0.CEL

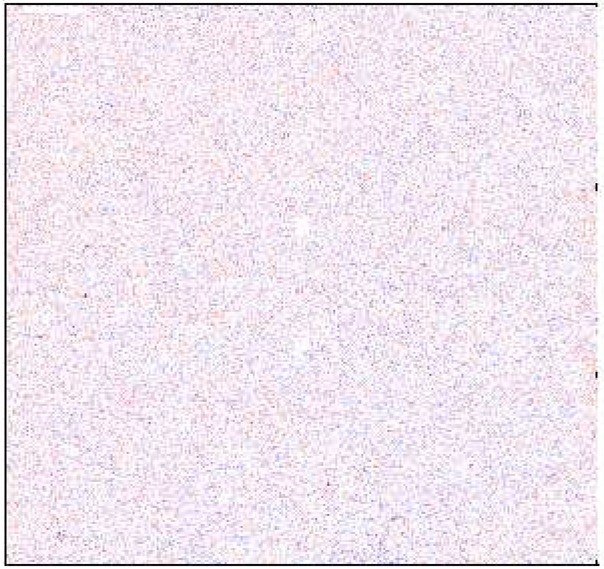

fitPLM residuals image plot: 17/38 (Thu Mar 12 14:58:19 2009)

A143_24_Nugo_024_t2.CEL

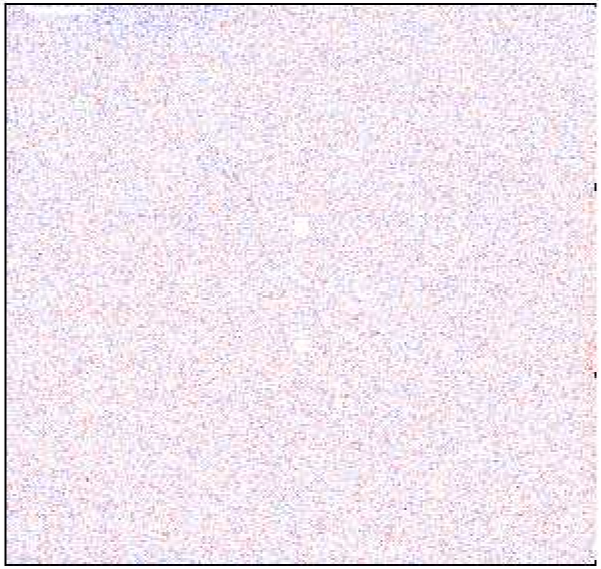

fitPLM residuals image plot: 18/38 (Thu Mar 12 14:58:23 2009)

A143_25_Nugo_029_t0.CEL

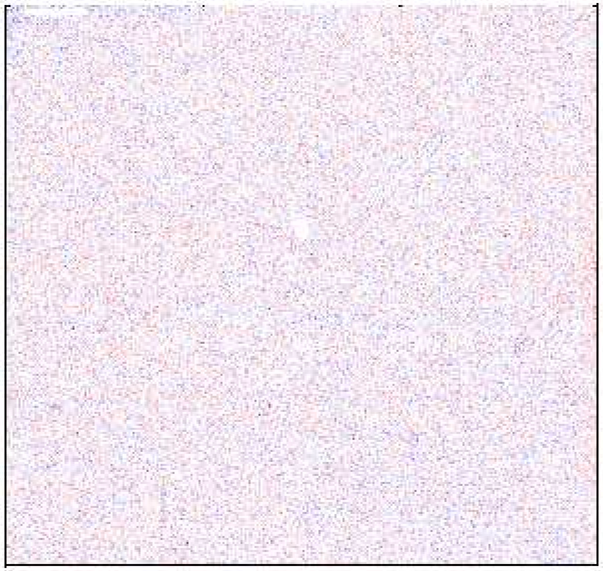

fitPLM residuals image plot: 19/38 (Thu Mar 12 14:58:28 2009)

A143_26_Nugo_029_t2.CEL

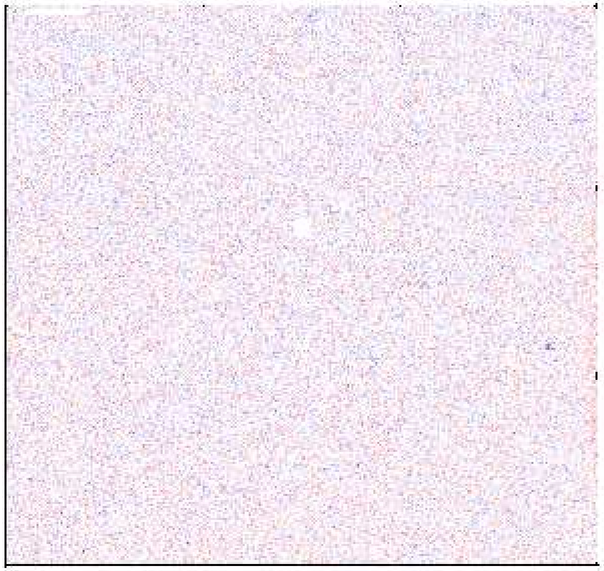

fitPLM residuals image plot: 20/38 (Thu Mar 12 14:58:32 2009)

A143_27_Nugo_030_t0.CEL

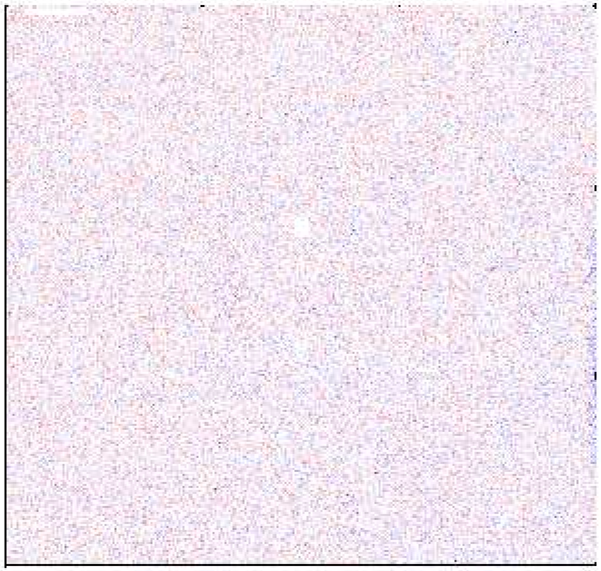

fitPLM residuals image plot: 21/38 (Thu Mar 12 14:58:36 2009)

A143_28_Nugo_030_t2.CEL

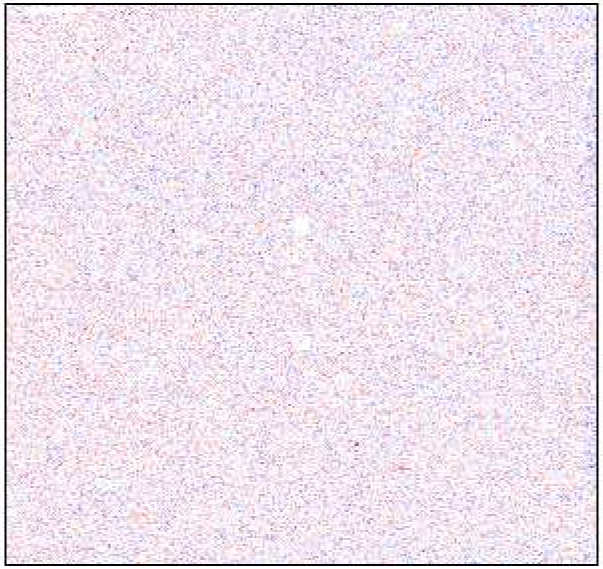

fitPLM residuals image plot: 22/38 (Thu Mar 12 14:58:40 2009)

A143_29_Nugo_031_t0.CEL

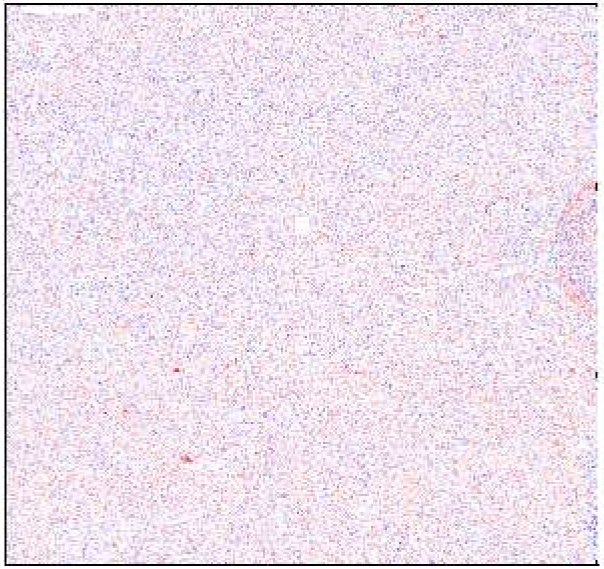

fitPLM residuals image plot: 23/38 (Thu Mar 12 14:58:44 2009)

A143_30_Nugo_031_t2.CEL

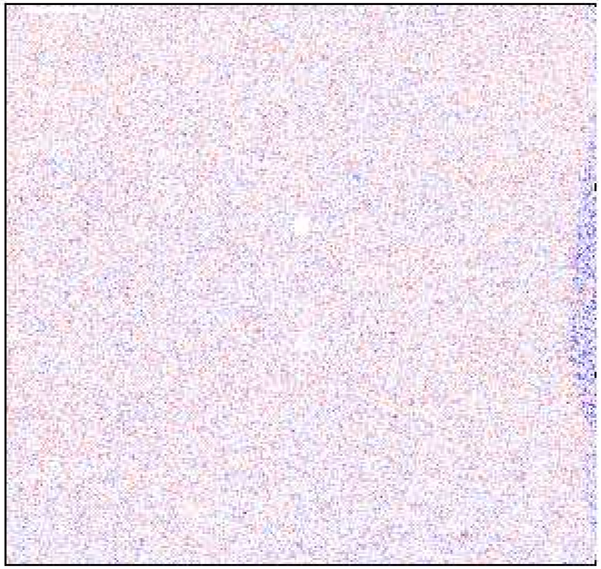

fitPLM residuals image plot: 24/38 (Thu Mar 12 14:58:47 2009)

A143_31_Nugo_033_t0.CEL

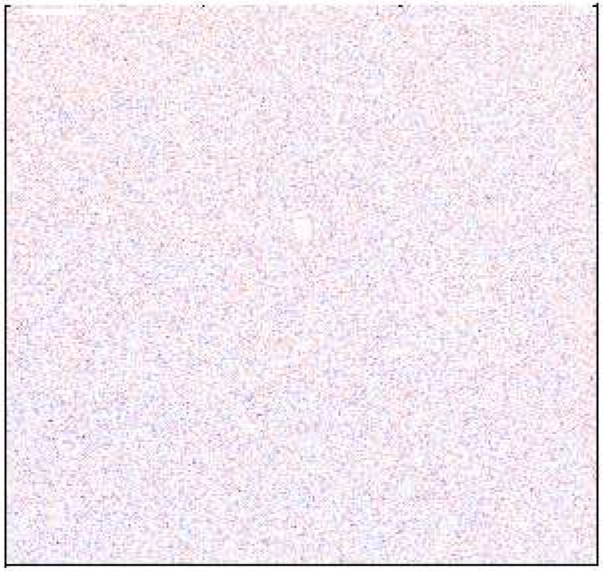

fitPLM residuals image plot: 25/38 (Thu Mar 12 14:58:53 2009)

A143_32_Nugo_033_t2.CEL

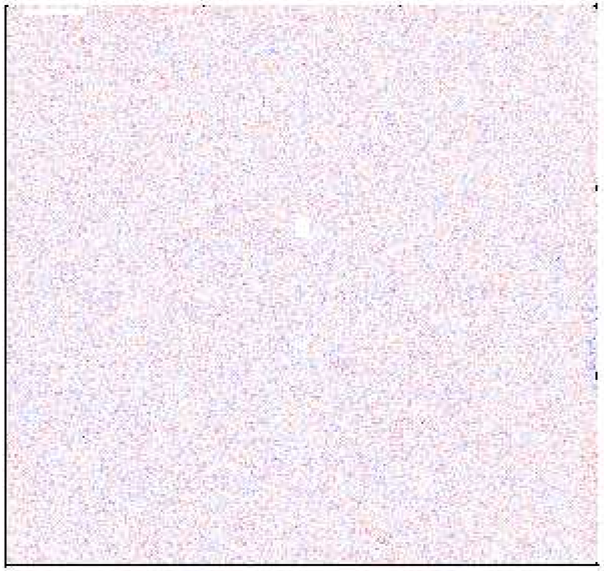

fitPLM residuals image plot: 26/38 (Thu Mar 12 14:58:57 2009)

A143_33_Nugo_038_t0.CEL

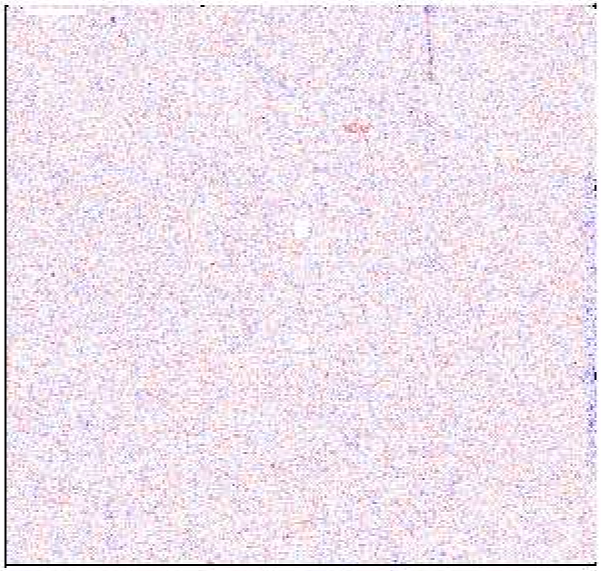

fitPLM residuals image plot: 27/38 (Thu Mar 12 14:59:01 2009)

A143_34_Nugo_038_t2.CEL

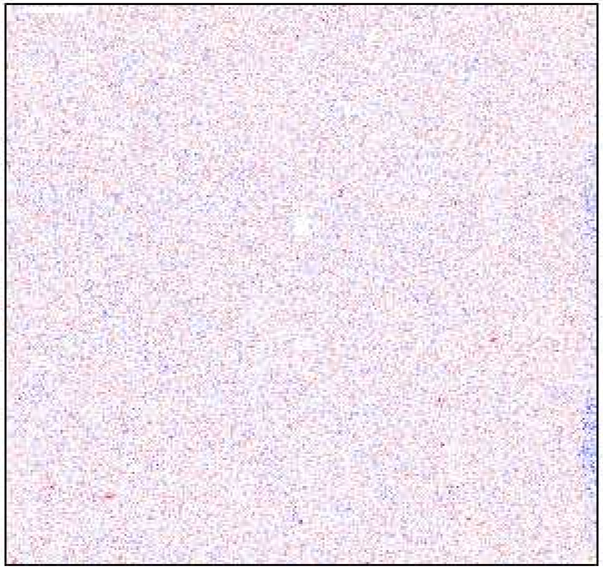

fitPLM residuals image plot: 28/38 (Thu Mar 12 14:59:05 2009)

A143_35_Nugo_039_t0.CEL

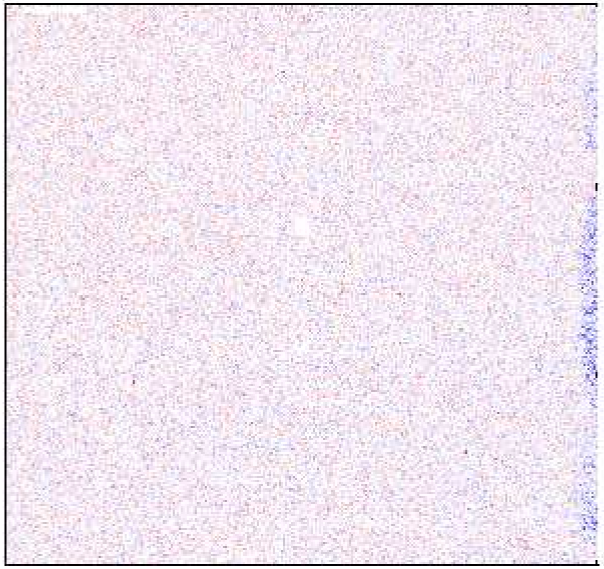

fitPLM residuals image plot: 29/38 (Thu Mar 12 14:59:08 2009)

A143_36_Nugo_039_t2.CEL

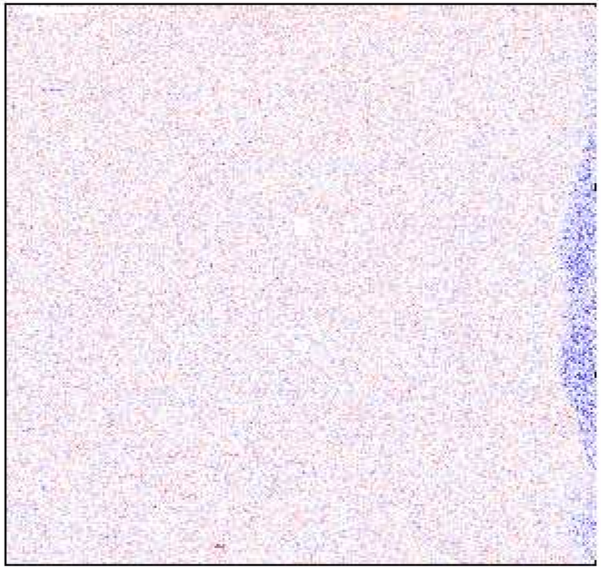

fitPLM residuals image plot: 30/38 (Thu Mar 12 14:59:13 2009)

A143_37_Nugo_044_t0.CEL

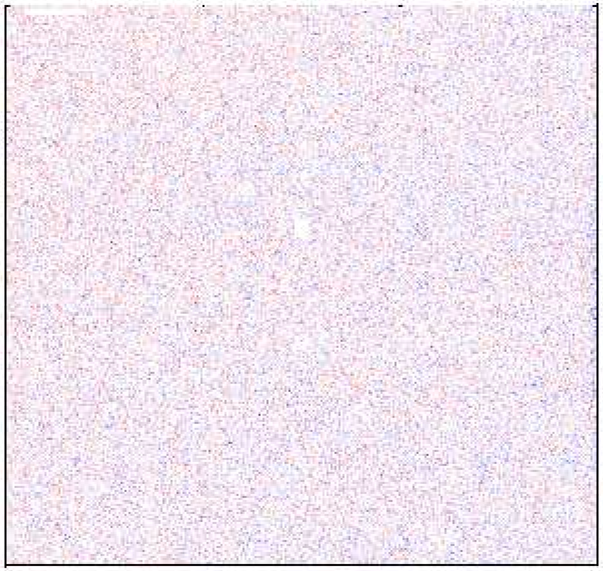

fitPLM residuals image plot: 31/38 (Thu Mar 12 14:59:18 2009)

A143_38_Nugo_044_t2.CEL

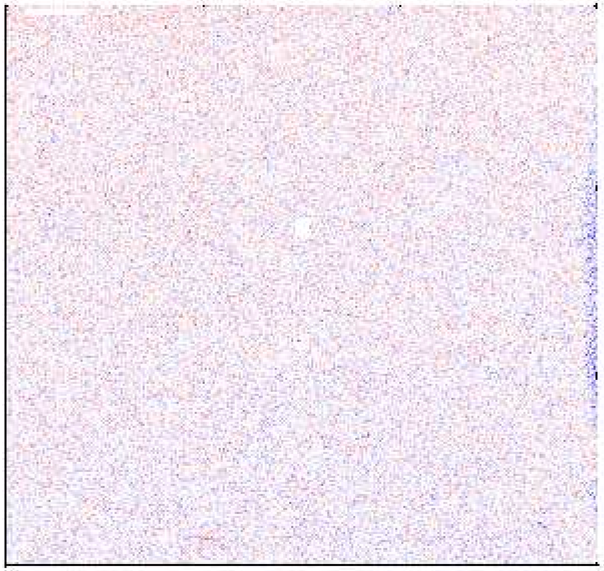

fitPLM residuals image plot: 32/38 (Thu Mar 12 14:59:21 2009)

A143_39_Nugo_045_t0.CEL

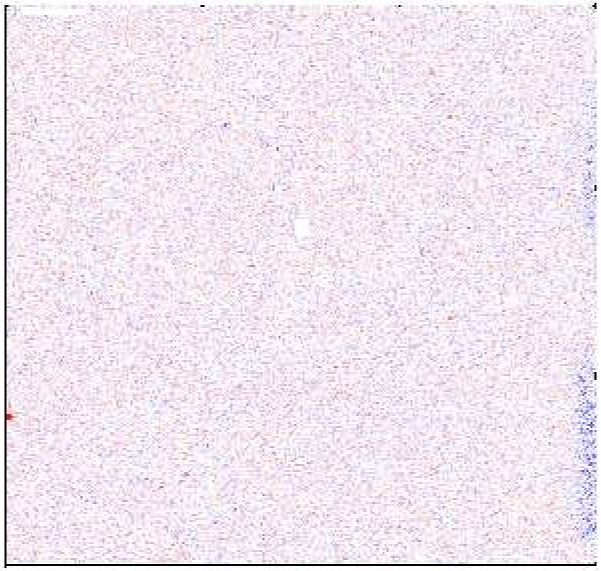

fitPLM residuals image plot: 33/38 (Thu Mar 12 14:59:25 2009)

A143_40_Nugo_045_t2.CEL

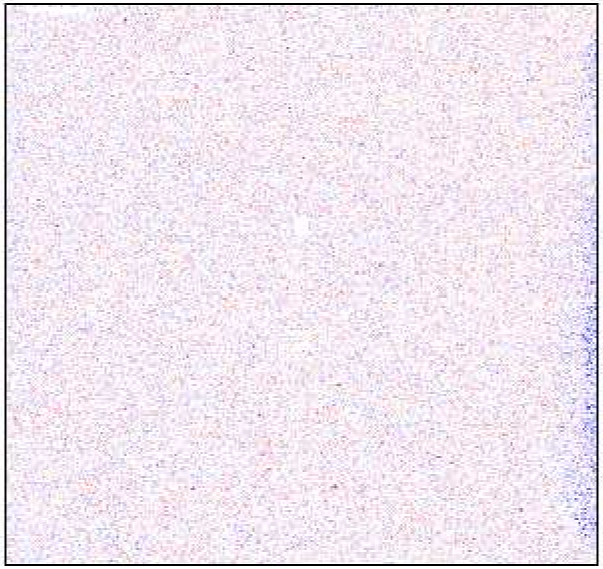

fitPLM residuals image plot: 34/38 (Thu Mar 12 14:59:29 2009)

A143_41_Nugo_048_t0.CEL

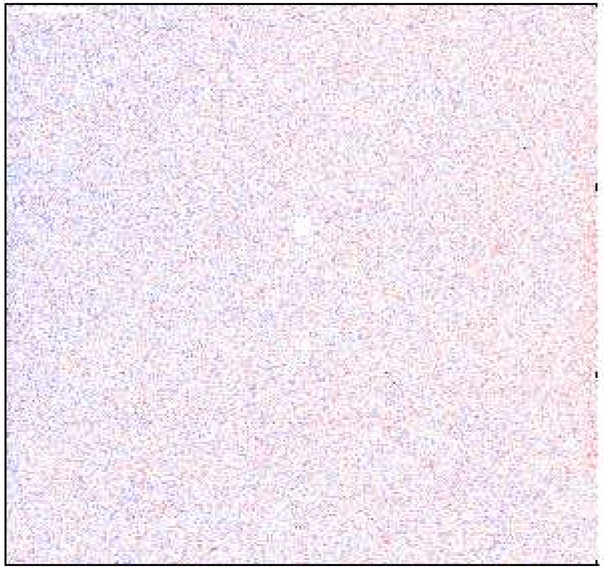

fitPLM residuals image plot: 35/38 (Thu Mar 12 14:59:33 2009)

A143_42_Nugo_048_t2.CEL

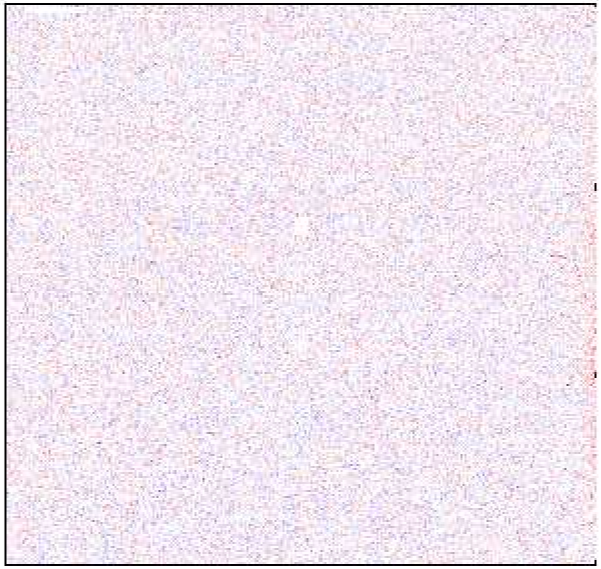

fitPLM residuals image plot: 36/38 (Thu Mar 12 14:59:37 2009)

A143_43A_Nugo_053_t0.CEL

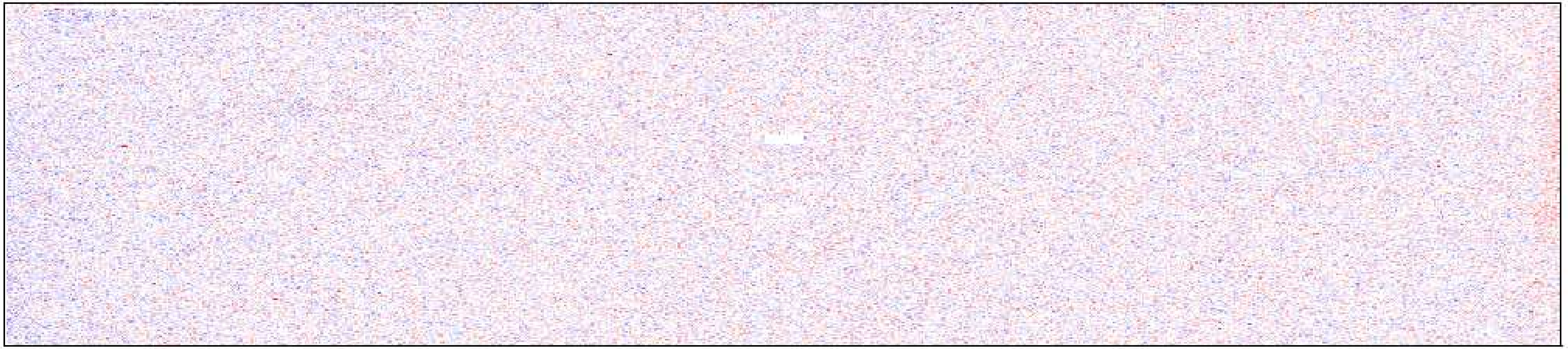

fitPLM residuals image plot: 37/38 (Thu Mar 12 14:59:42 2009)

A143_44A_Nugo_053_t2.CEL

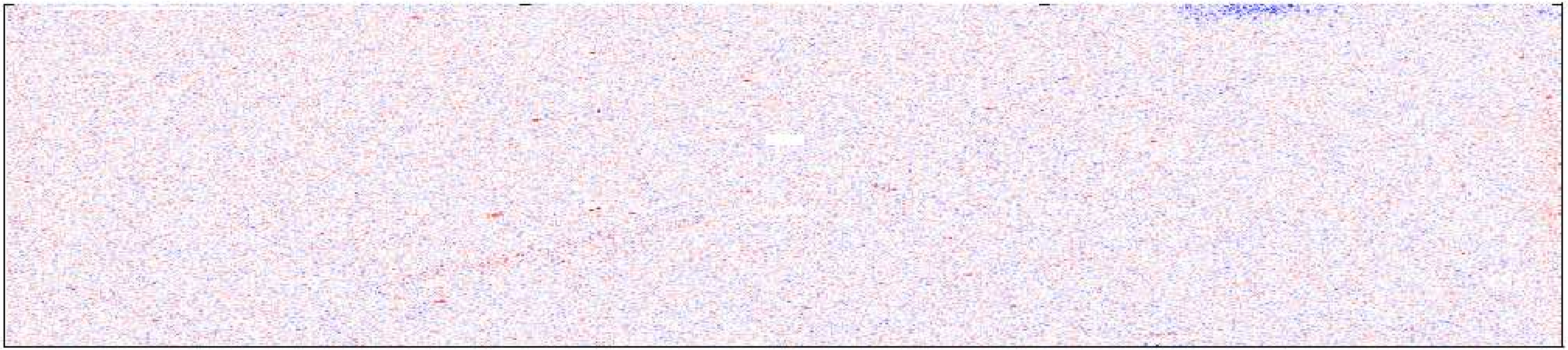

fitPLM residuals image plot: 38/38 (Thu Mar 12 14:59:47 2009)

Density plot (1/7) BEFORE normalization. Date: Thu Mar 12 15:01:42 2009

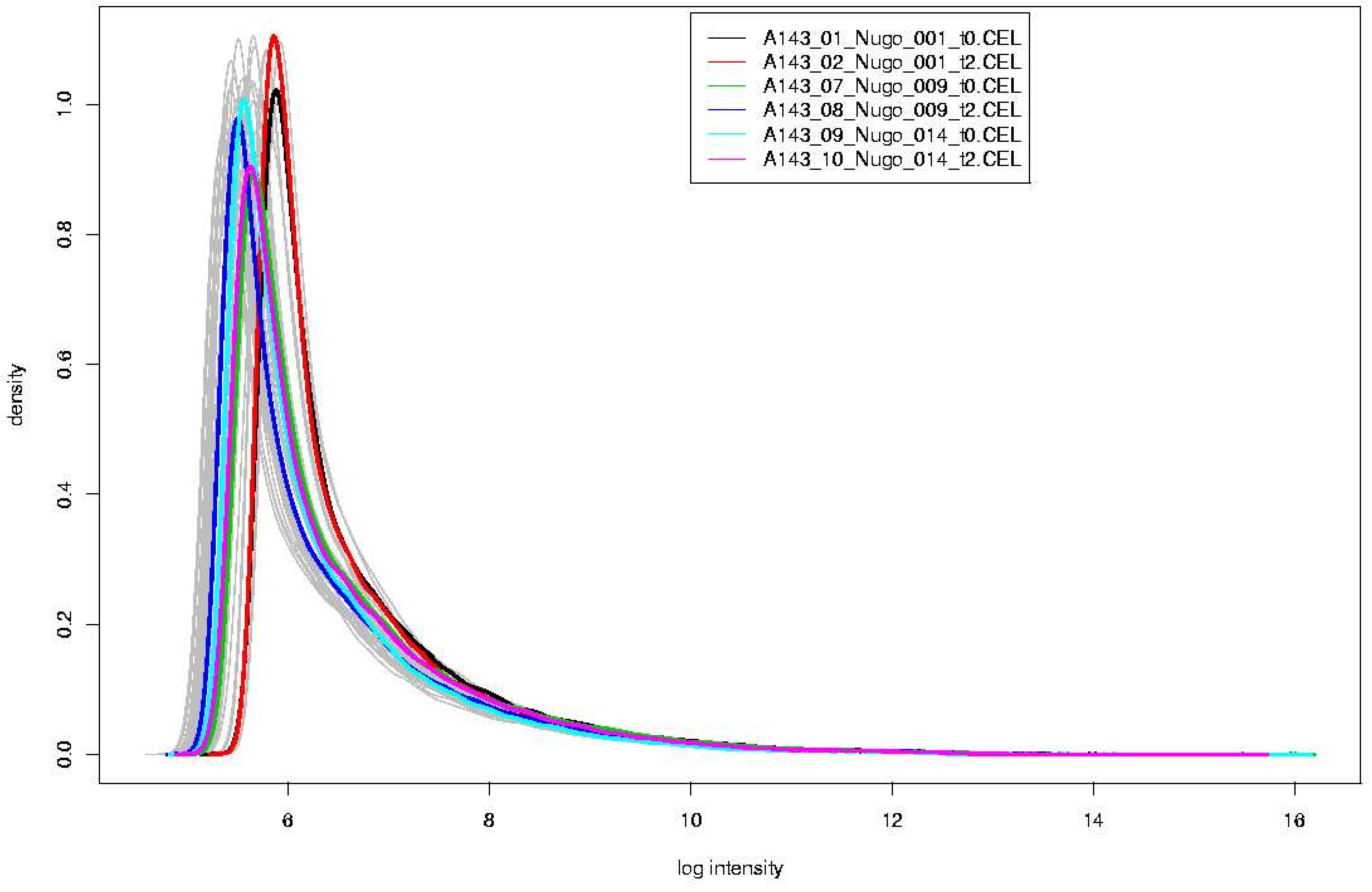

Density plot (2/7) BEFORE normalization. Date: Thu Mar 12 15:01:58 2009

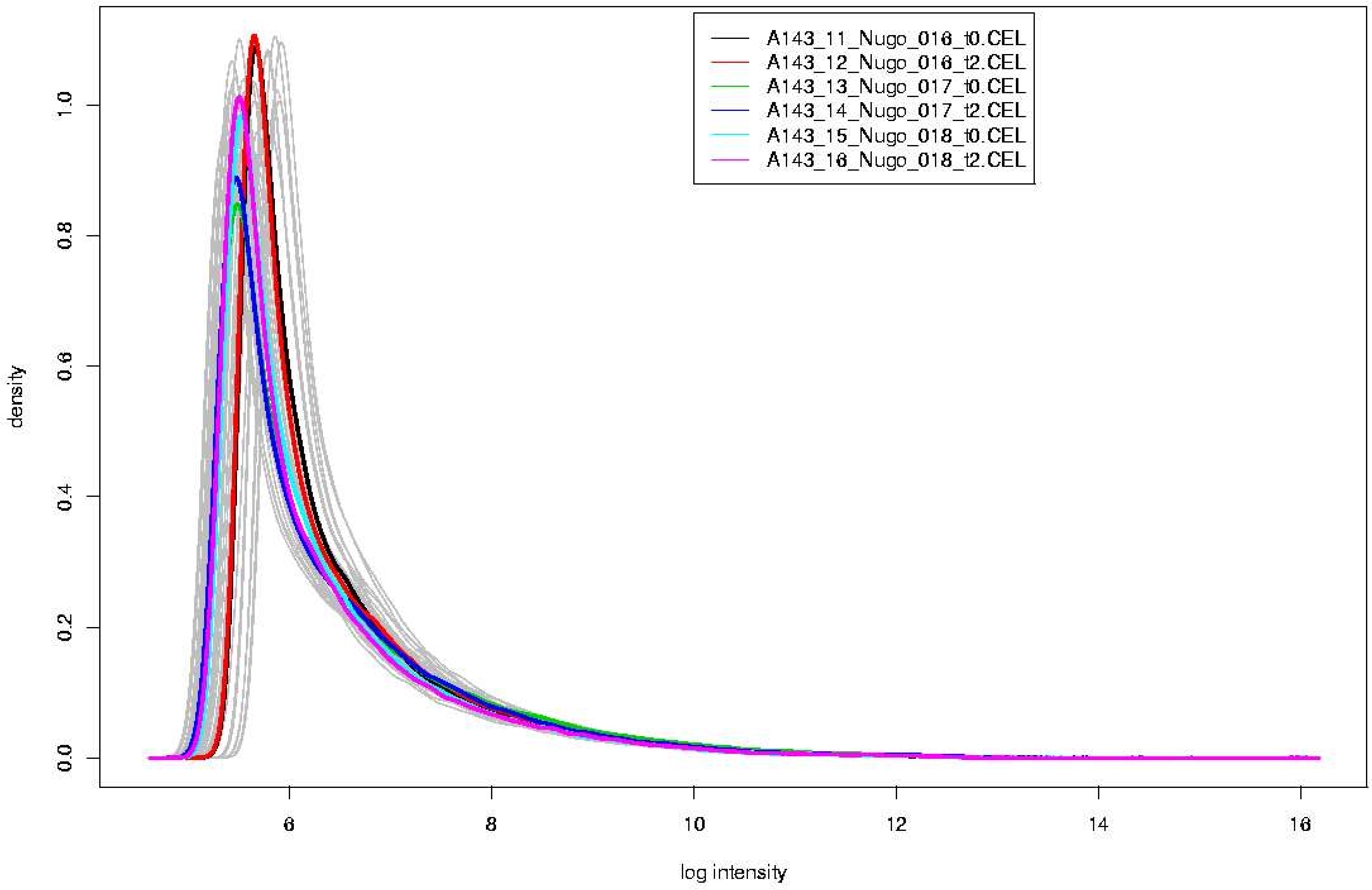

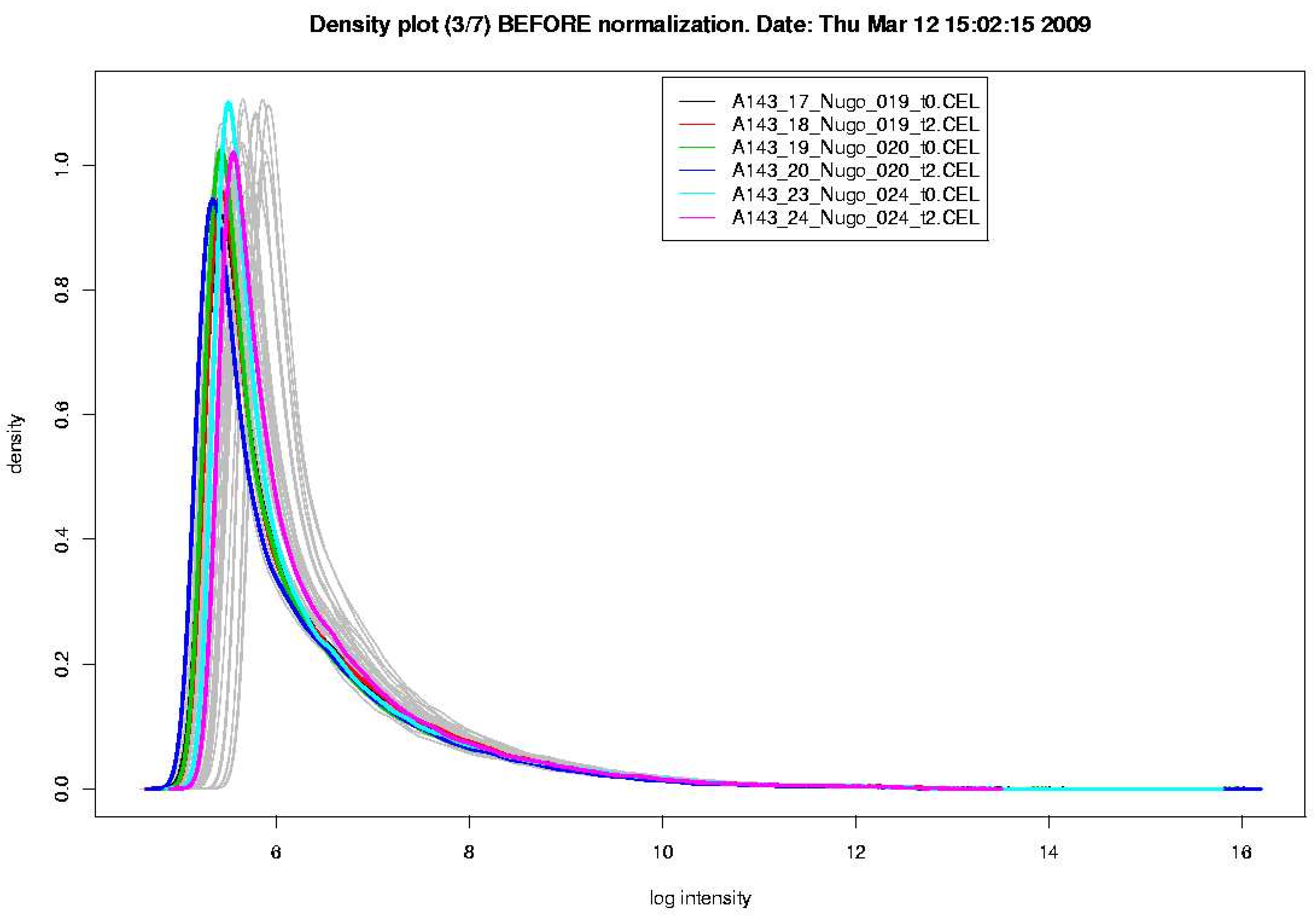
Density plot (3/7) BEFORE normalization. Date: Thu Mar 12 15:02:15 2009
A143_17_Nugo_019_t0.CEL
A143_18_Nugo_019_t2.CEL
A143_19_Nugo_020_t0.CEL
A143_20_Nugo_020_t2.CEL
A143_23_Nugo_024_t0.CEL
A143_24_Nugo_024_t2.CEL
density
0.0
0.2
0.4
0.6
0.8
1.0
log intensity
6
8
10
12
14
16

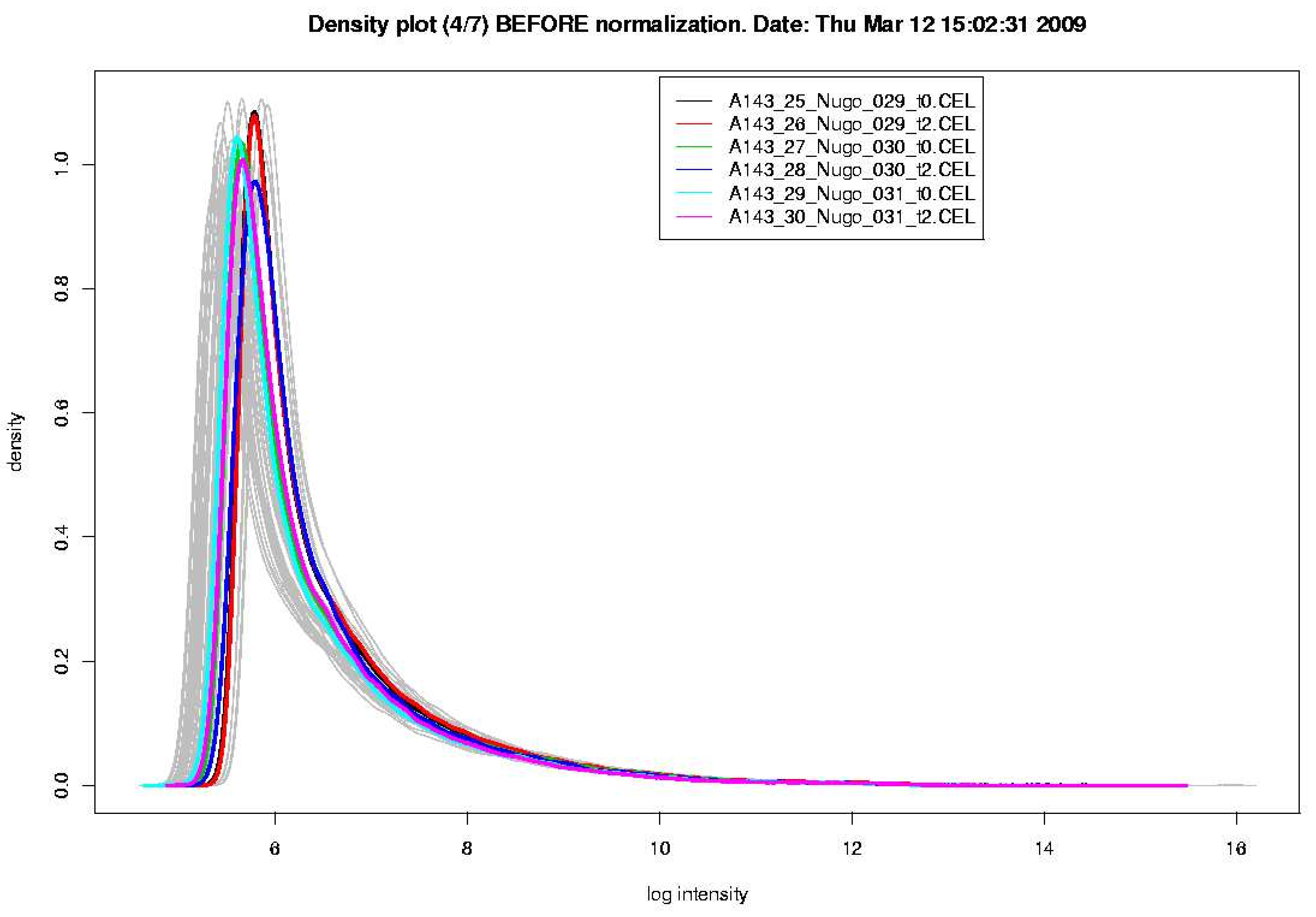
Density plot (4/7) BEFORE normalization. Date: Thu Mar 12 15:02:31 2009
density
0.0
0.2
0.4
0.6
0.8
1.0
log intensity
6
8
10
12
14
16
A143_25_Nugo_029_t0.CEL
A143_26_Nugo_029_t2.CEL
A143_27_Nugo_030_t0.CEL
A143_28_Nugo_030_t2.CEL
A143_29_Nugo_031_t0.CEL
A143_30_Nugo_031_t2.CEL

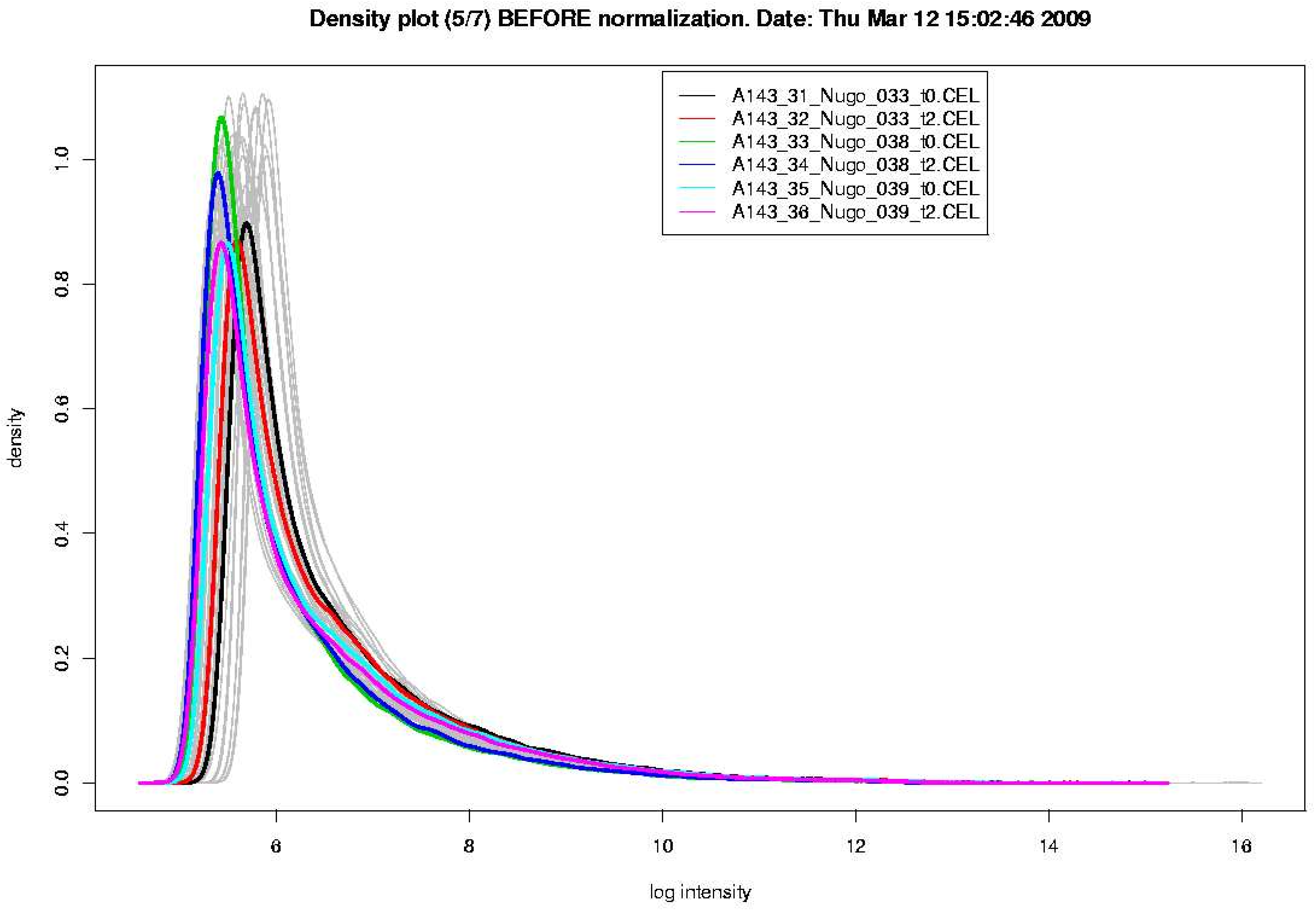
Density plot (5/7) BEFORE normalization. Date: Thu Mar 12 15:02:46 2009
A143_31_Nugo_033_t0.CEL
A143_32_Nugo_033_t2.CEL
A143_33_Nugo_038_t0.CEL
A143_34_Nugo_038_t2.CEL
A143_35_Nugo_039_t0.CEL
A143_36_Nugo_039_t2.CEL
density
0.0
0.2
0.4
0.6
0.8
1.0
log intensity
6
8
10
12
14
16

Density plot (6/7) BEFORE normalization. Date: Thu Mar 12 15:03:01 2009

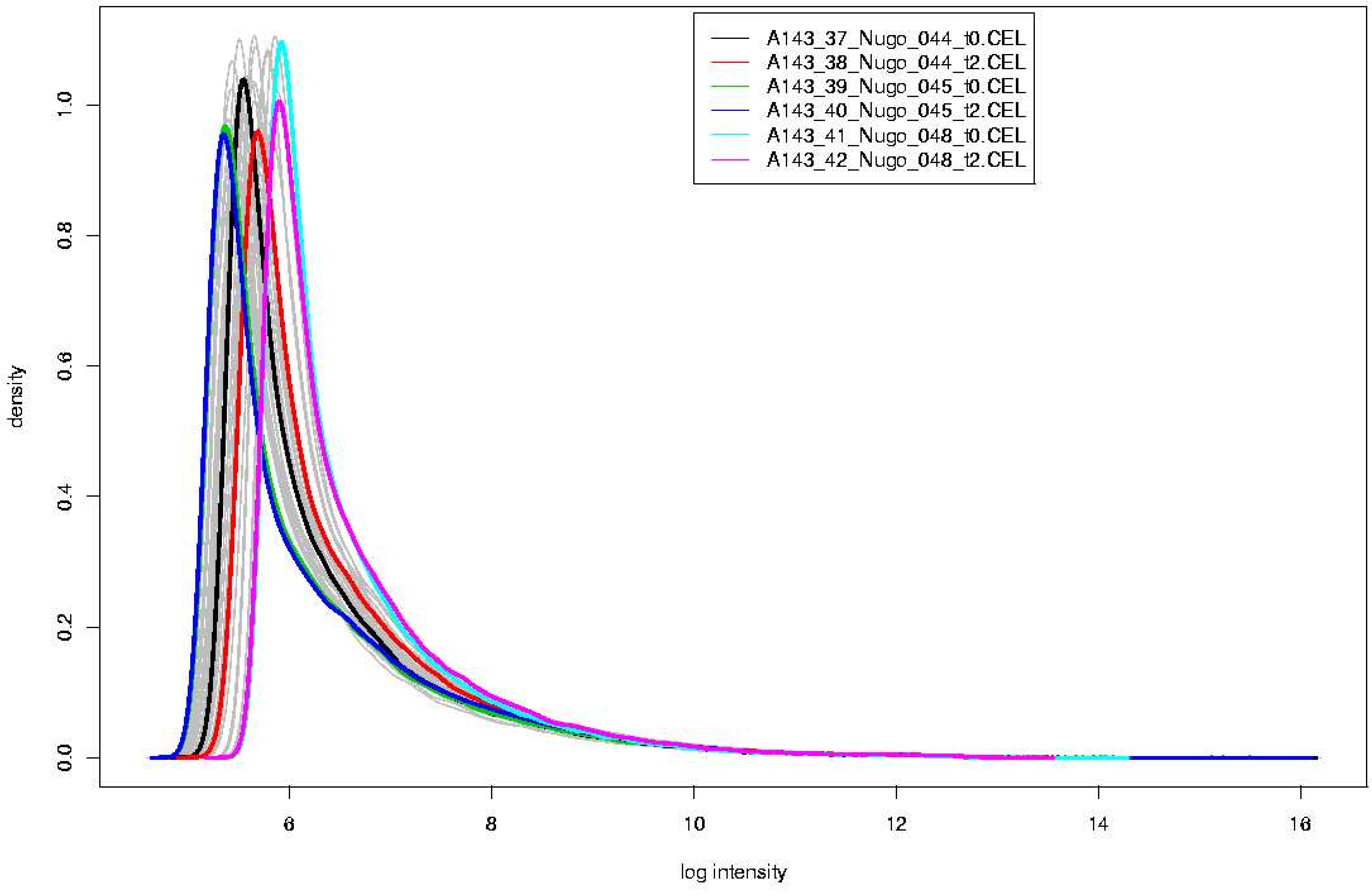

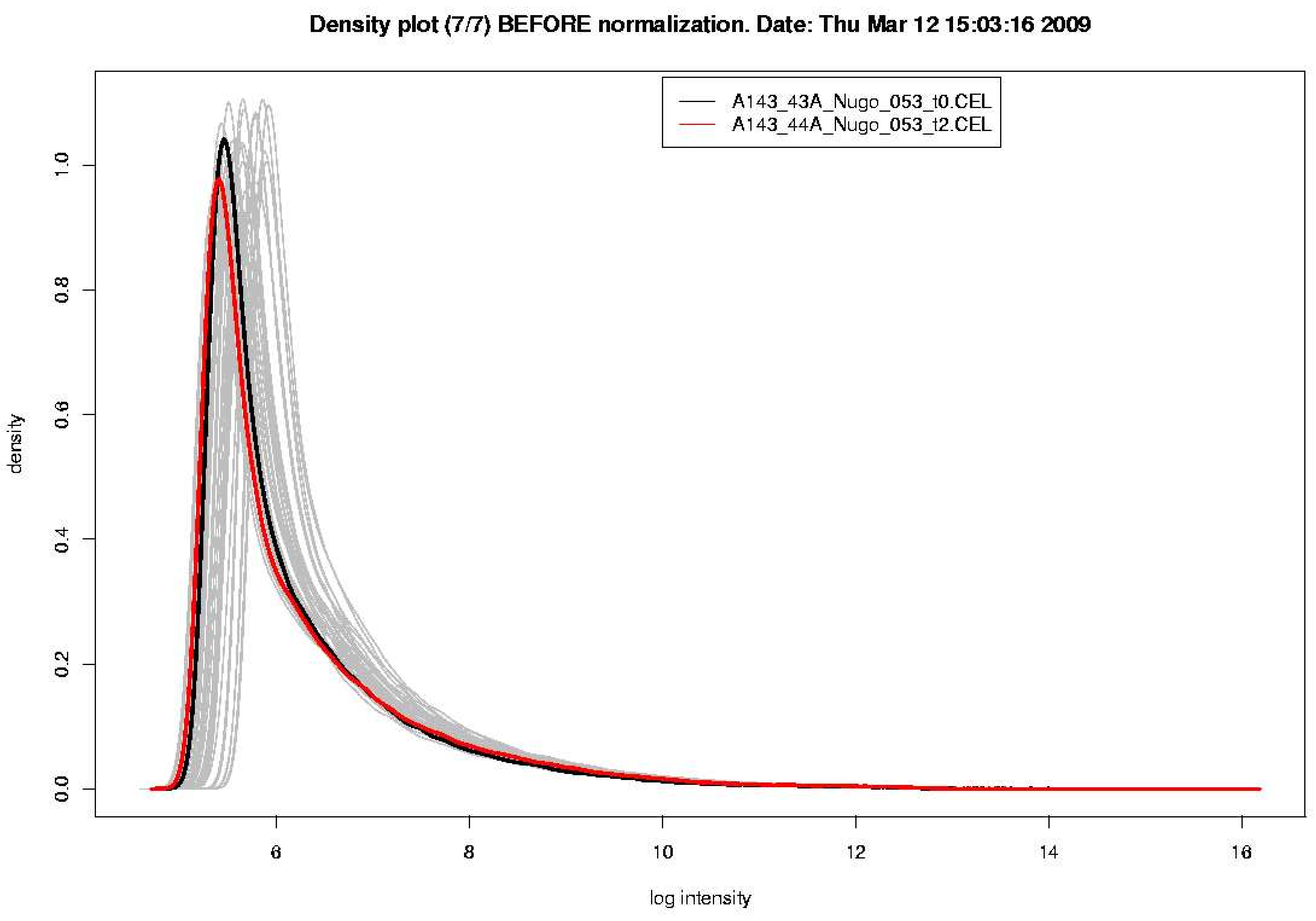
Density plot (7/7) BEFORE normalization. Date: Thu Mar 12 15:03:16 2009
A143_43A_Nugo_053_t0.CEL
A143_44A_Nugo_053_t2.CEL
density
0.0
0.2
0.4
0.6
0.8
1.0
6
8
10
12
14
16
log intensity

Density plot of ALL arrays BEFORE normalization. Date: Thu Mar 12 15:03:29 2009

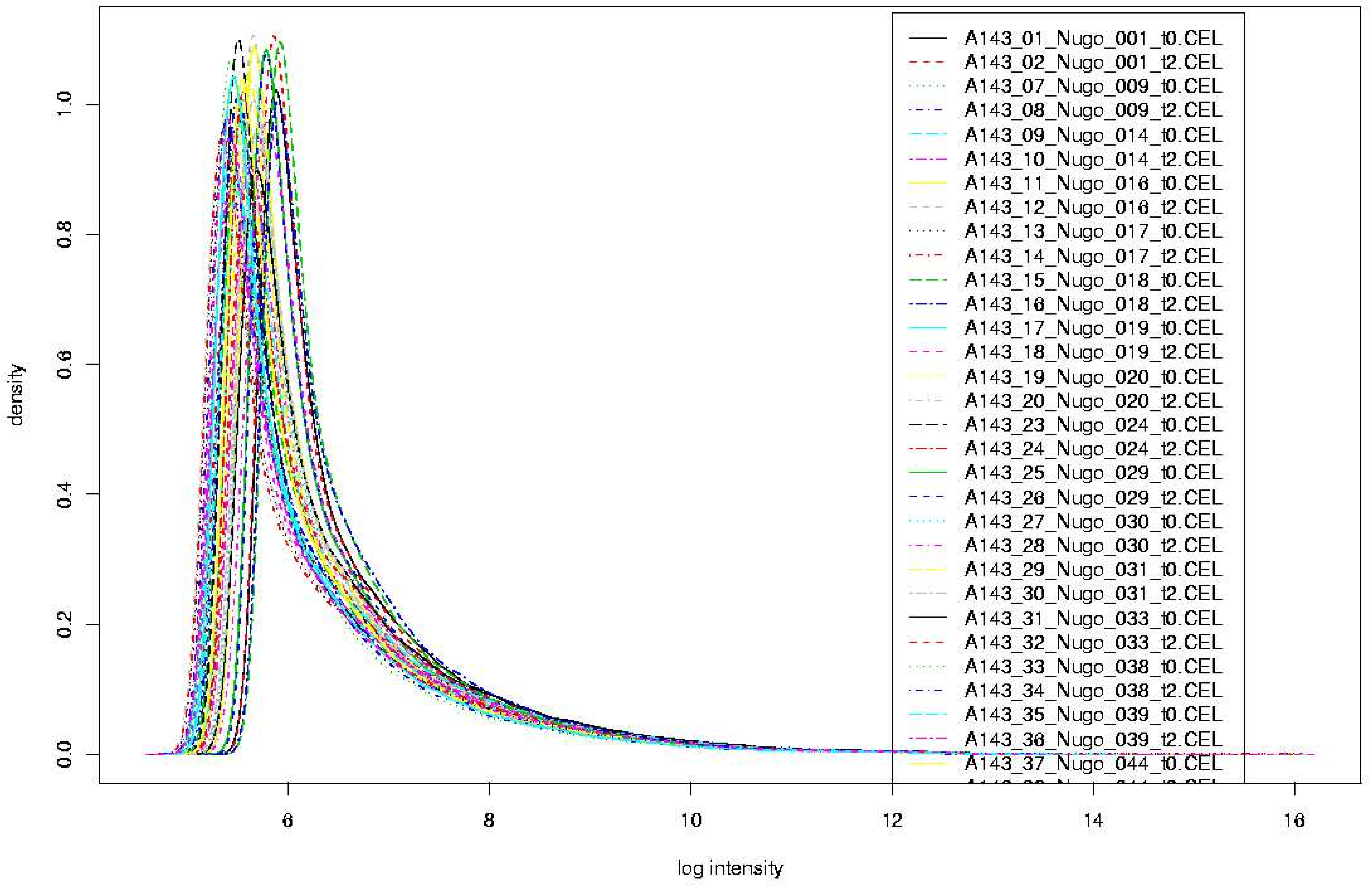

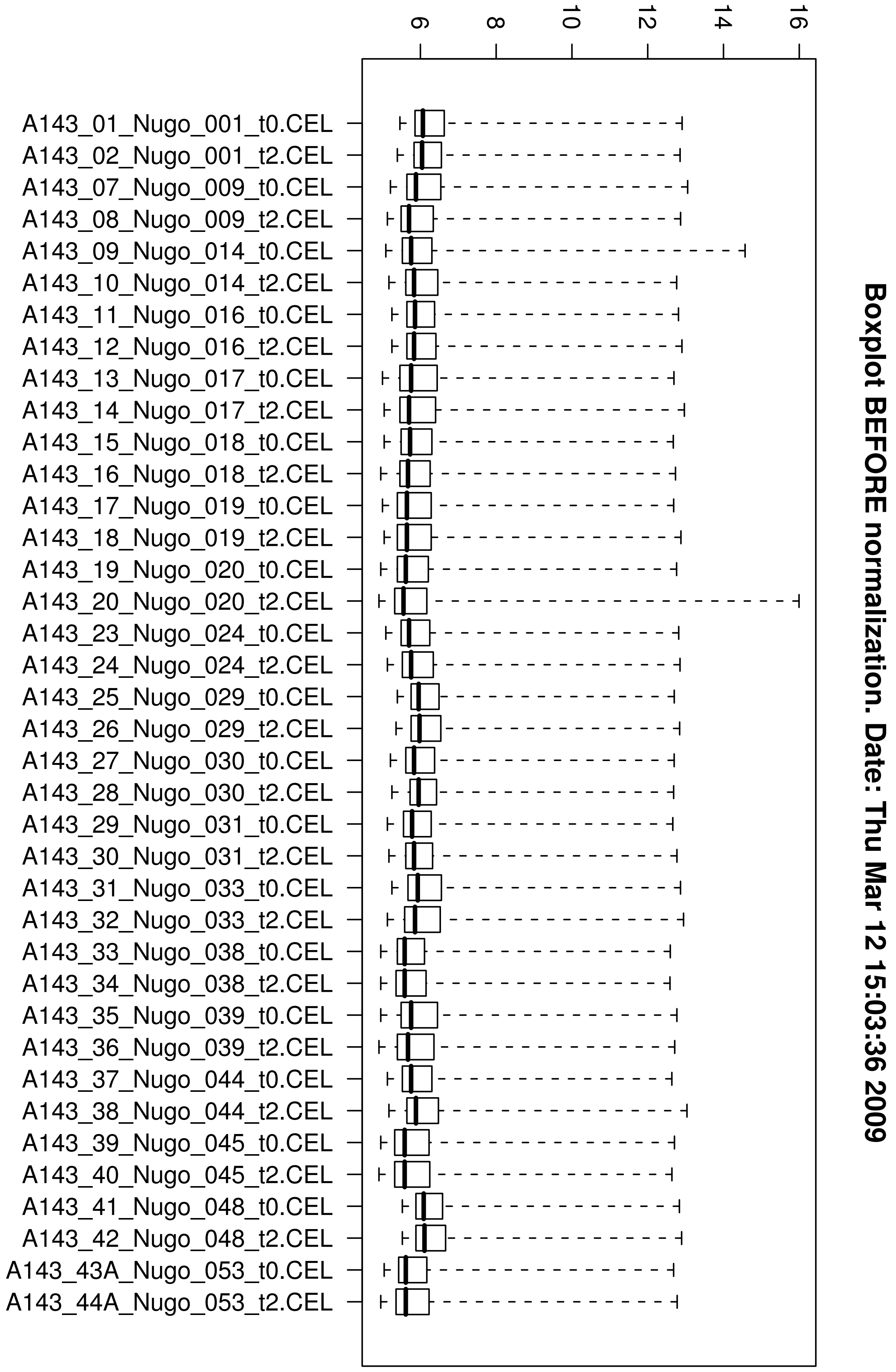
Boxplot BEFORE normalization. Date: Thu Mar 12 15:03:36 2009
6
8
10
12
14
16
A143_01_Nugo_001_t0.CEL
A143_02_Nugo_001_t2.CEL
A143_07_Nugo_009_t0.CEL
A143_08_Nugo_009_t2.CEL
A143_09_Nugo_014_t0.CEL
A143_10_Nugo_014_t2.CEL
A143_11_Nugo_016_t0.CEL
A143_12_Nugo_016_t2.CEL
A143_13_Nugo_017_t0.CEL
A143_14_Nugo_017_t2.CEL
A143_15_Nugo_018_t0.CEL
A143_16_Nugo_018_t2.CEL
A143_17_Nugo_019_t0.CEL
A143_18_Nugo_019_t2.CEL
A143_19_Nugo_020_t0.CEL
A143_20_Nugo_020_t2.CEL
A143_23_Nugo_024_t0.CEL
A143_24_Nugo_024_t2.CEL
A143_25_Nugo_029_t0.CEL
A143_26_Nugo_029_t2.CEL
A143_27_Nugo_030_t0.CEL
A143_28_Nugo_030_t2.CEL
A143_29_Nugo_031_t0.CEL
A143_30_Nugo_031_t2.CEL
A143_31_Nugo_033_t0.CEL
A143_32_Nugo_033_t2.CEL
A143_33_Nugo_038_t0.CEL
A143_34_Nugo_038_t2.CEL
A143_35_Nugo_039_t0.CEL
A143_36_Nugo_039_t2.CEL
A143_37_Nugo_044_t0.CEL
A143_38_Nugo_044_t2.CEL
A143_39_Nugo_045_t0.CEL
A143_40_Nugo_045_t2.CEL
A143_41_Nugo_048_t0.CEL
A143_42_Nugo_048_t2.CEL
A143_43A_Nugo_053_t0.CEL
A143_44A_Nugo_053_t2.CEL

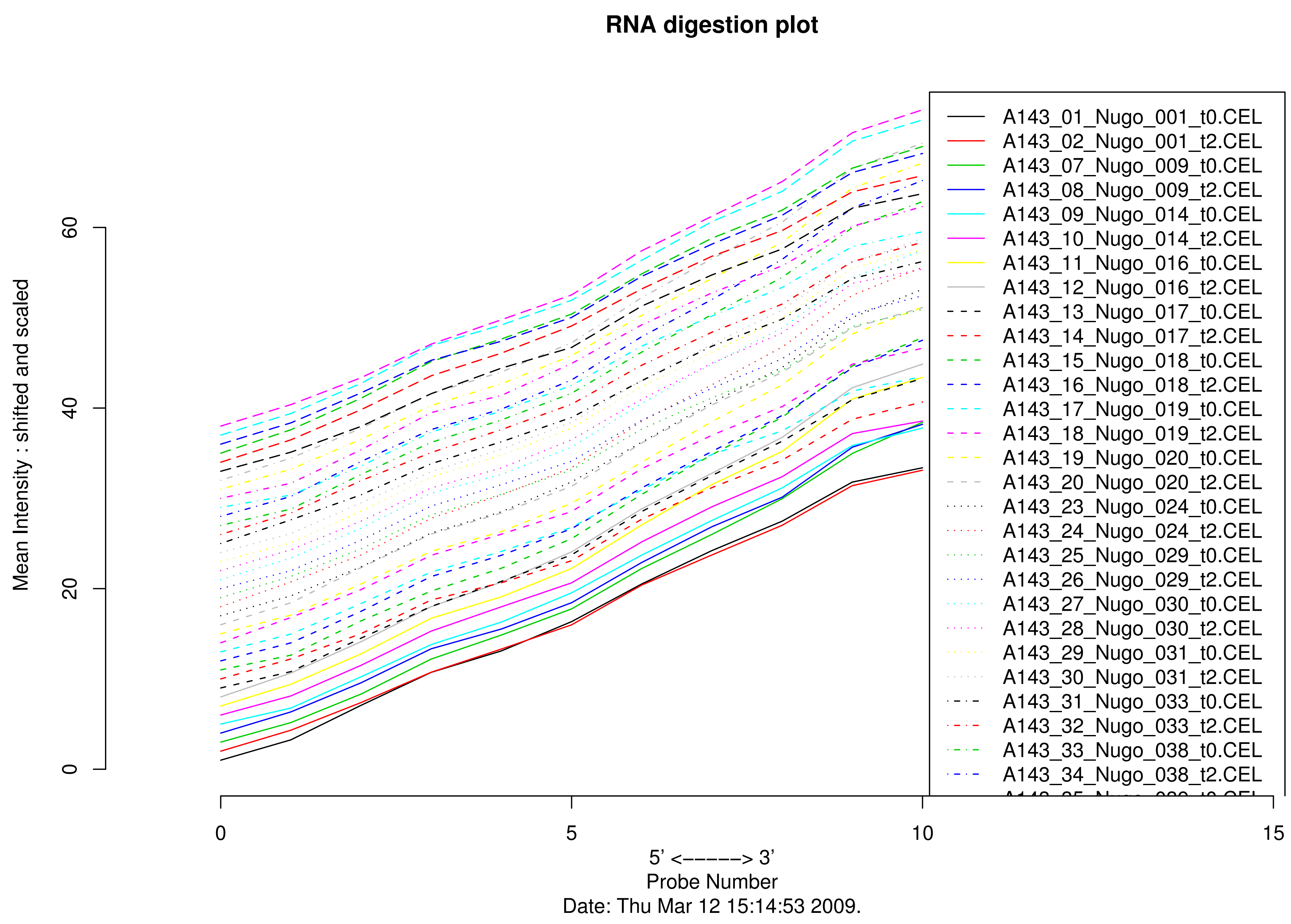

RNA digestion plot
Mean Intensity : shifted and scaled
0
20
40
60
0
5
10
15
A143_01_Nugo_001_t0.CEL
A143_02_Nugo_001_t2.CEL
A143_07_Nugo_009_t0.CEL
A143_08_Nugo_009_t2.CEL
A143_09_Nugo_014_t0.CEL
A143_10_Nugo_014_t2.CEL
A143_11_Nugo_016_t0.CEL
A143_12_Nugo_016_t2.CEL
A143_13_Nugo_017_t0.CEL
A143_14_Nugo_017_t2.CEL
A143_15_Nugo_018_t0.CEL
A143_16_Nugo_018_t2.CEL
A143_17_Nugo_019_t0.CEL
A143_18_Nugo_019_t2.CEL
A143_19_Nugo_020_t0.CEL
A143_20_Nugo_020_t2.CEL
A143_23_Nugo_024_t0.CEL
A143_24_Nugo_024_t2.CEL
A143_25_Nugo_029_t0.CEL
A143_26_Nugo_029_t2.CEL
A143_27_Nugo_030_t0.CEL
A143_28_Nugo_030_t2.CEL
A143_29_Nugo_031_t0.CEL
A143_30_Nugo_031_t2.CEL
A143_31_Nugo_033_t0.CEL
A143_32_Nugo_033_t2.CEL
A143_33_Nugo_038_t0.CEL
A143_34_Nugo_038_t2.CEL
5' <-----> 3'
Probe Number
Date: Thu Mar 12 15:14:53 2009.

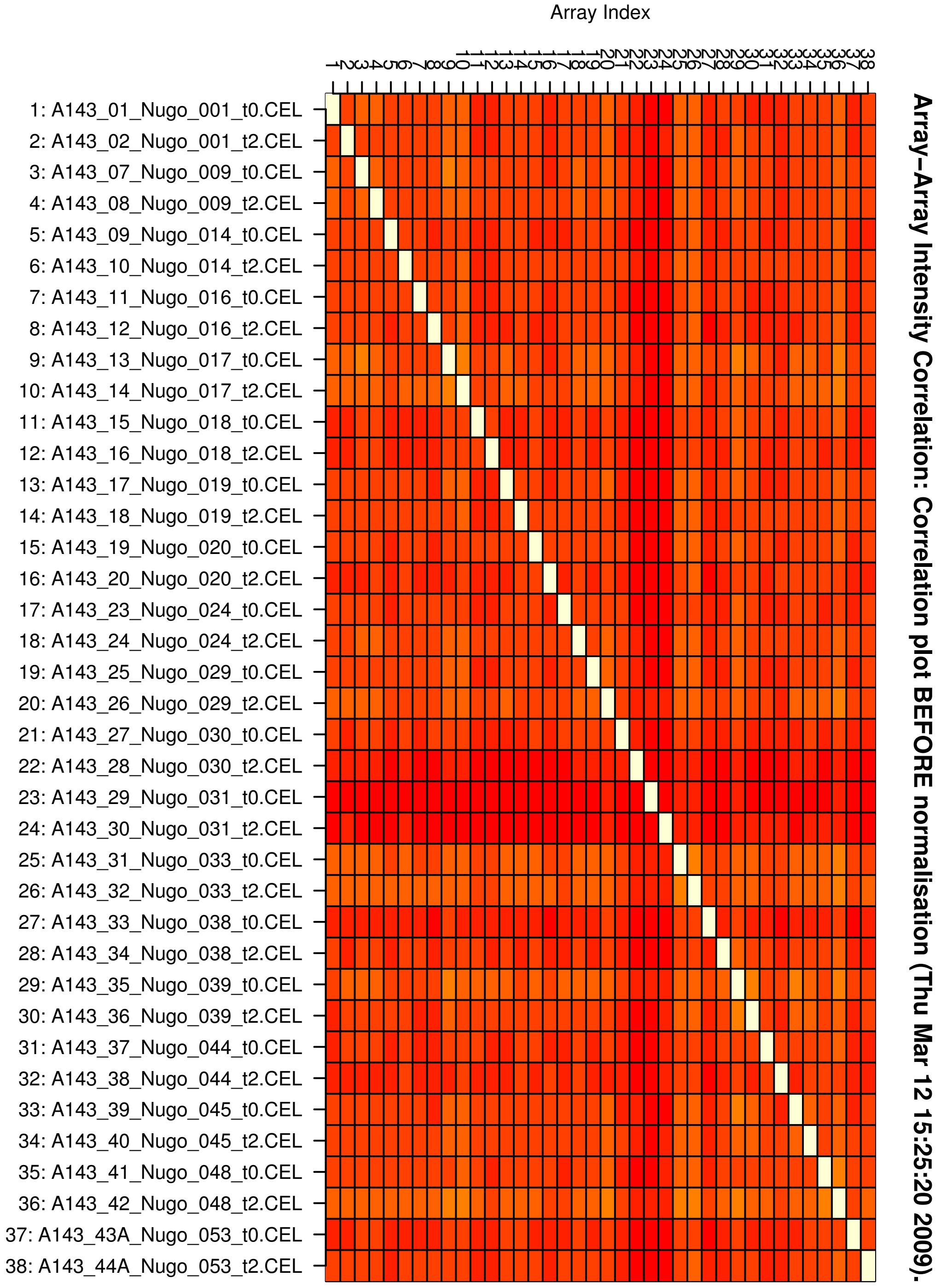
Array Index
1 2 3 4 5 6 7 8 9 10 11 12 13 14 15 16 17 18 19 20 21 22 23 24 25 26 27 28 29 30 31 32 33 34 35 36 37 38
1: A143_01_Nugo_001_t0.CEL
2: A143_02_Nugo_001_t2.CEL
3: A143_07_Nugo_009_t0.CEL
4: A143_08_Nugo_009_t2.CEL
5: A143_09_Nugo_014_t0.CEL
6: A143_10_Nugo_014_t2.CEL
7: A143_11_Nugo_016_t0.CEL
8: A143_12_Nugo_016_t2.CEL
9: A143_13_Nugo_017_t0.CEL
10: A143_14_Nugo_017_t2.CEL
11: A143_15_Nugo_018_t0.CEL
12: A143_16_Nugo_018_t2.CEL
13: A143_17_Nugo_019_t0.CEL
14: A143_18_Nugo_019_t2.CEL
15: A143_19_Nugo_020_t0.CEL
16: A143_20_Nugo_020_t2.CEL
17: A143_23_Nugo_024_t0.CEL
18: A143_24_Nugo_024_t2.CEL
19: A143_25_Nugo_029_t0.CEL
20: A143_26_Nugo_029_t2.CEL
21: A143_27_Nugo_030_t0.CEL
22: A143_28_Nugo_030_t2.CEL
23: A143_29_Nugo_031_t0.CEL
24: A143_30_Nugo_031_t2.CEL
25: A143_31_Nugo_033_t0.CEL
26: A143_32_Nugo_033_t2.CEL
27: A143_33_Nugo_038_t0.CEL
28: A143_34_Nugo_038_t2.CEL
29: A143_35_Nugo_039_t0.CEL
30: A143_36_Nugo_039_t2.CEL
31: A143_37_Nugo_044_t0.CEL
32: A143_38_Nugo_044_t2.CEL
33: A143_39_Nugo_045_t0.CEL
34: A143_40_Nugo_045_t2.CEL
35: A143_41_Nugo_048_t0.CEL
36: A143_42_Nugo_048_t2.CEL
37: A143_43A_Nugo_053_t0.CEL
38: A143_44A_Nugo_053_t2.CEL
Array–Array Intensity Correlation: Correlation plot BEFORE normalisation (Thu Mar 12 15:25:20 2009).

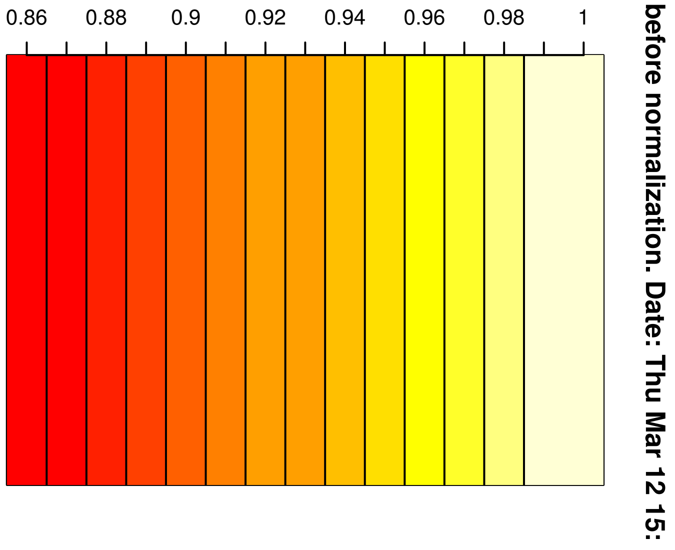
0.86
0.88
0.9
0.92
0.94
0.96
0.98
1
before normalization. Date: Thu Mar 12 15:

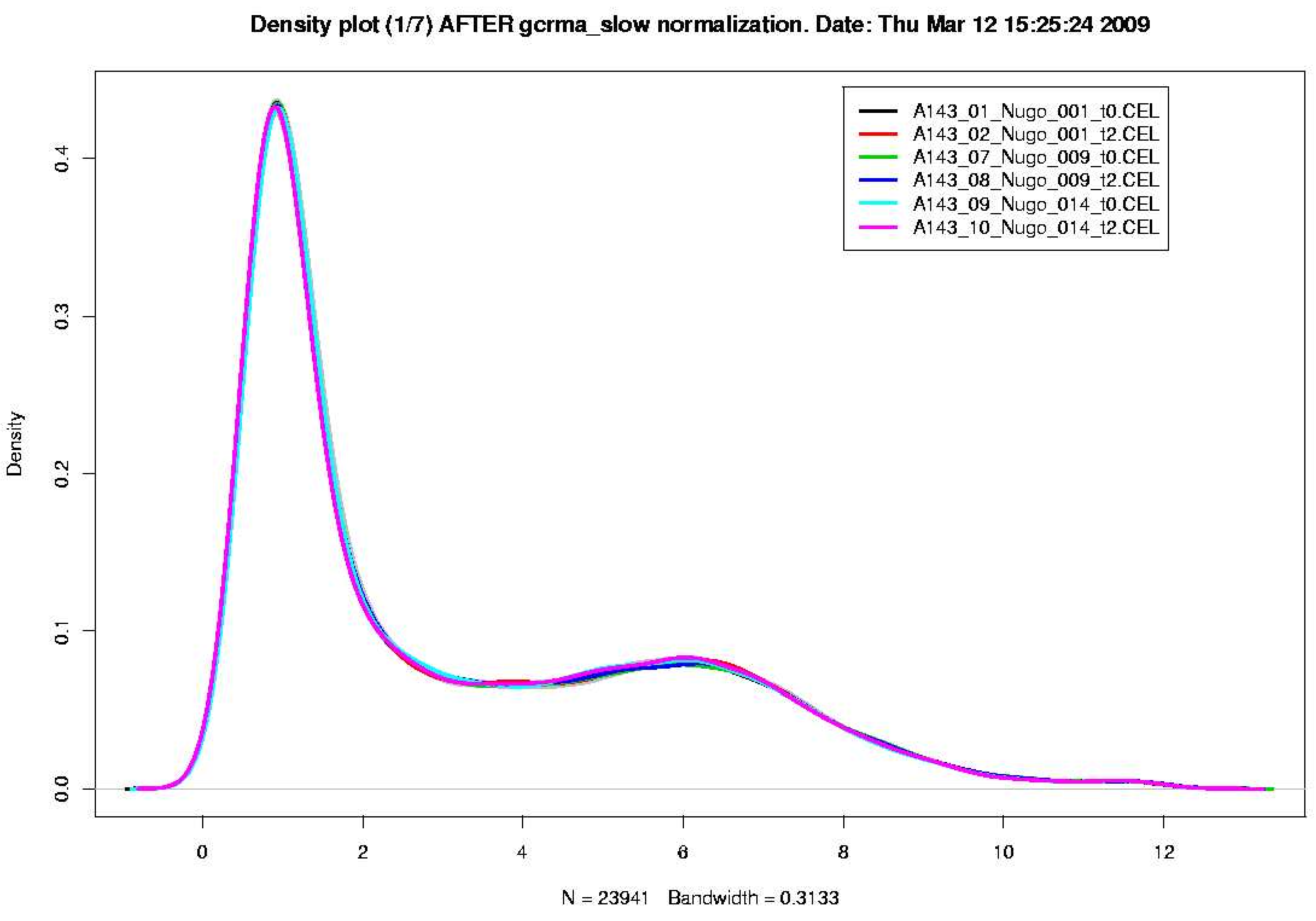
Density plot (1/7) AFTER gcrma_slow normalization. Date: Thu Mar 12 15:25:24 2009
A143_01_Nugo_001_t0.CEL
A143_02_Nugo_001_t2.CEL
A143_07_Nugo_009_t0.CEL
A143_08_Nugo_009_t2.CEL
A143_09_Nugo_014_t0.CEL
A143_10_Nugo_014_t2.CEL
Density
0.0
0.1
0.2
0.3
0.4
0
2
4
6
8
10
12
N = 23941 Bandwidth = 0.3133

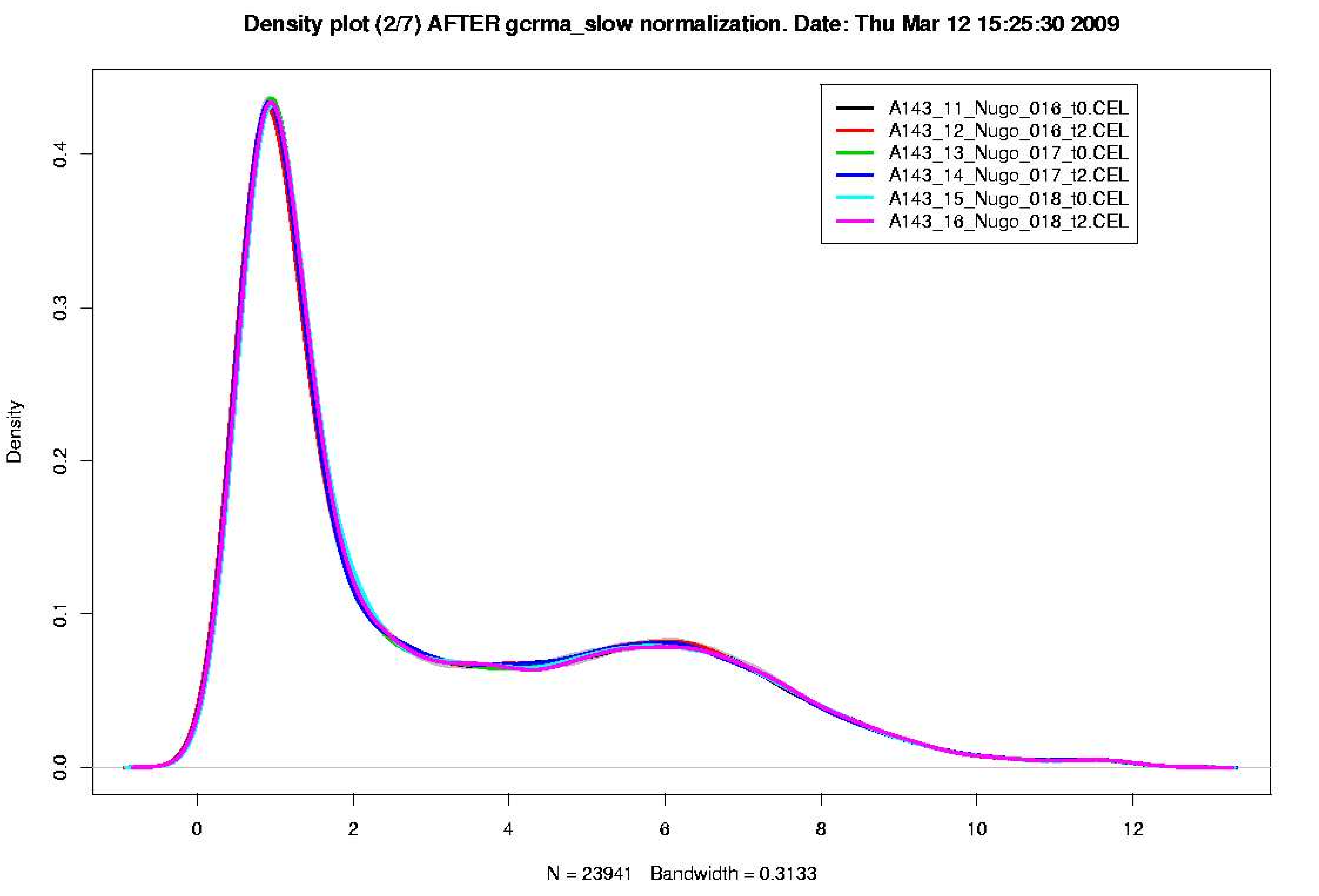
Density plot (2/7) AFTER gcrma_slow normalization. Date: Thu Mar 12 15:25:30 2009
Density
0.0
0.1
0.2
0.3
0.4
0
2
4
6
8
10
12
N = 23941 Bandwidth = 0.3133
A143_11_Nugo_016_t0.CEL
A143_12_Nugo_016_t2.CEL
A143_13_Nugo_017_t0.CEL
A143_14_Nugo_017_t2.CEL
A143_15_Nugo_018_t0.CEL
A143_16_Nugo_018_t2.CEL

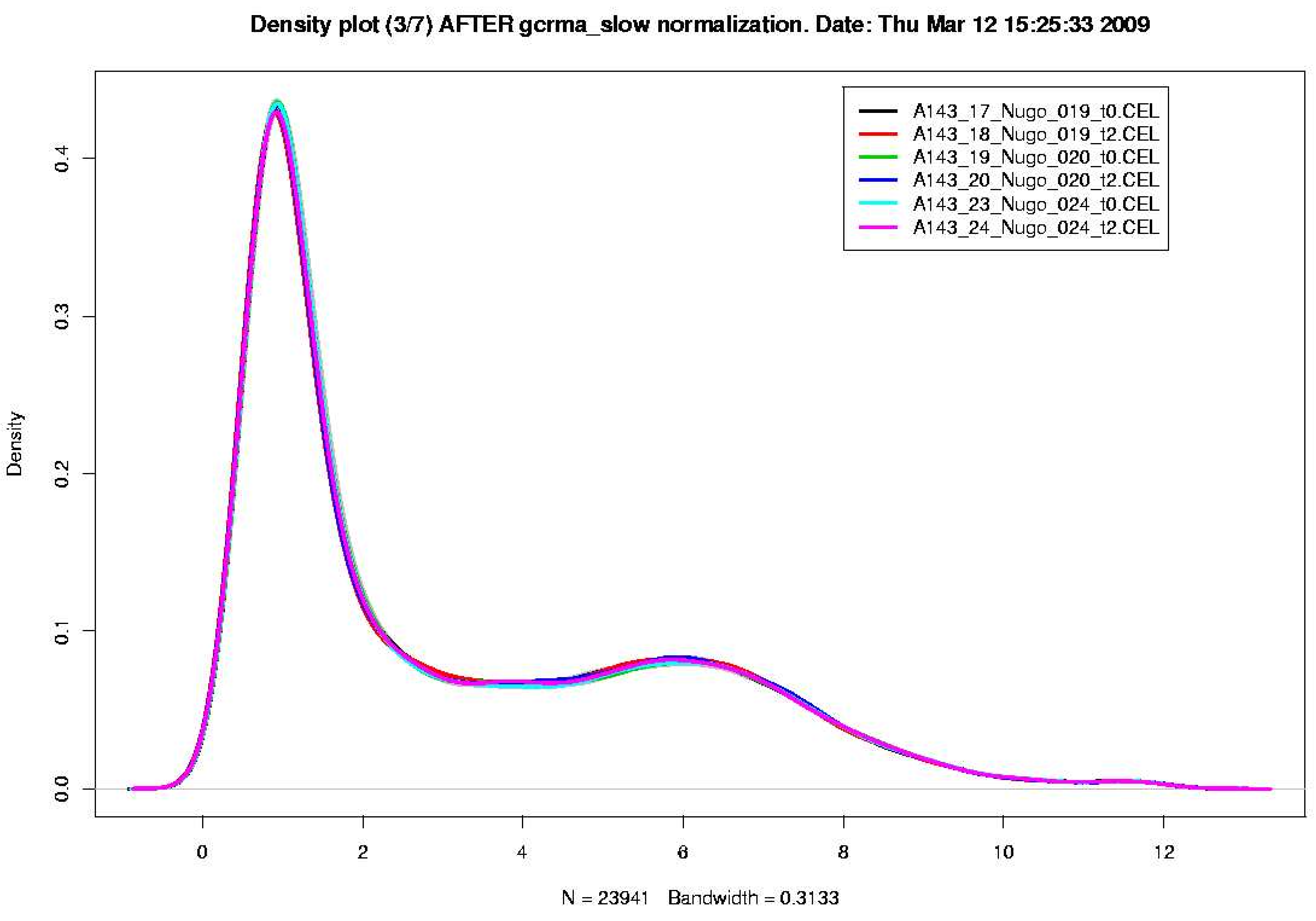
Density plot (3/7) AFTER gcrma_slow normalization. Date: Thu Mar 12 15:25:33 2009
A143_17_Nugo_019_t0.CEL
A143_18_Nugo_019_t2.CEL
A143_19_Nugo_020_t0.CEL
A143_20_Nugo_020_t2.CEL
A143_23_Nugo_024_t0.CEL
A143_24_Nugo_024_t2.CEL
Density
0.0
0.1
0.2
0.3
0.4
0
2
4
6
8
10
12
N = 23941 Bandwidth = 0.3133

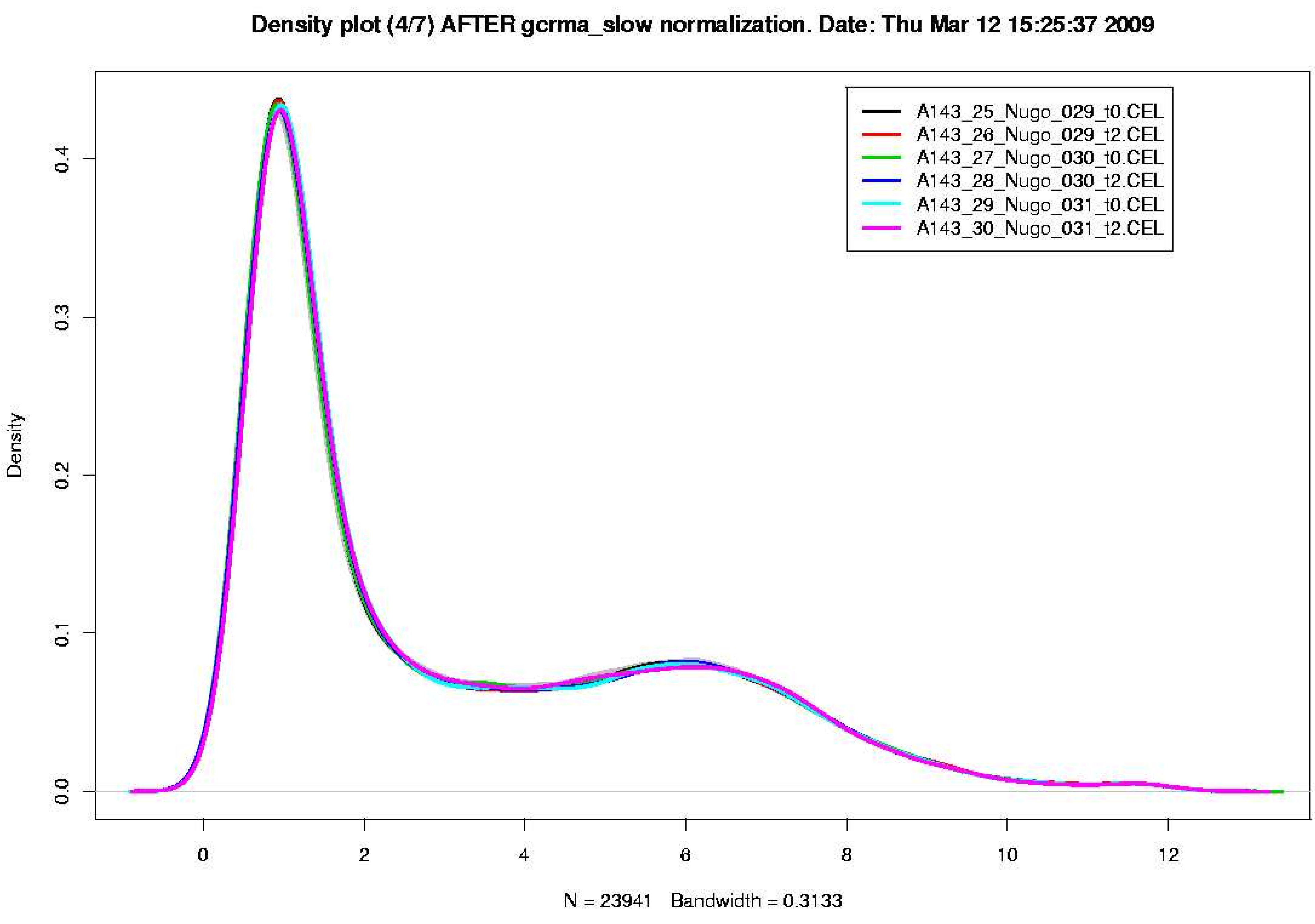
Density plot (4/7) AFTER gcrma_slow normalization. Date: Thu Mar 12 15:25:37 2009
A143_25_Nugo_029_t0.CEL
A143_26_Nugo_029_t2.CEL
A143_27_Nugo_030_t0.CEL
A143_28_Nugo_030_t2.CEL
A143_29_Nugo_031_t0.CEL
A143_30_Nugo_031_t2.CEL
Density
0.0
0.1
0.2
0.3
0.4
0
2
4
6
8
10
12
N = 23941 Bandwidth = 0.3133

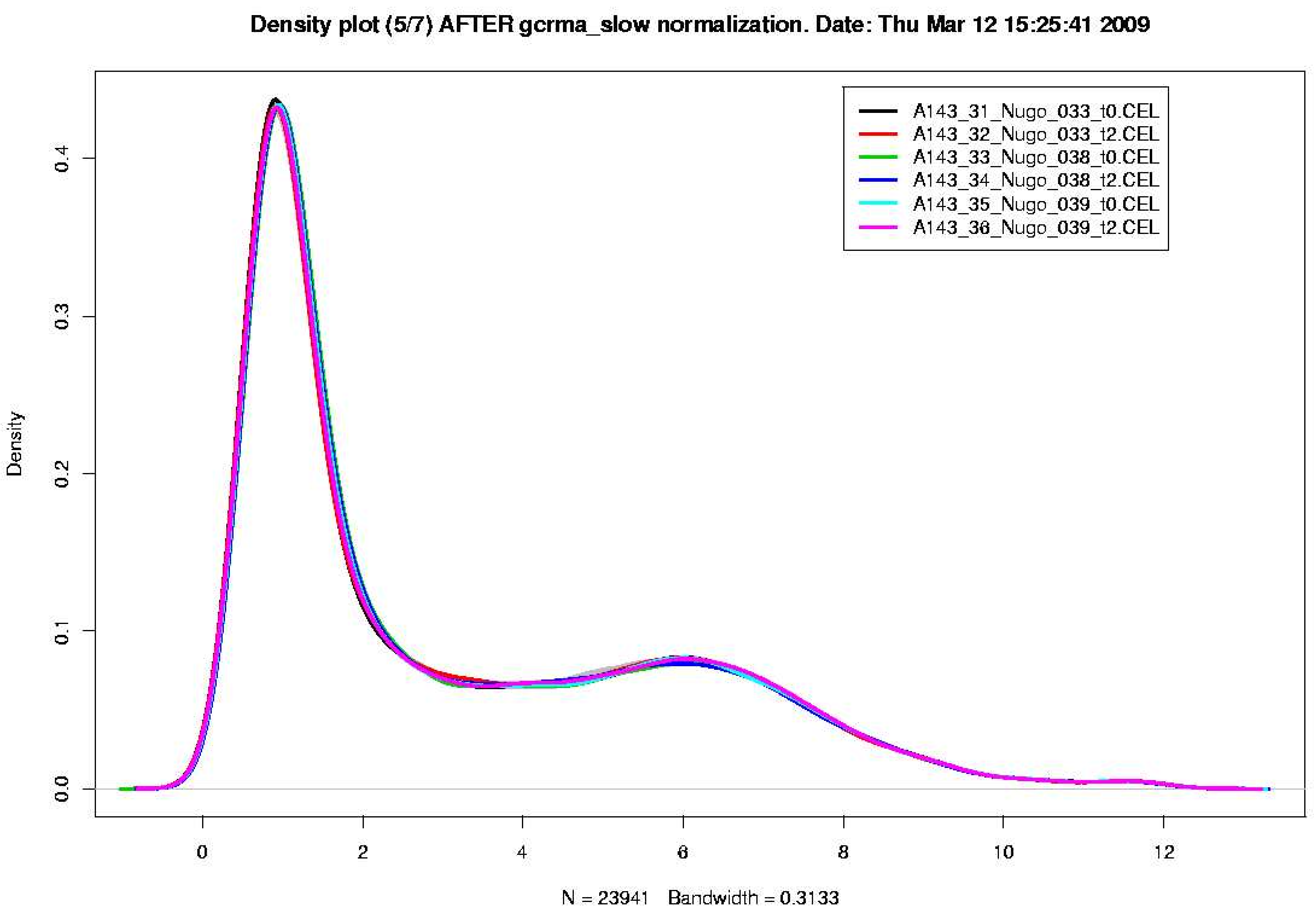
Density plot (5/7) AFTER gcrma_slow normalization. Date: Thu Mar 12 15:25:41 2009
A143_31_Nugo_033_t0.CEL
A143_32_Nugo_033_t2.CEL
A143_33_Nugo_038_t0.CEL
A143_34_Nugo_038_t2.CEL
A143_35_Nugo_039_t0.CEL
A143_36_Nugo_039_t2.CEL
Density
0.0
0.1
0.2
0.3
0.4
0
2
4
6
8
10
12
N = 23941 Bandwidth = 0.3133

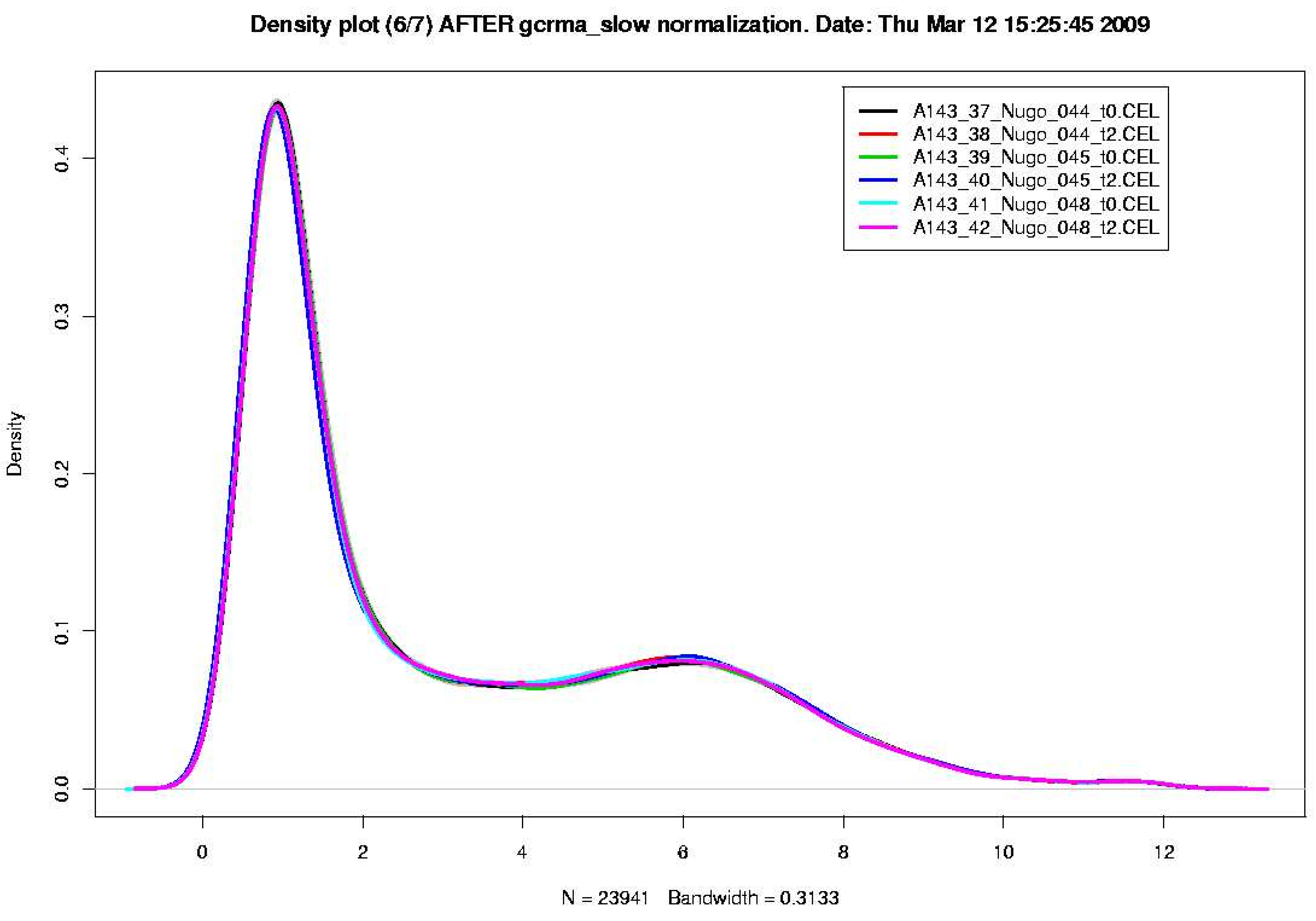
Density plot (6/7) AFTER gcrma_slow normalization. Date: Thu Mar 12 15:25:45 2009
Density
0.0
0.1
0.2
0.3
0.4
0
2
4
6
8
10
12
N = 23941 Bandwidth = 0.3133
A143_37_Nugo_044_t0.CEL
A143_38_Nugo_044_t2.CEL
A143_39_Nugo_045_t0.CEL
A143_40_Nugo_045_t2.CEL
A143_41_Nugo_048_t0.CEL
A143_42_Nugo_048_t2.CEL

Density plot (7/7) AFTER gcrma_slow normalization. Date: Thu Mar 12 15:25:48 2009

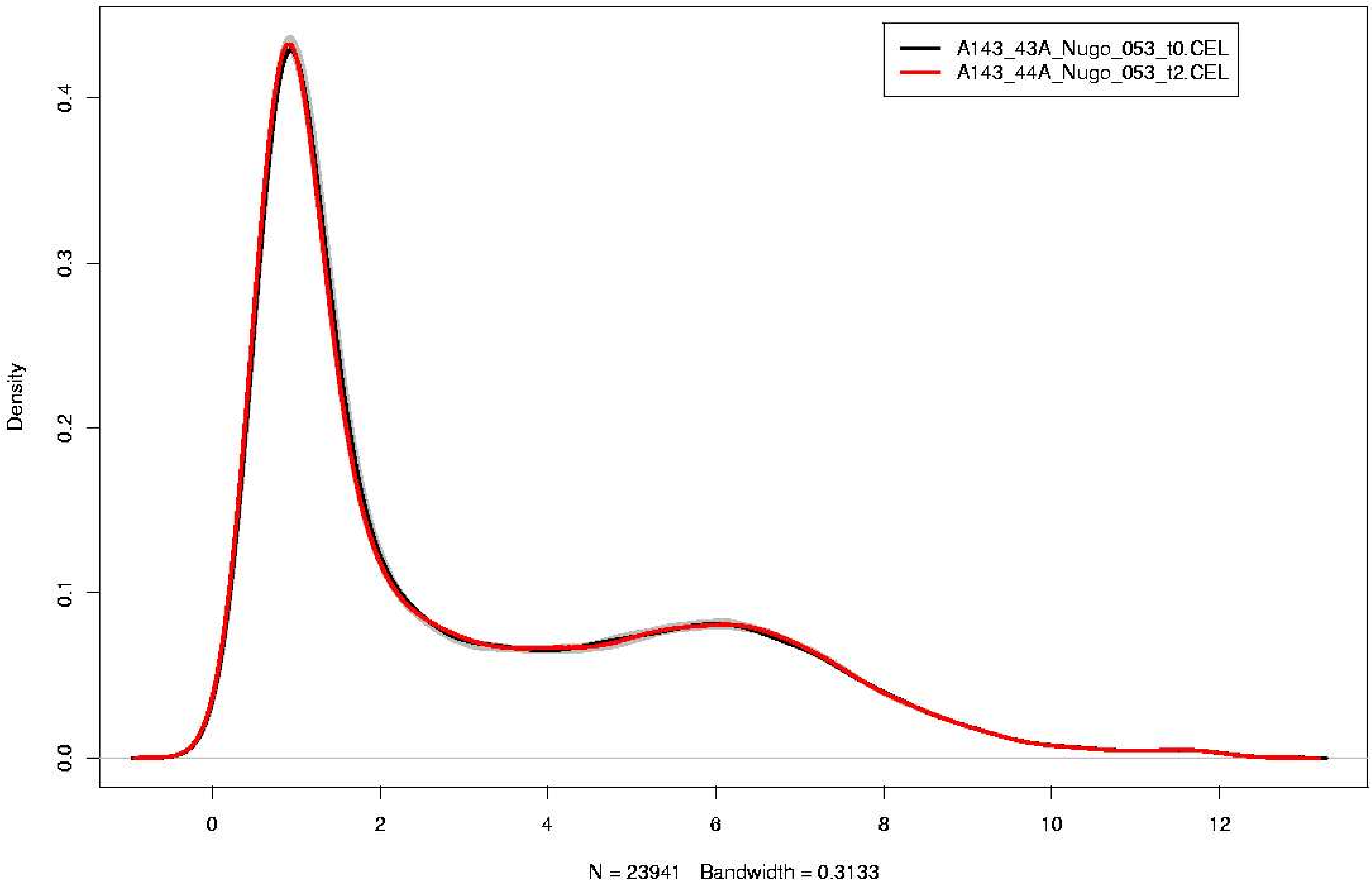

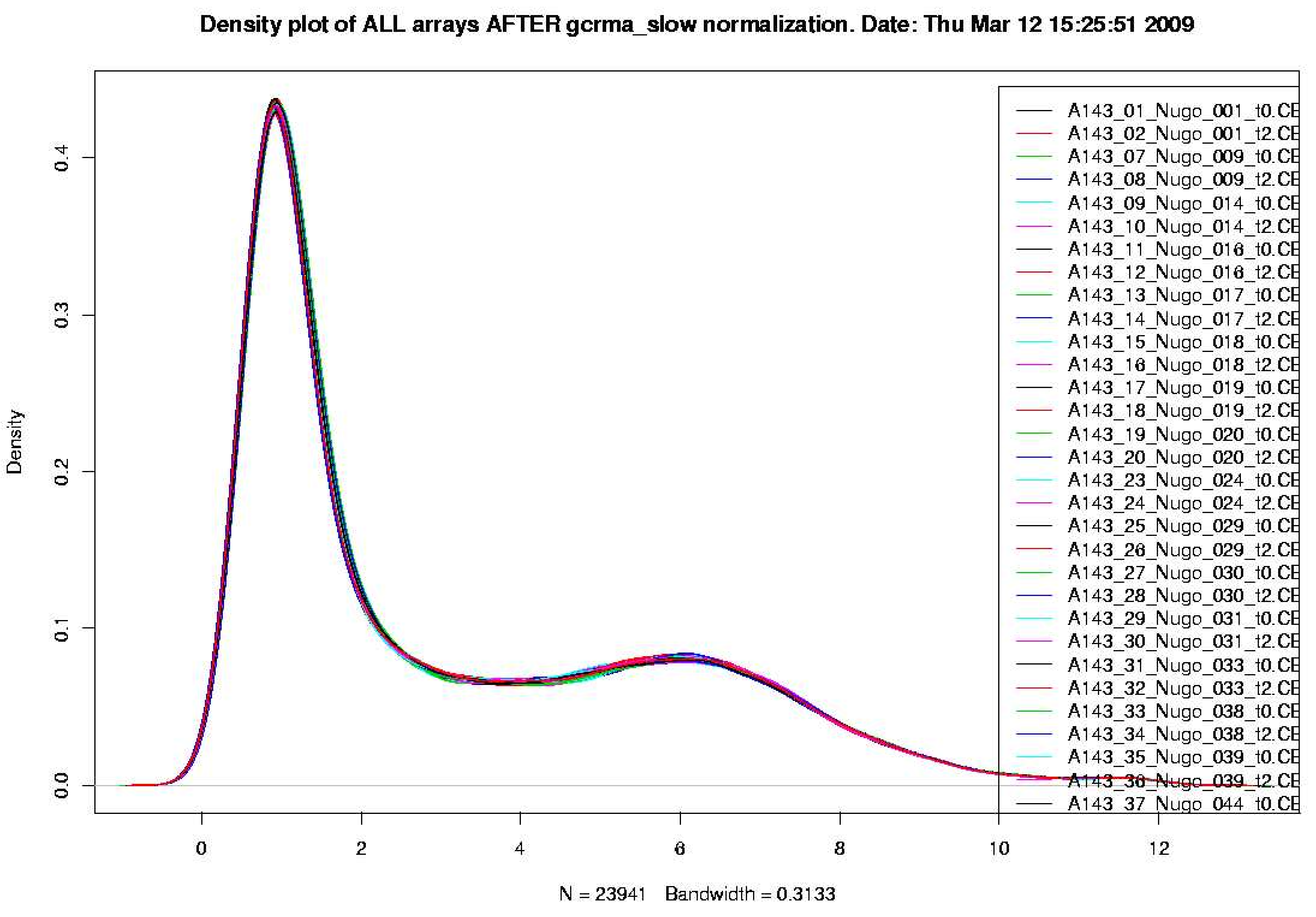
Density plot of ALL arrays AFTER gcrma_slow normalization. Date: Thu Mar 12 15:25:51 2009
Density
0.0
0.1
0.2
0.3
0.4
0
2
4
6
8
10
12
N = 23941 Bandwidth = 0.3133
A143_01_Nugo_001_t0.CE
A143_02_Nugo_001_t2.CE
A143_07_Nugo_009_t0.CE
A143_08_Nugo_009_t2.CE
A143_09_Nugo_014_t0.CE
A143_10_Nugo_014_t2.CE
A143_11_Nugo_016_t0.CE
A143_12_Nugo_016_t2.CE
A143_13_Nugo_017_t0.CE
A143_14_Nugo_017_t2.CE
A143_15_Nugo_018_t0.CE
A143_16_Nugo_018_t2.CE
A143_17_Nugo_019_t0.CE
A143_18_Nugo_019_t2.CE
A143_19_Nugo_020_t0.CE
A143_20_Nugo_020_t2.CE
A143_23_Nugo_024_t0.CE
A143_24_Nugo_024_t2.CE
A143_25_Nugo_029_t0.CE
A143_26_Nugo_029_t2.CE
A143_27_Nugo_030_t0.CE
A143_28_Nugo_030_t2.CE
A143_29_Nugo_031_t0.CE
A143_30_Nugo_031_t2.CE
A143_31_Nugo_033_t0.CE
A143_32_Nugo_033_t2.CE
A143_33_Nugo_038_t0.CE
A143_34_Nugo_038_t2.CE
A143_35_Nugo_039_t0.CE
A143_36_Nugo_039_t2.CE
A143_37_Nugo_044_t0.CE

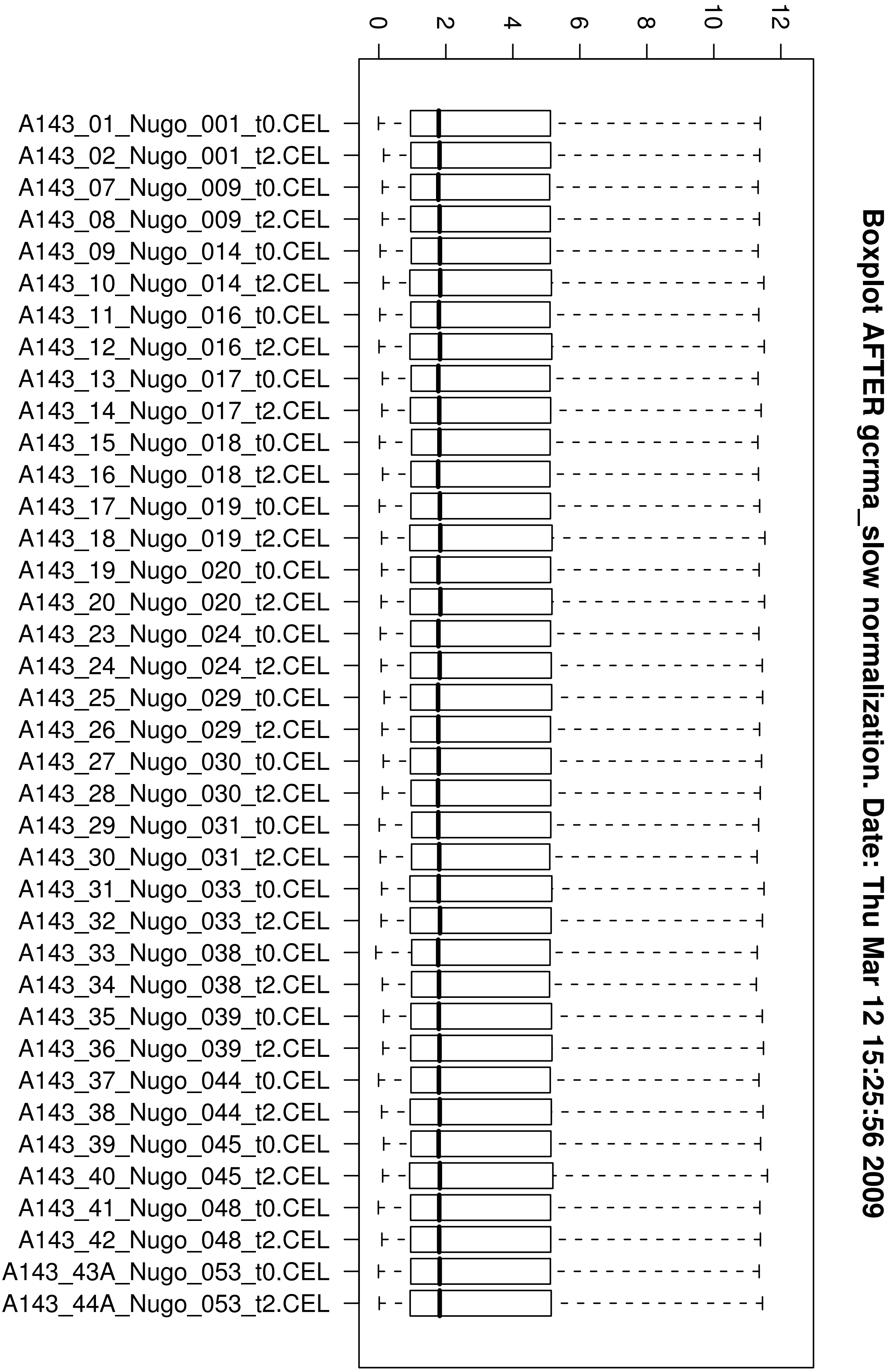

Boxplot AFTER gcrma_slow normalization. Date: Thu Mar 12 15:25:56 2009
0
2
4
6
8
10
12
A143_01_Nugo_001_t0.CEL
A143_02_Nugo_001_t2.CEL
A143_07_Nugo_009_t0.CEL
A143_08_Nugo_009_t2.CEL
A143_09_Nugo_014_t0.CEL
A143_10_Nugo_014_t2.CEL
A143_11_Nugo_016_t0.CEL
A143_12_Nugo_016_t2.CEL
A143_13_Nugo_017_t0.CEL
A143_14_Nugo_017_t2.CEL
A143_15_Nugo_018_t0.CEL
A143_16_Nugo_018_t2.CEL
A143_17_Nugo_019_t0.CEL
A143_18_Nugo_019_t2.CEL
A143_19_Nugo_020_t0.CEL
A143_20_Nugo_020_t2.CEL
A143_23_Nugo_024_t0.CEL
A143_24_Nugo_024_t2.CEL
A143_25_Nugo_029_t0.CEL
A143_26_Nugo_029_t2.CEL
A143_27_Nugo_030_t0.CEL
A143_28_Nugo_030_t2.CEL
A143_29_Nugo_031_t0.CEL
A143_30_Nugo_031_t2.CEL
A143_31_Nugo_033_t0.CEL
A143_32_Nugo_033_t2.CEL
A143_33_Nugo_038_t0.CEL
A143_34_Nugo_038_t2.CEL
A143_35_Nugo_039_t0.CEL
A143_36_Nugo_039_t2.CEL
A143_37_Nugo_044_t0.CEL
A143_38_Nugo_044_t2.CEL
A143_39_Nugo_045_t0.CEL
A143_40_Nugo_045_t2.CEL
A143_41_Nugo_048_t0.CEL
A143_42_Nugo_048_t2.CEL
A143_43A_Nugo_053_t0.CEL
A143_44A_Nugo_053_t2.CEL

RLE plot (gcrma_slow). Date: Thu Mar 12 14:59:54 2009.

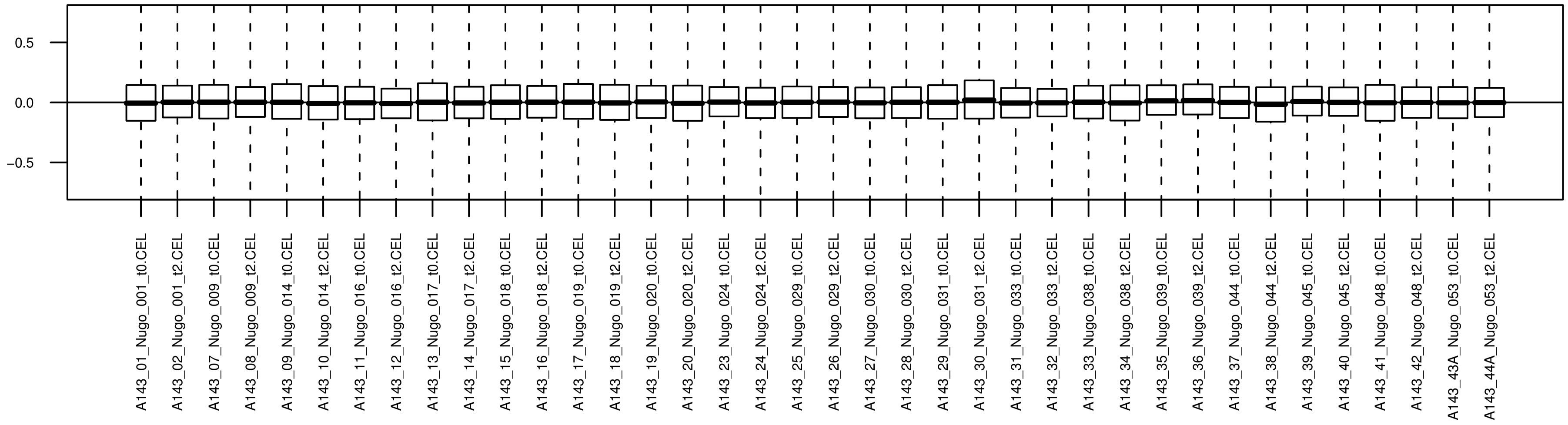

RLE plot (RMA). Date: Thu Mar 12 15:01:29 2009.

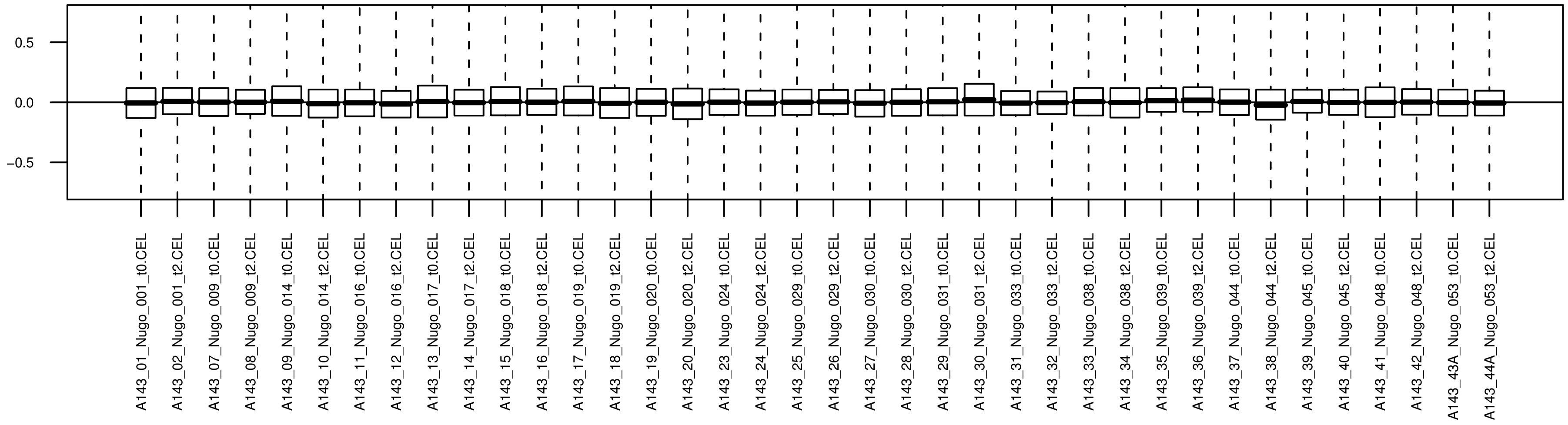

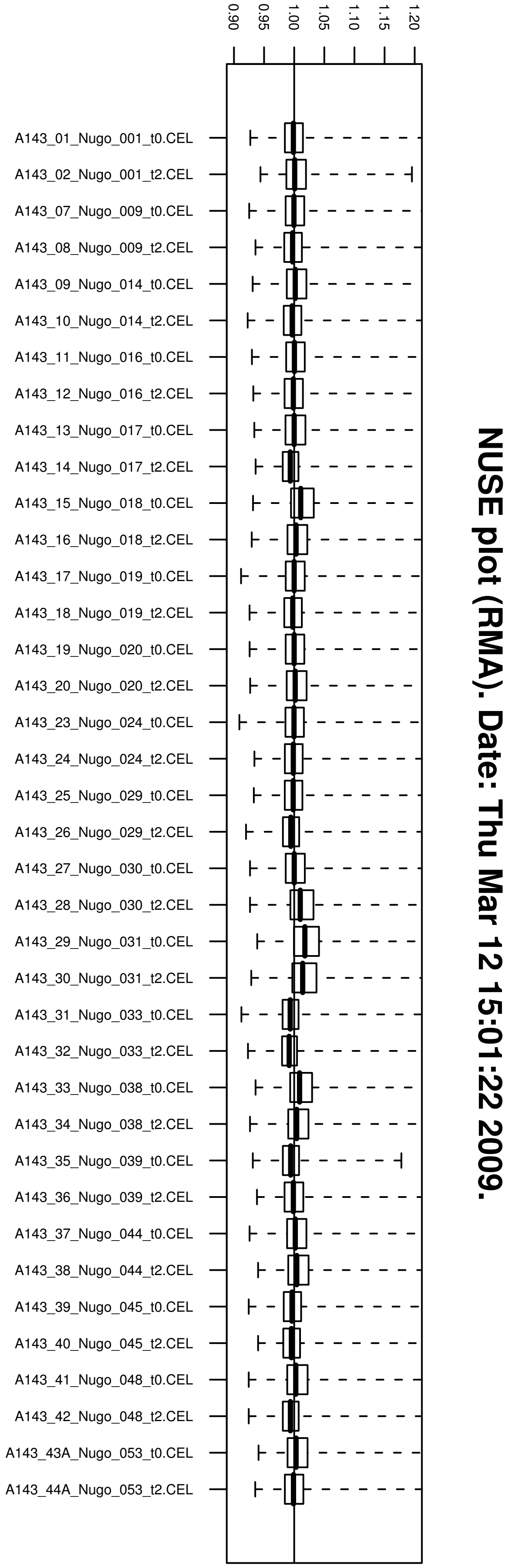
NUSE plot (RMA). Date: Thu Mar 12 15:01:22 2009.
0.90
0.95
1.00
1.05
1.10
1.15
1.20
A143_01_Nugo_001_t0.CEL
A143_02_Nugo_001_t2.CEL
A143_07_Nugo_009_t0.CEL
A143_08_Nugo_009_t2.CEL
A143_09_Nugo_014_t0.CEL
A143_10_Nugo_014_t2.CEL
A143_11_Nugo_016_t0.CEL
A143_12_Nugo_016_t2.CEL
A143_13_Nugo_017_t0.CEL
A143_14_Nugo_017_t2.CEL
A143_15_Nugo_018_t0.CEL
A143_16_Nugo_018_t2.CEL
A143_17_Nugo_019_t0.CEL
A143_18_Nugo_019_t2.CEL
A143_19_Nugo_020_t0.CEL
A143_20_Nugo_020_t2.CEL
A143_23_Nugo_024_t0.CEL
A143_24_Nugo_024_t2.CEL
A143_25_Nugo_029_t0.CEL
A143_26_Nugo_029_t2.CEL
A143_27_Nugo_030_t0.CEL
A143_28_Nugo_030_t2.CEL
A143_29_Nugo_031_t0.CEL
A143_30_Nugo_031_t2.CEL
A143_31_Nugo_033_t0.CEL
A143_32_Nugo_033_t2.CEL
A143_33_Nugo_038_t0.CEL
A143_34_Nugo_038_t2.CEL
A143_35_Nugo_039_t0.CEL
A143_36_Nugo_039_t2.CEL
A143_37_Nugo_044_t0.CEL
A143_38_Nugo_044_t2.CEL
A143_39_Nugo_045_t0.CEL
A143_40_Nugo_045_t2.CEL
A143_41_Nugo_048_t0.CEL
A143_42_Nugo_048_t2.CEL
A143_43A_Nugo_053_t0.CEL
A143_44A_Nugo_053_t2.CEL

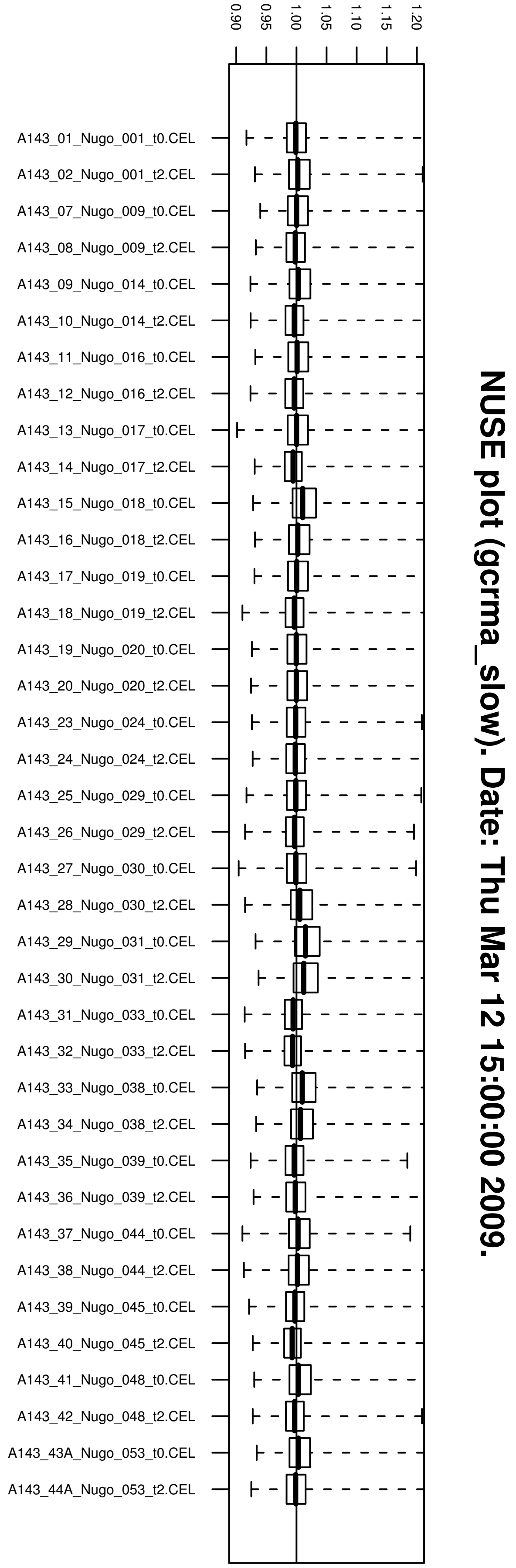
NUSE plot (gcrma_slow). Date: Thu Mar 12 15:00:00 2009.
0.90
0.95
1.00
1.05
1.10
1.15
1.20
A143_01_Nugo_001_t0.CEL
A143_02_Nugo_001_t2.CEL
A143_07_Nugo_009_t0.CEL
A143_08_Nugo_009_t2.CEL
A143_09_Nugo_014_t0.CEL
A143_10_Nugo_014_t2.CEL
A143_11_Nugo_016_t0.CEL
A143_12_Nugo_016_t2.CEL
A143_13_Nugo_017_t0.CEL
A143_14_Nugo_017_t2.CEL
A143_15_Nugo_018_t0.CEL
A143_16_Nugo_018_t2.CEL
A143_17_Nugo_019_t0.CEL
A143_18_Nugo_019_t2.CEL
A143_19_Nugo_020_t0.CEL
A143_20_Nugo_020_t2.CEL
A143_23_Nugo_024_t0.CEL
A143_24_Nugo_024_t2.CEL
A143_25_Nugo_029_t0.CEL
A143_26_Nugo_029_t2.CEL
A143_27_Nugo_030_t0.CEL
A143_28_Nugo_030_t2.CEL
A143_29_Nugo_031_t0.CEL
A143_30_Nugo_031_t2.CEL
A143_31_Nugo_033_t0.CEL
A143_32_Nugo_033_t2.CEL
A143_33_Nugo_038_t0.CEL
A143_34_Nugo_038_t2.CEL
A143_35_Nugo_039_t0.CEL
A143_36_Nugo_039_t2.CEL
A143_37_Nugo_044_t0.CEL
A143_38_Nugo_044_t2.CEL
A143_39_Nugo_045_t0.CEL
A143_40_Nugo_045_t2.CEL
A143_41_Nugo_048_t0.CEL
A143_42_Nugo_048_t2.CEL
A143_43A_Nugo_053_t0.CEL
A143_44A_Nugo_053_t2.CEL

Array–Array Intensity Correlation: Correlation plot AFTER gcrma_slow normalisation (Thu Mar 12 15:28:49 2009).

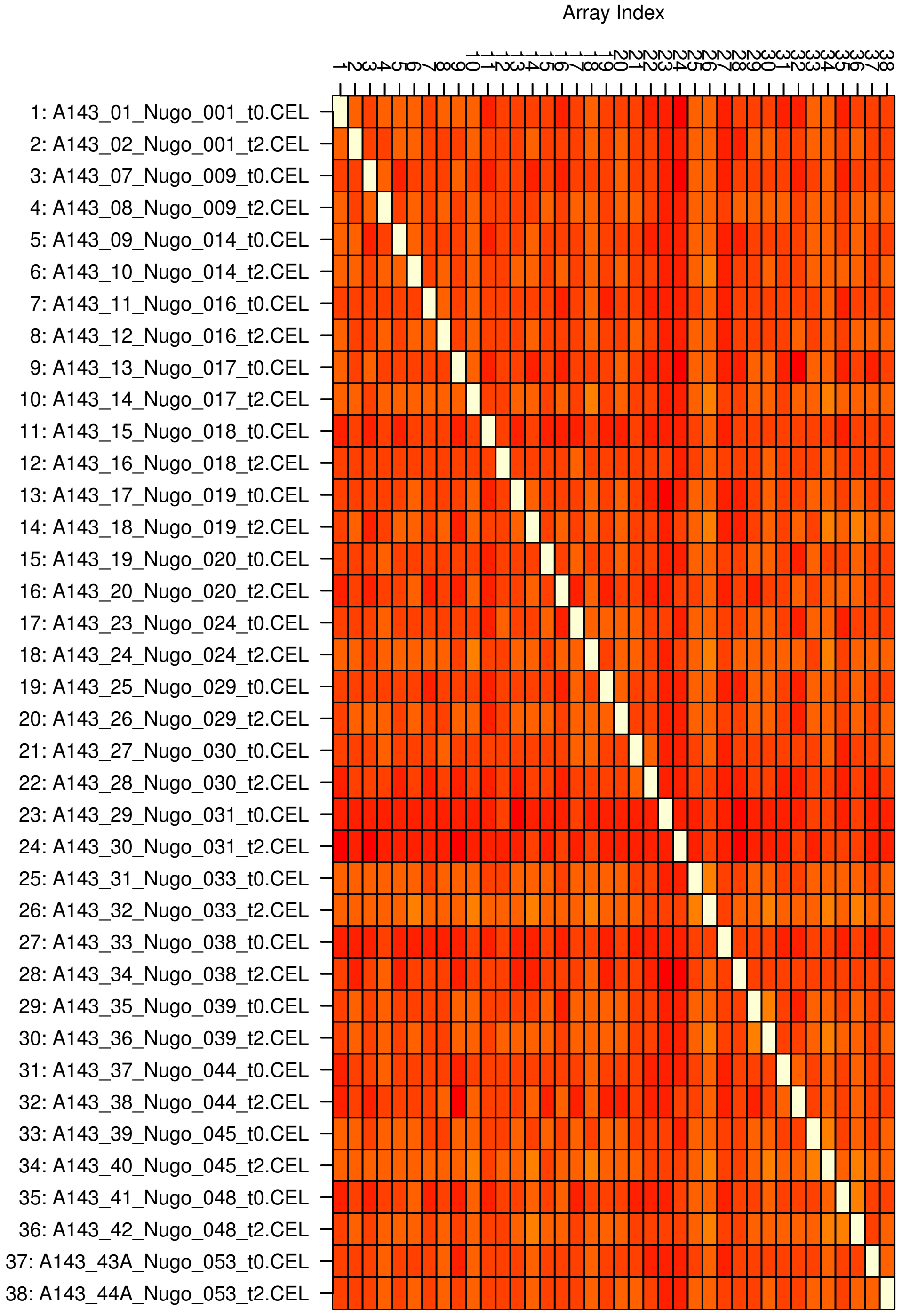

gcrma_slow normalization. Date: Thu Mar

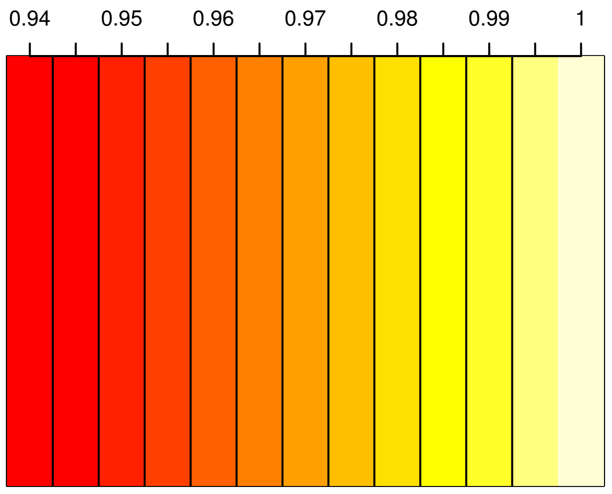

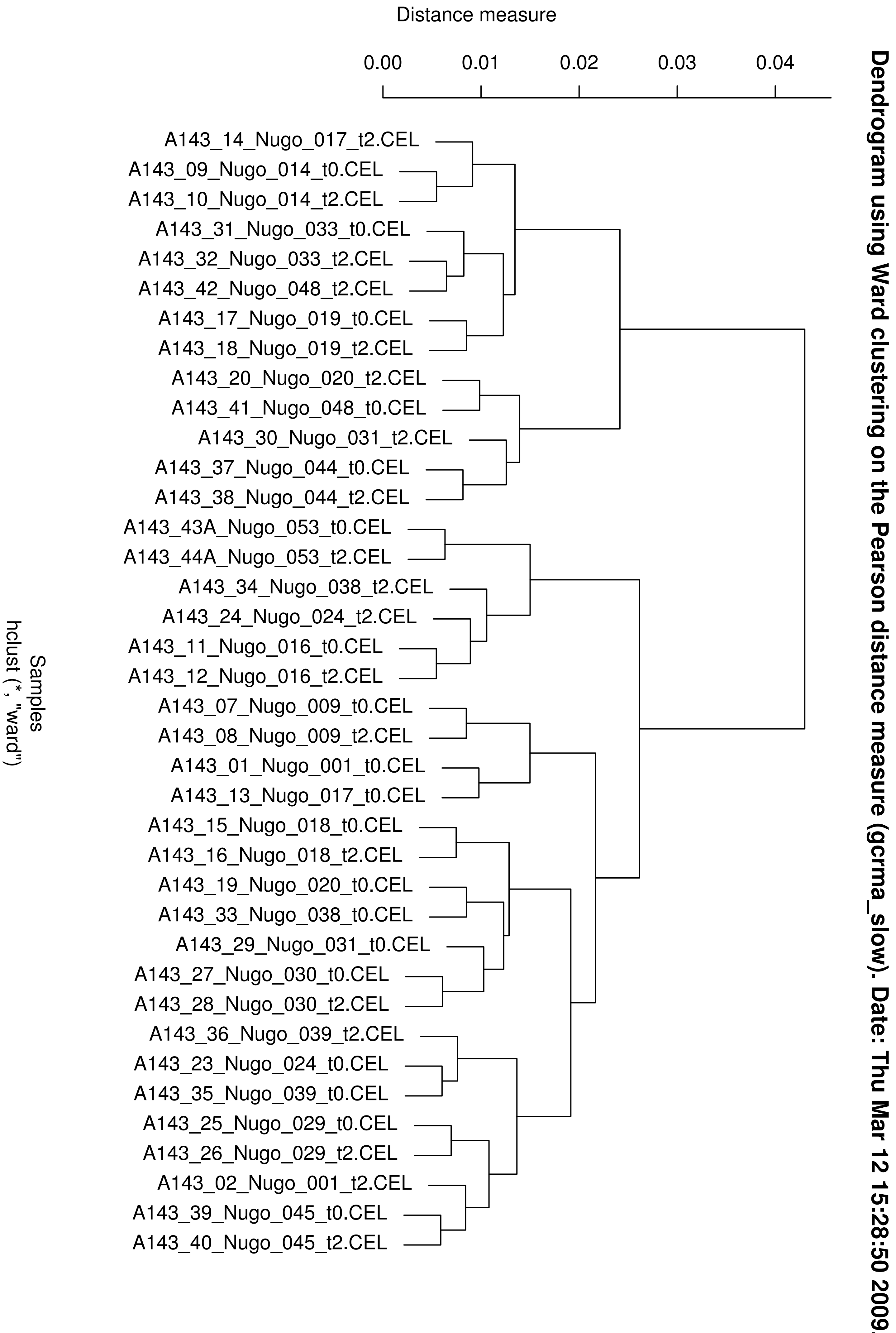
Dendrogram using Ward clustering on the Pearson distance measure (gcrma_slow). Date: Thu Mar 12 15:28:50 2009.
Distance measure
0.00
0.01
0.02
0.03
0.04
A143_14_Nugo_017_t2.CEL
A143_09_Nugo_014_t0.CEL
A143_10_Nugo_014_t2.CEL
A143_31_Nugo_033_t0.CEL
A143_32_Nugo_033_t2.CEL
A143_42_Nugo_048_t2.CEL
A143_17_Nugo_019_t0.CEL
A143_18_Nugo_019_t2.CEL
A143_20_Nugo_020_t2.CEL
A143_41_Nugo_048_t0.CEL
A143_30_Nugo_031_t2.CEL
A143_37_Nugo_044_t0.CEL
A143_38_Nugo_044_t2.CEL
A143_43A_Nugo_053_t0.CEL
A143_44A_Nugo_053_t2.CEL
A143_34_Nugo_038_t2.CEL
A143_24_Nugo_024_t2.CEL
A143_11_Nugo_016_t0.CEL
A143_12_Nugo_016_t2.CEL
A143_07_Nugo_009_t0.CEL
A143_08_Nugo_009_t2.CEL
A143_01_Nugo_001_t0.CEL
A143_13_Nugo_017_t0.CEL
A143_15_Nugo_018_t0.CEL
A143_16_Nugo_018_t2.CEL
A143_19_Nugo_020_t0.CEL
A143_33_Nugo_038_t0.CEL
A143_29_Nugo_031_t0.CEL
A143_27_Nugo_030_t0.CEL
A143_28_Nugo_030_t2.CEL
A143_36_Nugo_039_t2.CEL
A143_23_Nugo_024_t0.CEL
A143_35_Nugo_039_t0.CEL
A143_25_Nugo_029_t0.CEL
A143_26_Nugo_029_t2.CEL
A143_02_Nugo_001_t2.CEL
A143_39_Nugo_045_t0.CEL
A143_40_Nugo_045_t2.CEL
Samples
hclust (*, "ward")
